# Acetic Acid Mediated for One-Pot Synthesis of Novel Pyrazolyl *s*-Triazine Derivatives for the Targeted Therapy of Triple-Negative Breast Tumor Cells (MDA-MB-231) *via* EGFR/PI3K/AKT/mTOR Signaling Cascades

**DOI:** 10.3390/pharmaceutics14081558

**Published:** 2022-07-27

**Authors:** Ihab Shawish, Assem Barakat, Ali Aldalbahi, Walhan Alshaer, Fadwa Daoud, Dana A. Alqudah, Mazhar Al Zoubi, Ma’mon M. Hatmal, Mohamed S. Nafie, Matti Haukka, Anamika Sharma, Beatriz G. de la Torre, Fernando Albericio, Ayman El-Faham

**Affiliations:** 1 Department of Math and Sciences, College of Humanities and Sciences, Prince Sultan University, P.O. Box 66833, Riyadh 11586, Saudi Arabia; ishawish@psu.edu.sa; 2 Department of Chemistry, College of Science, King Saud University, P.O. Box 2455, Riyadh 11451, Saudi Arabia; aaldalbahi@ksu.edu.sa; 3 Cell Therapy Center, The University of Jordan, Amman 11942, Jordan; walhanjordan@yahoo.com (W.A.); fadwadaoud22@gmail.com (F.D.); pharmd.dana.alqudah@gmail.com (D.A.A.); 4 Department of Basic Medical Sciences, Faculty of Sciences, Yarmouk University, Irbid 21163, Jordan; mszoubi@yu.edu.jo; 5 Department of Medical Laboratory Sciences, Faculty of Applied Medical Sciences, The Hashemite University, P.O. Box 330127, Zarqa 13133, Jordan; mamon@hu.edu.jo; 6 Department of Chemistry, Faculty of Science, Suez Canal University, Ismailia 41522, Egypt; mohamed_nafie@science.suez.edu.eg; 7 Department of Chemistry, University of Jyväskylä, P.O. Box 35, FI-40014 Jyväskylä, Finland; matti.o.haukka@jyu.fi; 8 KwaZulu-Natal Research Innovation and Sequencing Platform (KRISP), School of Laboratory Medicine and Medical Sciences, College of Health Sciences, University of KwaZulu-Natal, Durban 4041, South Africa; anamika.aug14@gmail.com (A.S.); garciadelatorreb@ukzn.ac.za (B.G.d.l.T.); 9 Peptide Science Laboratory, School of Chemistry and Physics, University of KwaZulu-Natal, Durban 4001, South Africa; 10 CIBER-BBN (Networking Centre on Bioengineering, Biomaterials and Nanomedicine) and Department of Organic Chemistry, University of Barcelona, 08028 Barcelona, Spain; 11 Institute for Advanced Chemistry of Catalonia (IQAC-CSIC), 08034 Barcelona, Spain; 12 Chemistry Department, Faculty of Science, Alexandria University, P.O. Box 426, Ibrahimia, Alexandria 12321, Egypt

**Keywords:** one-pot synthesis, DMF-DMA, pyrazolyl-*s*-triazine, anticancer profile, EGFR/PI3K/AKT/mTOR, apoptosis

## Abstract

Here, we described the synthesis of novel pyrazole-*s*-triazine derivatives via an easy one-pot procedure for the reaction of β-dicarbonyl compounds (ethylacetoacetate, 5,5-dimethyl-1,3-cyclohexadione or 1,3-cyclohexadionone) with *N*,*N*-dimethylformamide dimethylacetal, followed by addition of 2-hydrazinyl-4,6-disubstituted-*s*-triazine either in ethanol-acetic acid or neat acetic acid to afford a novel pyrazole and pyrazole-fused cycloalkanone systems. The synthetic protocol proved to be efficient, with a shorter reaction time and high chemical yield with broad substrates. The new pyrazolyl-*s*-triazine derivatives were tested against the following cell lines: MCF-7 (breast cancer); MDA-MB-231 (triple-negative breast cancer); U-87 MG (glioblastoma); A549 (non-small cell lung cancer); PANC-1 (pancreatic cancer); and human dermal fibroblasts (HDFs). The cell viability assay revealed that most of the *s*-triazine compounds induced cytotoxicity in all the cell lines tested. However, compounds **7d**, **7f** and **7c**, which all have a piperidine or morpholine moiety with one aniline ring or two aniline rings in their structures, were the most effective. Compounds **7f** and **7d** showed potent EGFR inhibitory activity with IC_50_ values of 59.24 and 70.3 nM, respectively, compared to Tamoxifen (IC_50_ value of 69.1 nM). Compound **7c** exhibited moderate activity, with IC_50_ values of 81.6 nM. Interestingly, hybrids **7d** and **7f** exerted remarkable PI3K/AKT/mTOR inhibitory activity with 0.66/0.82/0.80 and 0.35/0.56/0.66-fold, respectively, by inhibiting their concentrations to 4.39, 37.3, and 69.3 ng/mL in the **7d**-treatment, and to 2.39, 25.34 and 57.6 ng/mL in the **7f**-treatment compared to the untreated control.

## 1. Introduction

Pyrazole derivatives are a highly relevant class of heterocyclic compounds as they are vital substructures in a variety of compounds with important biological properties [1,2,3,4,5]. They have a wide spectrum of activities, including antimicrobial [6,7,8], anti-inflammatory [9], antiparasitic [10], antidepressant [11], antiviral [12], antifungal [13], and antitumor [14,15] activity. Moreover, the pyrazole nucleus is the core unit in numerous FDA-approved pharmaceutical drugs such as celecobix (Celebrex) [16], sildenafil (Viagra) [17], and rimonabant (Acomplia) [18].

Given the excellent bioactivity and wide range of applications of pyrazole derivatives, many studies have addressed their synthesis and bioactivities [19,20]. Furthermore, the methodology for the synthesis of these derivatives has been summarized in several reviews [7,21,22,23].

Enaminediones are widely used to generate polysubstituted pyrazoles [20,24,25,26]. To innovate pharmaceutically relevant pyrazoles, the research community has been attracted to the capacity of enaminediones and 1,2- and 1,3-dinucleophiles to construct diverse heterocycles [2,3,4,5].

On the other hand, many *s*-triazine (1,3,5-triazine) derivatives show a wide range of biological activity [27,28,29,30,31,32,33]. Thus, the synthesis and evaluation of *s*-triazine derivatives coupled with a pyrazolyl ring is a key endeavor in the field. *s*-triazines with the pyrazolyl fragment in their structure can be synthesized by cyclotrimerization of aromatic nitriles [34] or from cyanuric chloride by substitution of the first or second chlorine atom by the aromatic amines containing the pyrazolyl fragment [35]. Ayyangar et al. prepared *s*-triazinylpyrazoles by reacting hydrazinyl-*s*-triazines with 3-iminobutyronitrile and acetoacetic ester [36]. Later, Mikhaylichenko et al. reported the synthesis of 1,3,5-triazine pyrazole derivatives using quaternary amine salts [37].

Recently, we described the synthesis of pyrazole-*s*-triazine derivatives by direct reaction with β-diketone, using triethylamine as a catalyst or using HClO_4_ in an aqueous medium [38,39]. In the present work, we describe a one-pot method for the synthesis of pyrazole and fused pyrazole-*s*-triazine derivatives in the presence of acetic acid *via* the formation of the enaminedione derivatives of β-diketone.

**Figure 1:**
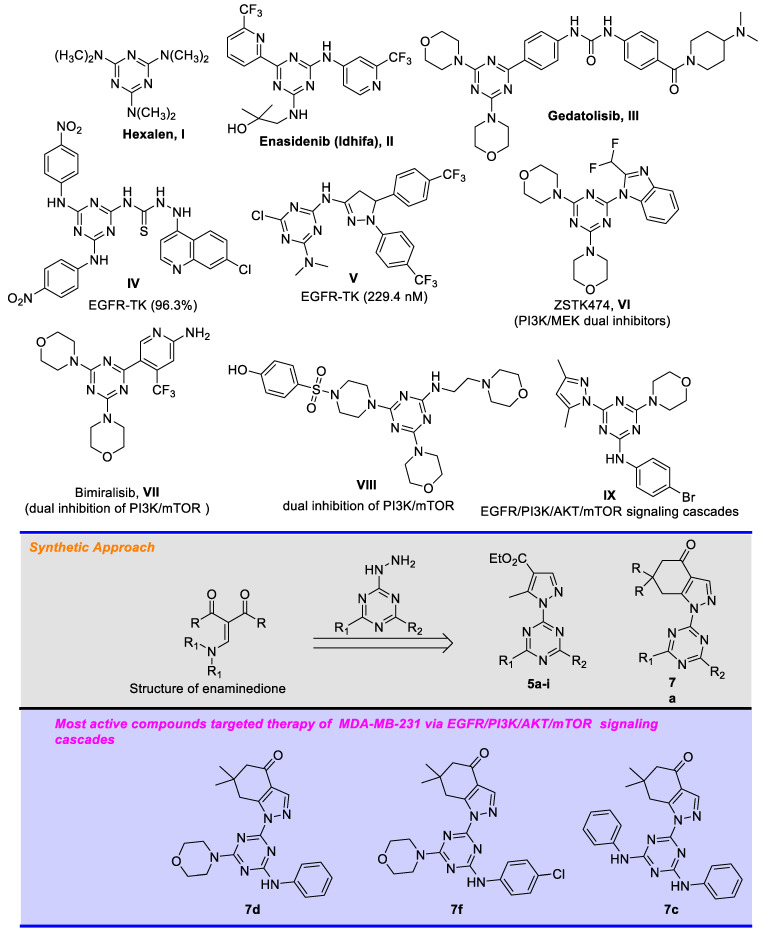
Selected *s*-triazine as an anticancer agent targeting the EGFR/PI3K/AKT/mTOR cascades and our designed compounds.

The search for new compounds with therapeutic efficacy is a major focus in medicinal chemistry. However, the latent progress of resistance or tolerance to these compounds over time, particularly in the context of the treatment of diseases such as cancer, severely limits their medical use. Many representative examples reported for cancer treatment, either approved for human use or in late-stage clinical trials, contain the 1,3,5-triazine (*s*-triazine) moiety. In 1990, the US FDA approved Hexalen (Altretamine) (Figure 1, compound **I**), as an example of targeted therapy for ovarian cancer [40]. First authorized in 2017 by the US-FDA, Enasidenib (Idhifa, compound **II**) is another commercial drug based on the *s*-triazine scaffold and it is used to treat IDH2-positive acute leukemia [41]. Indeed, Gedatolisib was reported as first-in-class to treat breast cancer via the PI3K/mTOR inhibitor (Figure 1, compound **III**) [42]. Several molecules have been reported to be tethered to the *s*-triazine motif, such as targeted EGFR-TK inhibitors **IV** [43] and **V** [44], **VI** ZSTK474 (as PI3K/MEK dual inhibitors) [45], and Bimiralisib (PQR309) (compound **VII**) [46,47,48]. Moreover, **VIII** [49] also shows anti-cancer efficacy as a dual inhibitor of PI3K/mTOR Figure 1). More recently, compound **IX** possessed EGFR/PI3K/AKT/mTOR signaling cascades inhibitor [50] (Figure 1).

In the framework of our ongoing project based on the use of *s*-triazine as a scaffold for the development of novel agents for cancer treatment [38,39], we tested a new series of pyrazolyl-*s*-triazine derivatives against MCF-7, MDA-MB-231, U-87 MG, A549, PANC-1, and HDF cell lines. To better understand the potential mechanism of action of these compounds in human cancer cells, we also conducted an EGFR enzymatic assay and evaluated the PI3K/AKT/mTOR downstream signaling pathway. Finally, a molecular docking study targeting the EGFR/PI3K/AKT/mTOR cascades was conducted.

## 2. Materials and Methods

### 2.1. Chemistry

#### 2.1.1. Materials and Methods

All reagents and solvents were purchased from commercial suppliers and used without further purification. The reaction was followed up and checks of the purity were done using TLC on silica gel-protected aluminum sheets (Type 60 GF254, Merck, Darmstadt, Germany). Melting points were recorded on a Mel-Temp Apparatus (Sigma-Aldrich Chemie GmbH, Taufkirchen, Germany) in an open capillary and are uncorrected. Fourier transform infrared spectroscopy (FTIR) was conducted on a Shimadzu 8201 PC FTIR spectrophotometer (Shimadzu, Ltd., Kyoto, Japan). ^1^H NMR and ^13^C NMR spectra were recorded on a JEOL 400 MHz spectrometer (JEOL, Ltd., Tokyo, Japan), and chemical shift (δ) values were expressed in ppm. Elemental analyses were performed on a Perkin–Elmer 2400 elemental analyzer (PerkinElmer, Inc., Waltham, MA, USA). High resolution mass spectrometry (HRMS) was performed using a Bruker ESI-QTOF mass spectrometer (Bruker, Billerica, MA, USA) in positive-ion mode.

##### General Procedure for the Synthesis of 2-Hydrazino-6-Substituted *s*-Triazine Derivatives, **3a**–**l**

**Scheme 1:**
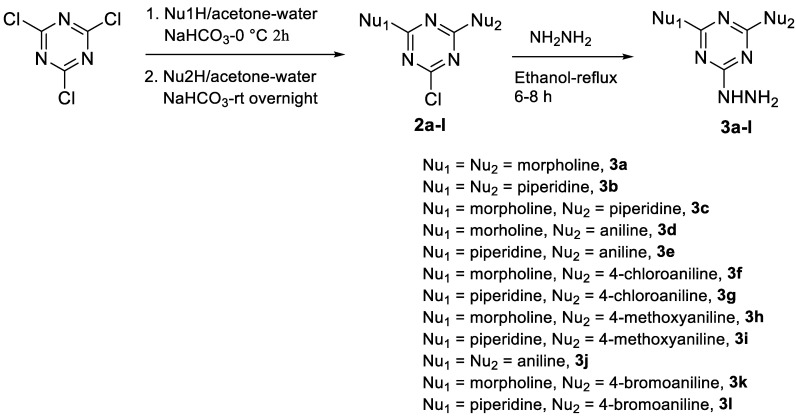
Synthesis of hydrazine-disubstituted *s*-triazine derivatives **3a**–**l**.

A solution of amine (20 mmol) in acetone (50 mL) was added dropwise over 15 min to a solution of cyanuric chloride **1** (20 mmol) in acetone (50 mL) at 0–5 °C. After complete addition, an aqueous solution of NaHCO_3_, (22 mmol equiv.) in water (50 mL) was added dropwise (10 min) at the same temperature. The reaction mixture was then stirred at 0–5 °C for 2 h. After completion of the reaction and disappearance of the starting materials (TLC, ethyl acetate/hexane 2:8), the second nucleophile (20 mmol) in acetone (50 mL) was added at the same temperature, followed by addition of an aqueous solution of NaHCO_3_ (22 mmol equiv.) in water (50 mL). The reaction mixture was stirred at 0 °C for 1 h and then at rt overnight. Excess distilled water was added and the precipitates of products **2a**–**l** (Scheme 1) were collected by filtration, washed with water (2 × 20 mL), and dried at rt to afford the desired products in good yield.

The chloro derivatives **2a**–**l** were reacted with excess hydrazine hydrate (80%) for 6–8 h following the reported method [32,33,50] to afford the desired products **3a**–**l** (Scheme 1) as white solids, which were used directly in the next step.

The spectral data for compounds **2a**–**l** were previously reported by our group [32,33,50] and agreed with the reported data. 

##### General Procedure for the Synthesis of **5a**–**i**

**Scheme 2:**
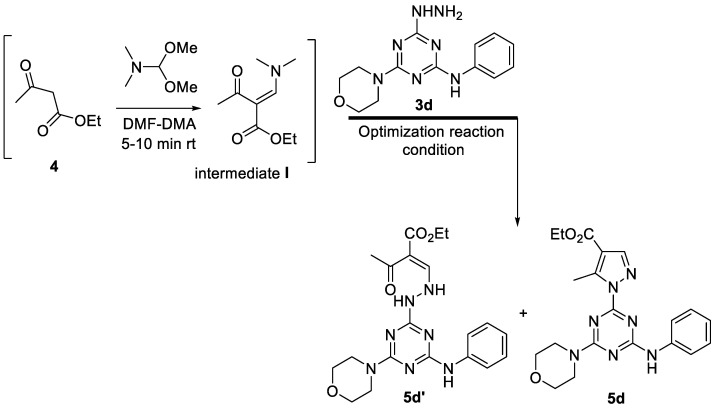
Proposed final products **5d’** and **5d** for the reaction of compound **3d** with ethylacetocacetate and DMF-DMA.

**Scheme 3:**
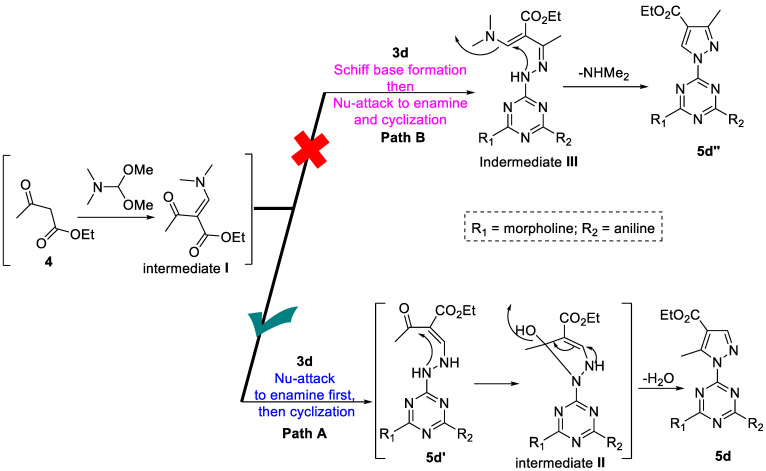
Proposed mechanism for the formation of pyrazole-*s*-triazine derivative **5d** as a model substrate.

**Scheme 4:**
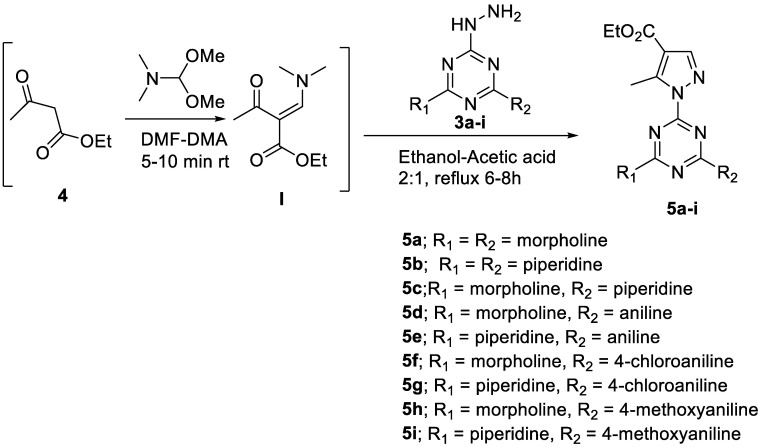
Synthesis of pyrazole-*s*-triazine derivatives **5a**–**i**.

A solution of ethylacetoacetate **4** (1.0 mmol) and *N*,*N*-dimethylformamide dimethylacetal (1.2 mmol) was stirred for 5 min at rt and then 2,4-disubstituted-*s*-triazine derivatives **3a**–**i** (Scheme 1) (1.0 mmol) in ethanol-AcOH (2:1; 10 mL) were slowly added to the mixture. The reaction mixture was refluxed for 6–8 h. The progress of reactions was monitored by TLC (methanol-CHCl_3_; 1:9 or ethylacetate-hexane; 1:1). After completion of the reaction, the solvent was evaporated under reduced pressure, and water (20 mL) was added to the residue, then extracted with ethylacetate (2 × 10 mL). The organic layer was successively washed with sodium carbonate solution and water and then dried with MgSO_4_. Evaporation of the solvent afforded target pyrazole derivatives **5a**–**i** (Scheme 2, Scheme 3 and Scheme 4). The products were recrystallized from DCM-Petroleum ether 40–60.

1. Ethyl 1-(4,6-dimorpholino-1,3,5-triazin-2-yl)-5-methyl-1*H*-pyrazole-4-carboxylate, **5a**

Pale yellow solid in 81% yield, mp 158–159 °C. 1H NMR (400 MHz, CDCl_3_, ppm) δ 1.34 (t, 3H, *J* = 7.2 Hz, CH_3_), 2.92 (s, 3H, CH_3_), 3.71. (brs, 8H, CH_2_-N-CH_2_), 3.82 (brs, 8H, CH_2_-O-CH_2_), 4.31 (q, 2H, *J* = 7.2 Hz, CH_2_), 8.03 (s, 1H, CH-*_pyrazole_*); ^13^C NMR (101 MHz, CDCl_3_, ppm) δ 14.1, 14.3, 43.9, 60.2, 66.7, 114.7, 143.0, 146.9, 163.5, 165.2. Anal. Calc. for C_18_H_25_N_7_O_4_ (403.44) C, 53.59; H, 6.25; N, 24.30. Found C, 53.35; H, 6.16; N, 24.52. HRMS-ESI (*m*/*z*) calculated for [M + H]^+^ 404.44; found: 404.2034.

2. Ethyl 1-(4,6-di(piperidin-1-yl)-1,3,5-triazin-2-yl)-5-methyl-1*H*-pyrazole-4-carboxylate, **5b**

Light brown solid in 80% yield, mp 134–136 °C. ^1^H NMR (400 MHz, CDCl_3_, ppm) δ 1.32 (t, 3H, *J* = 7.2 Hz, CH_3_), 1.57 (brs, 8H, 4CH_2_), 1.64 (brs, 4H, 2CH_2_), 2.92 (s, 3H, CH_3_), 3.76 (brs, 4H, CH_2_-N-CH_2_), 4.29 (q, 2H, *J* = 7.2 Hz, CH_2_), 8.05 (s, 1H, CH *_pyrazole_*); ^13^C NMR (100 MHz, CDCl_3_, ppm) δ 13.9, 14.2, 24.7, 25.7, 44.5, 59.9, 114.3, 142.5, 146.7, 163.4, 163.6, 164.8. Anal. Calc. for C_20_H_29_N_7_O_2_ (399.50) C, 60.13; H, 7.32; N, 24.54. Found C, 60.34; H, 7.44; N, 24.71. HRMS-ESI (*m*/*z*) calculated for [M + H]^+^ 400.50; found: 400.2452.

3. Ethyl 5-methyl-1-(4-morpholino-6-(piperidin-1-yl)-1,3,5-triazin-2-yl)-1*H*-pyrazole-4-carboxylate, **5c**

Light brown solid in 84% yield, mp 119–120 °C. ^1^H NMR (400 MHz, CDCl_3_, ppm) δ 1.33 (t, 3H, *J* = 7.2 Hz, CH_3_), 1.50–1.65 (m, 6H, 3CH_2_), 2.91 (s, 3H, CH_3_), 3.69–3.77 (m, 12H, 6CH_2_), 4.26 (q, 2H, *J* = 7.6 Hz, CH_2_), 8.00 (s, 1H, CH- *_pyrazole_*); ^13^C NMR (101 MHz, CDCl_3_, ppm) δ 14.0, 14.3, 24.6, 25.7, 43.7, 44.6, 60.0, 66.8, 114.4, 142.7, 146.7, 163.5, 164.6, 165.5. Anal. Calc. for C_19_H_27_N_7_O_3_ (401.47) C, 56.84; H, 6.78; N, 24.42. Found C, 56.66; H, 6.62; N, 24.67. HRMS-ESI (*m*/*z*) calculated for [M + H]^+^ 402.47; found: 402.2255.

4. Ethyl 5-methyl-1-(4-morpholino-6-(phenylamino)-1,3,5-triazin-2-yl)-1*H*-pyrazole-4-carboxylate, **5d**

Beige solid in 85% yield, mp 135–137 °C; ^1^H NMR (400 MHz, CDCl_3_, ppm) δ 1.33 (t, 3H, *J* = 7.2 Hz, CH_3_), 2.97 (s, 3H, CH_3_), 3.76 (brs, 4H, CH_2_-N-CH_2_), 3.85 (brs, 4H, CH_2_-O-CH_2_), 4.31 (q, 2H, *J* = 7.2 Hz, CH_2_), 7.09 (t, 1H, *J* = 7.2 Hz, Ar-H), 7.33 (t, 2H, *J* = 7.2 Hz, Ar-H), 7.34 (brs, 1H, NH), 7.62 (t, 2H, *J* = 7.2Hz, Ar-H), 8.09 (s, 1H, CH- *_pyrazole_*); ^13^C NMR (101 MHz, CDCl_3_, ppm) δ 14.3, 44.2, 66.5, 99.9, 117.4, 120.8, 128.9, 138.2, 143.1, 147.2, 155.9, 163.4, 164.2. Anal. Calc. for C_20_H_23_N_7_O_3_ (409.45) C, 58.67; H, 5.66; N, 23.95. Found C, 58.90; H, 5.81; N, 24.19. HRMS-ESI (*m*/*z*) calculated for [M + H]^+^ 410.45; found: 410.2265.

5. Ethyl 5-methyl-1-(4-(phenylamino)-6-(piperidin-1-yl)-1,3,5-triazin-2-yl)-1*H*-pyrazole-4-carboxylate, **5e**

Beige solid in 86% yield, 117–119 °C; ^1^H NMR (400 MHz, CDCl_3_, ppm) δ 1.39 (t, 3H, *J* = 7.2 Hz, CH_3_), 1.67 (brs,6H, 3CH_2_), 2.90 (s, 3H, CH_3_), 3.8 (brs, 4H, CH_2_-N-CH_2_), 4.31 (q, 2H, *J* = 7.2 Hz, CH_2_), 7.09 (t, 1H, *J* = 7.2 Hz, Ar-H), 7.33 (t, 2H, *J* = 7.2 Hz, Ar-H), 7.34 (brs, 1H, NH), 7.62 (t, 2H, *J* = 7.2Hz, Ar-H), 7.87 (brs, 1H, NH), 8.03 (s, 1H, CH-*_pyrazole_*); ^13^C NMR (101 MHz, CDCl_3_, ppm) δ 14.3, 24.5, 25.7, 45.1, 60.2, 114.9, 120.2, 128.8, 138.2, 143.1, 147.2, 163.4, 164.2. Anal. Calc. for C_21_H_25_N_7_O_2_ (407.48): C, 61.90; H, 6.18; N, 24.06. Found C, 61.75; H, 6.23; N, 24.28. HRMS-ESI (*m*/*z*) calculated for [M + H]^+^ 408.48; found:408.3255.

6. Ethyl 1-(4-((4-chlorophenyl)amino)-6-morpholino-1,3,5-triazin-2-yl)-5-methyl-1*H*-pyrazole-4-carboxylate, **5f**

Pale yellow solid in 87% yield, mp 190–191 °C. ^1^H NMR (400 MHz, CDCl_3_, ppm) δ 1.35 (t, 3H, *J* = 7.2 Hz, CH_3_), 2.96 (s, 3H, CH_3_), 3.76 (t, 4H, *J* = 3.6 Hz, CH_2_-N-CH_2_), 3.85 (t, 4H, *J* = 4.4Hz, CH_2_-O-CH_2_), 4.28 (q, 2H, *J* = 7.2 Hz, CH_2_), 7.26 (d, 2H, *J* = 8.8 Hz, Ar-H), 7.47 (d, 2H, *J* = 8.8 Hz, Ar-H), 7.58 (brs, 1H, NH), 8.03 (s, 1H, CH*-_pyrazole_*); ^13^C NMR (101 MHz, CDCl_3_, ppm) δ 14.3, 44.2, 60.2, 66.5, 115.1, 121.6, 128.9, 136.7, 143.2, 147.2, 163.3, 164.5, 165.2. Anal. Calc. for C_20_H_22_ClN_7_O_3_ (443.15) C, 54.12; H, 5.00; Cl, 7.99; N, 22.09. Found C, 54.30; H, 5.13; N, 22.31. HRMS-ESI (*m*/*z*) calculated for [M + H]^+^ 444.15; found: 444.1551.

7. Ethyl 1-(4-((4-chlorophenyl)amino)-6-(piperidin-1-yl)-1,3,5-triazin-2-yl)-5-methyl-1*H*-pyrazole-4-carboxylate, **5g**

Beige solid in 89% yield, mp 140–142 °C; ^1^H NMR (400 MHz, CDCl_3_, ppm) δ 1.34 (t, 3H, *J* = 7.2 Hz, CH_3_), 1.62 (brs, 4H, 2CH_2_), 1.69 (brs, 2H, CH_2_), 2.97 (s, 3H, CH_3_), 3.80 (brs, 4H, CH_2_-N-CH_2_), 4.29 (q, 2H, *J* = 7.6 Hz, CH_2_),7.27 (dd, 2H, *J* = 3.6, 4.0 Hz, Ar-H), 7.38 (brs, 1H, N-H), 7.48 (d, 2H, *J* = 8.02, Ar-H), 8.03 (s, 1H, CH *_pyrazole_*); ^13^C NMR (101 MHz, CDCl_3_, ppm) δ: 14.1, 14.3, 24.5, 25.6, 45.1, 60.2, 114.8, 121.2, 128.3, 128.8, 137.0, 142.9, 147.1, 153.6, 157.6, 162.2, 163.4, 164.6. Anal. Calc. for C_21_H_24_ClN_7_O_2_ (441.92) C, 57.08; H, 5.47; N, 22.19. Found C, 57.21; H, 5.60; N, 22.34. HRMS-ESI (*m*/*z*) calculated for [M + H]^+^ 442.92; found:442.9225.

8. Ethyl 1-(4-((4-methoxyphenyl)amino)-6-morpholino-1,3,5-triazin-2-yl)-5-methyl-1*H*-pyrazole-4-carboxylate, **5h**

Beige solid in 82% yield, mp158–160 °C; ^1^H NMR (400 MHz, CDCl_3_, ppm) δ: 1.32 (t, 3H, *J* = 7.2 Hz, CH_3_), 2.95 (s, 3H, CH_3_), 3.75 (m, 4H, CH_2_-N-CH_2_), 3.78 (s, 3H, OCH_3_), 3.82 (brs, 4H, CH_2_-O-CH_2_),4.28 (q, 2H, *J* = 7.6 Hz, CH_2_), 6.85 (d, 2H, *J* = 9.2 Hz, Ar-H), 7.43 (d, 2H, *J* = 8.0 Hz, Ar-H), 8.03 (s, 1H, CH-*_pyrazole_*), 8.10 (brs, 1H, NH); ^13^C NMR (101 MHz, CDCl_3_, ppm) δ: 14.1,14.7, 44.0, 55.4, 60.2, 66.51, 113.9, 114.9, 122.2, 142.9, 146.7, 163.5, 165.5, 176.1. Anal. Calc. for C_21_H_25_N_7_O_4_ (439.48): C, 57.39; H, 5.73; N, 22.31. Found C, 57.55; H, 5.86; N, 22.18. HRMS-ESI (*m*/*z*) calculated for [M + H]^+^ 440.48; found:440.4556.

9. Ethyl 1-(4-((4-methoxyphenyl)amino)-6-(piperidin-1-yl)-1,3,5-triazin-2-yl)-5-methyl-1*H*-pyrazole-4-carboxylate, **5i**

Beige solid in 84% yield, mp 116–118 °C; ^1^H NMR (400 MHz, CDCl_3_, ppm) δ 1.35 (t, 3H, *J* = 7.2 Hz, CH_3_), 1.62 (brs,4H, 2CH_2_), 1.67 (brs, 2H, CH_2_), 2.96 (s, 3H, CH_3_), 3.77 (brs, 4H, CH_2_-N-CH_2_), 3.79 (s, 3H, OCH_3_), 4.29 (q, 2H, *J* = 7.2 Hz, CH_2_), 6.87 (d, 2H, *J* = 8.6 Hz, Ar-H), 7.43 (brs, 1H, NH), 7.46 (d, 2H, *J* = 8.8 Hz, Ar-H), 8.03 (s, 1H, CH*-_pyrazole_*); ^13^C NMR (101 MHz, CDCl_3_, ppm) δ: 13.99, 14.04, 24.6, 25.7, 44.9, 55.4, 60.1, 113.9, 114.7, 122.1, 131.5, 142.8, 146.9, 162.0, 163.5, 165.5, 175.7. Anal. Calc. for C_22_H_27_N_7_O_3_ (437.50): C, 60.40; H, 6.22; N, 22.41. Found: C, 60.57; H, 6.35; N, 22.67. HRMS-ESI (*m*/*z*) calculated for [M + H]^+^ 438.50; found:438.2255.

##### General Procedure for the Synthesis of *s*-Triazine Derivatives **7a**–**t**

**Scheme 5:**
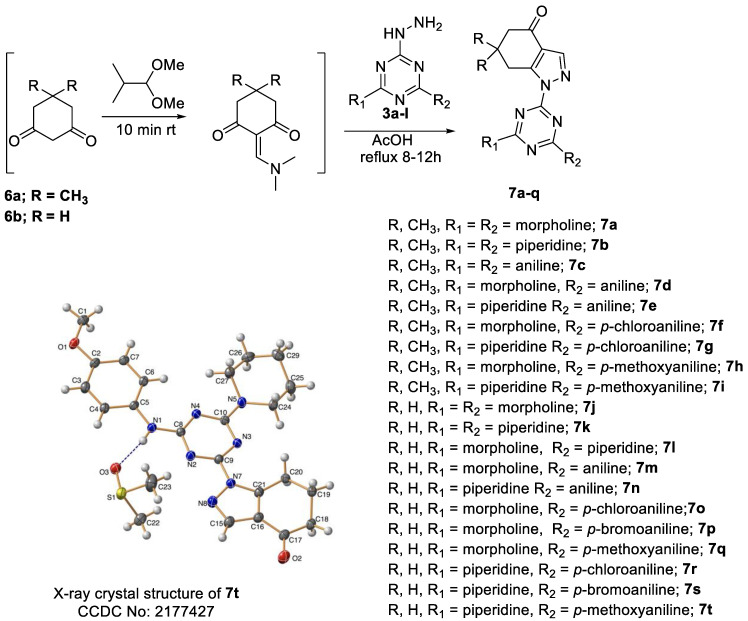
Synthesis of fused pyrazole-*s*-triazine derivatives **7a**–**t**.

A neat mixture of *N*,*N*-dimethylformamide dimethyl acetal (1.2 mmol) and 5,5-dimethyl-1,3-cyclohexadione **6a** or 1,3-cyclohexadione **6b** (Scheme 5) were mixed together and stirred for 10 min at rt. The hydrazine derivatives **3a**–**l** (1.0 mmol) in glacial acetic acid (10 mL) were slowly added to the mixture. The reaction mixture was refluxed for 8–12 h, and its progress was monitored by TLC (methanol-CHCl3; 1:9 or ethyl acetate-hexane, 1:1. After completion of the reaction, the mixture was left to cool to rt and then poured into ice-cold water (50 mL). The aq. solution was extracted with ethyl acetate, washed 10% Na_2_CO_3_ solution, and water several times, then dried (Na_2_SO_4_) to afford the target products **7a**–**t** which were recrystallized from ethyl acetate to give the pure products (Scheme 5).

1. 1-(4,6-Dimorpholino-1,3,5-triazin-2-yl)-6,6-dimethyl-1,5,6,7-tetrahydro-4*H*-indazol-4-one, **7a**

Light brown solid in 80% yield, mp 196–198 °C. ^1^H NMR (400 MHz, CDCl_3_, ppm) δ: 1.09 (s, 6H, 2CH_3_), 2.39 (s, 2H, CH_2_), 3.2 (s, 2H, CH_2_), 3.73 (brs, 8H,2 CH_2_-N-CH_2_), 3.83 (brs, 8H, 2 CH_2_-O-CH_2_), 8.08 (s, 1H, CH-*_pyrazole_*); ^13^C NMR (101 MHz, CDCl_3_, ppm) δ: 28.5, 39.8, 43.6, 43.8, 51.8, 66.7, 120.9, 139.6, 151.5, 162.9, 164.6, 165.1, 193.0. Anal. Calc. for C_20_H_27_N_7_O_3_ (413.48) C, 58.10; H, 6.58; N, 23.71. Found C, 58.24; H, 6.71; N, 23.53. HRMS-ESI (*m*/*z*) calculated for [M + H]^+^ 414.48; found: 414.2246.

2. 1-(4,6-Di(piperidin-1-yl)-1,3,5-triazin-2-yl)-6,6-dimethyl-1,5,6,7-tetrahydro-4*H*-indazol-4-one, **7b**

Off-white solid in 83% yield, mp 176–177 °C. ^1^H NMR (400 MHz, CDCl_3_, ppm) δ 1.08 (s, 6H, 2CH_3_), 1.65 (brs, 12H, 6CH_2_), 2.38 (s, 2H, CH_2_), 3.22 (brs, 2H, CH_2_), 3.81 (brs, 8H, 4CH_2;_ CH_2_-N- CH_2_), 8.12 (s, 1H, CH*-_pyrazole_*); ^13^C NMR (101 MHz, CDCl_3_, ppm) δ 24.6, 25.8, 28.5, 35.3, 40.0, 45.0, 51.8, 120.1, 139.6, 151.5, 162.3, 163.7, 193.1 (CO). Anal. Calc. for C_22_H_31_ClN_7_O (409.54) C, 64.52; H, 7.63; N, 23.94. Found C, C, 64.72; H, 7.81; N, 23.65. HRMS-ESI (*m*/*z*) calculated for [M + H]^+^ 410.54; found: 410.2664.

3. 1-(4,6-*Bis*(phenylamino)-1,3,5-triazin-2-yl)-6,6-dimethyl-1,5,6,7-tetrahydro-4*H*-indazol-4-one, **7c**

Light brown solid in 80% yield, mp 227–229 °C; ^1^H NMR (400 MHz, CDCl_3_, ppm) δ 1.09 (brs, 6H, 2CH_3_), 2.35 (brs, 2H, CH_2_), 3.06-3.34 (brs, 2H, CH_2_), 7.16 (brs, 2H, Ar-H), 7.33 (brs, 4H, Ar-H), 7.56 (brs, 4H, Ar-H), 8.07 (s, 1H, CH*-_pyrazole_*), 8.19 (brs, 1H, NH); ^13^CNMR (101 MHz, CDCl_3_, ppm) δ 28.5, 35.2, 39.6, 51.7, 114.0, 121.1, 123.4, 124.2, 129.2, 140.2, 152.3, 163.3, 175.2, 192.8 (CO). Anal. Calc. for C_24_H_23_N_7_O (425.50): C, 67.75; H, 5.45; N, 23.04. Found: C, C, 67.98; H, 5.67; N, 23.30. HRMS-ESI (*m*/*z*) calculated for [M + H]^+^ 426.50; found: 426.2039.

4. 6,6-Dimethyl-1-(4-morpholino-6-(phenylamino)-1,3,5-triazin-2-yl)-1,5,6,7-tetrahydro-4*H*-indazol-4-one, **7d**

Off-white solid in 80% yield, mp 236–238 °C. ^1^H NMR (400 MHz, CDCl_3_, ppm) δ: 1.06 (s, 6H, 2CH_3_), 2.39 (s, 2H, CH_2_), 3.24 (s, 2H, CH_2_), 3.77 (brs, 4H, 2CH_2;_ CH_2_-N-CH_2_), 3.88 (brs, 4H, 2CH_2_; CH_2_-O-CH_2_), 7.10 (t, 1H, *J* = 7.6, Ar-H), 7.34 (t, 2H, *J* = 8.0, Ar-H), 7.7.53 (t, 2H, *J* = 7.6, Ar-H), 8.11 (s, 1H, CH*-_yrazole_*); ^13^C NMR (101 MHz, CDCl_3_, ppm) δ: 28.5, 35.3, 39.7, 44.2, 51.8, 66.5, 120.4, 121.1, 128.1, 138.1, 139.6, 141.7, 151.6, 162.7, 164.3, 165.2, 193.0 (CO). Anal. Calc. for C_22_H_25_N_7_O_2_ (419.49) C, 62.99; H, 6.01; N, 23.37. Found C, 62.73; H, 6.11; N, 23.54. HRMS-ESI (*m*/*z*) calculated for [M + H]^+^ 420.49; found:420.3225.

5. 6,6-Dimethyl-1-(4-(phenylamino)-6-(piperidin-1-yl)-1,3,5-triazin-2-yl)-1,5,6,7-tetrahydro-4*H*-indazol-4-one, **7e**

Off-white solid in 86% yield, mp 230–232 °C. 1H NMR (400 MHz, CDCl_3_, ppm) δ 1.10 (s, 6H, 2CH_3_), 1.64 (brs, 4H, 2CH_2_), 1.71 (brs, 2H, CH_2_), 2.39 (s, 2H, CH_2_), 3.26 (s, 2H, CH_2_), 3.84 (brs, 4H, 2CH_2;_ CH_2_-N-CH_2_), 7.06 (t, 1H, *J* = 7.6 Hz, Ar-H), 7.31 (t, 2H, *J* = 8.0, Ar-H), 7.35 (brs, 1H, NH), 7.56 (t, 2H, *J* = 7.6, Ar-H), 8.08 (s, 1H, CH *_pyrazole_*); ^13^C NMR (101 MHz, CDCl_3_, ppm) δ 24.6, 25,7, 28.5, 35.3, 39.7, 39.7, 45.1, 51.8, 120.1, 120.9, 123.7, 128.9, 138.3, 139.4, 151.6, 163.4, 164.7, 165.2, 193.1 (CO). Anal. Calc. for C_23_H_27_N_7_O (417.52) C, 66.17; H, 6.52; N, 23.48. Found C, 66.32; H, 6.66; N, 23.21. HRMS-ESI (*m*/*z*) calculated for [M + H]^+^ 418.44; found:418.4225.

6. 1-(4-((4-Chlorophenyl)amino)-6-morpholino-1,3,5-triazin-2-yl)-6,6-dimethyl-1,5,6,7-tetrahydro-4*H*-indazol-4-one, **7f**

Off-white solid in 81% yield, mp 237–239 °C. ^1^H NMR (400 MHz, CDCl_3_, ppm) δ 1.11 (s, 6H, 2CH_3_), 2.41 (s, 2H, CH_2_), 3.21 (brs, 2H, CH_2_), 3.79 (t, 4H, *J* = 4.4 Hz, 2CH_2,_ CH_2_-N-CH_2_), 3.91 (brs, 4H, 2CH_2_; CH_2_-O-CH_2_), 7.31 (d, 2H, *J* = 8.8, Ar-H), 7.52 (d, 2H, *J* = 8.8 Hz, Ar-H), 8.12 (s, 1H, CH, pyrazole); ^13^C NMR (101 MHz, CDCl_3_, ppm) δ 28.5, 35.3, 39.7, 44.2, 51.7, 66.4, 121.4, 128.9, 140.1, 151.2, 162.7, 164.3, 176.0, 192.8 (**C**O). Anal. Calc. for C_22_H_24_ClN_7_O_2_ (453.93) C, 58.21; H, 5.33; N, 21.60. Found C, 58.43; H, 5.45; N, 21.83. HRMS-ESI (*m*/*z*) calculated for [M + H]^+^ 454.93; found:454.9522.

7. 1-(4-((4-Chlorophenyl)amino)-6-(piperidin-1-yl)-1,3,5-triazin-2-yl)-6,6-dimethyl-1,5,6,7-tetrahydro-4*H*-indazol-4-one, **7g**

Off-white solid in 85% yield, mp 258–260 °C. 1H NMR (400 MHz, CDCl_3_, ppm) δ 1.09 (s, 6H, 2CH_3_), 1.72 (brs, 6H, 3CH_2_), 2.39 (s, 2H, CH_2_), 3.19 (brs, 2H, CH_2_), 3.87 (brs, 4H, 2CH_2;_ CH_2_-N-CH_2_), 7.24 (d, 2H, *J* = 8.4, Ar-H), 7.55 (d, 2H, *J* = 8.0, Ar-H), 8.12 (s, 1H, CH_,_ *_pyrazole_*), 9.72 (brs, 1H, NH); ^13^C NMR (101 MHz, CDCl_3_, ppm) δ: 24.3, 25.7, 28.5, 35.3, 39.6, 45.6, 51.7, 121.5, 128.9, 140.1, 151.6, 163.4, 164.7, 165.2, 176.0, 193.1 (**C**O). Anal. Calc. for C_23_H_26_ClN_7_O (451.96) C, 61.12; H, 5.80; N, 21.69. Found C, 61.29; H, 5.91; N, 21.43. HRMS-ESI (*m*/*z*) calculated for [M + H]^+^ 452.96; found:452.8556.

8. 1-(4-((4-Methoxyphenyl)amino)-6-morpholino-1,3,5-triazin-2-yl)-6,6-dimethyl-1,5,6,7-tetrahydro-4*H*-indazol-4-one, **7h**

Beige crystals for ethyl acetate in 81% yield, mp 190–192 °C; ^1^H NMR (400 MHz, CDCl_3_, ppm) δ 1.08 (s, 6H, 2CH_3_), 2.35 (brs, 2H, CH_2_), 3.22 (brs, 2H, CH_2_), 3.75 (m, 7H, OCH*_3_*, 2CH_2,_ CH_2_-N-CH_2_), 3.84 (brs, 4H, 2CH_2_; CH_2_-O-CH_2_), 6.84 (d, 2H, *J* = 8.8, Ar-H), 7.38 (brs, 2H, Ar-H), 7.85 (brs, 1H, NH), 8.01 (s, 1H, CH *_pyrazole_*); ^13^C NMR (101 MHz, CDCl_3_, ppm) δ 28.5, 35.3, 39.5, 44.2, 51.7, 55.5, 66.4, 113.9, 121.2, 122.1, 130.6, 139.6, 151.6, 156.3, 161.7, 163.3, 164.8, 176.0, 192.8 (CO). Anal. Calc. for C_23_H_27_N_7_O_3_ (449.52) C, 61.46; H, 6.05; N, 21.81. Found C, 61.67; H, 6.21; N, 22.01. HRMS-ESI (*m*/*z*) calculated for [M + H]^+^ 450.52; found: 450.2253.

9. 1-(4-((4-Methoxyphenyl)amino)-6-(piperidin-1-yl)-1,3,5-triazin-2-yl)-6,6-dimethyl-1,5,6,7-tetrahydro-4*H*-indazol-4-one, **7i**

Beige crystals from ethyl acetate in 85% yield, mp 178–180 °C; 1H NMR (400 MHz, CDCl_3_, ppm) δ: 2.09 (brs, 2H, CH_2_), 2.44 (brs, 2H, CH_2_), 3.35 (brs, 2H, CH_2_), 3.66 (brs, 8H, 4OCH_2_-), 3.78 (brs, 8H, 4NCH_2_-), 8.02 (s, 1H, CH*_pyrazole_*); ^13^C NMR (101 MHz, CDCl_3_, ppm) δ 25.7, 28.5, 35.2, 39.6, 45.2, 51.7, 55.4, 114.0, 121.1, 122.0, 130.6, 139.5, 151.9, 156.0, 163.3, 169.4, 175.2, 193.1 (**C**O). Anal. Calc. for C_24_H_29_N_7_O_2_ (447.24) C, 64.41; H, 6.53; N, 21.91. Found C, 64.23; H, 6.41; N, 22.013. HRMS-ESI (*m*/*z*) calculated for [M + H]^+^ 447.24; found: 448.2453.

10. 1-(4,6-Dimorpholino-1,3,5-triazin-2-yl)-1,5,6,7-tetrahydro-4*H*-indazol-4-one, **7j**

White precipitate from ethyl acetate in 94% yield; mp 260–262 °C; ^1^H NMR (400 MHz, DMSO-*d_6_*) δ 2.07 (brs, 2H, CH_2_), 2.42 (brs, 2H, CH_2_), 3.37 (brs, 2H, CH_2_), 3.64 (brs, 8H, 4OCH_2_-), 3.76 (brs, 8H, 4NCH_2_-), 7.98 (s, 1H, CH*_pyrazole_*); ^13^C NMR (101 MHz, DMSO-*d_6_*) δ 23.1, 25.0, 37.8, 43.5, 43.6, 66.8, 66.9, 121.2, 138.7, 152.8, 162.4, 164.6, 193.0; Anal. Calc. for C_18_H_23_N_7_O_3_ (385.43): C, 56.09; H, 6.02; N, 25.44. Found: C, 56.35; H, 6.19; N, 25.66; HRMS-ESI (*m*/*z*) calculated for [M + H]^+^ 386.43; found: 386.4432.

11. 1-(4,6-Di(piperidin-1-yl)-1,3,5-triazin-2-yl)-1,5,6,7-tetrahydro-4*H*-indazol-4-one, **7k**

Off-white precipitate from ethyl acetate in 92% yield; mp 199–200 °C; ^1^H NMR (400 MHz, DMSO-*d_6_*) δ 1.51 (brs, 8H, 4CH_2_), 1.62 (brs, 4H, 2CH_2_), 2.05 (brs, 2H, CH_2_), 2.42 (brs, 2H, CH_2_), 3.28 (brs, 2H, CH_2_), 3.73 (s, 8H, 4NCH_2_-), 7.98 (s, 1H, CH*_pyrazole_*); ^13^C NMR (101 MHz, DMSO-*d_6_*) δ 23.1, 24.2, 24.9, 25.2, 37.3, 43.8, 121.0, 138.3, 152.4, 162.5, 164.3, 193.0; Anal. Calc. for C_20_H_27_N_7_O (381.48): C, 62.97; H, 7.13; N, 25.70. Found: C, 63.12; H, 7.30; N, 25.97; HRMS-ESI (*m*/*z*) calculated for [M + H]^+^ 382.48; found: 382.3455.

12. 1-(4-Morpholino-6-(piperidin-1-yl)-1,3,5-triazin-2-yl)-1,5,6,7-tetrahydro-4*H*-indazol-4-one, **7l**

Off-white precipitate from ethyl acetate in 89% yield; mp 212–214 °C; ^1^H NMR (400 MHz, DMSO-*d_6_*) δ 1.54 (brs, 4H, 2CH_2_), 1.64 (brs, 2H, CH_2_), 2.05-2.18 (m, 2H, CH_2_), 2.42-2.46 (m, 2H, CH_2_), 3.33 (brs, 2H, CH_2_), 3.65 (brs, 4H, 2OCH_2_), 3.76 (brs, 8H, 4NCH_2_), 8.02 (s, 1H, CH*_pyrazole_*); ^13^C NMR (101 MHz, DMSO-*d_6_*) δ 23.1, 24.1, 24.9, 25.2, 37.3, 44.3, 43.7, 65.9, 121.1, 138.5, 152.6, 162.5, 164.8, 193.0; Anal. Calc. for C_19_H_25_N_7_O_2_ (383.46): C, 59.51; H, 6.57; N, 25.57. Found: C, 59.73; H, 6.66; N, 25.80; HRMS-ESI (*m*/*z*) calculated for [M + H]^+^ 384.46; found: 384.4465.

13. 1-(4-Morpholino-6-(phenylamino)-1,3,5-triazin-2-yl)-1,5,6,7-tetrahydro-4*H*-indazol-4-one, **7m**

Off-white precipitate from ethyl acetate in 94% yield, mp 298–300 °C; ^1^H NMR (400 MHz, CDCl_3_) δ 2.26 (brs, 2H, CH_2_), 2.61 (brs, 2H, CH_2_), 3.36 (brs, 2H, CH_2_-), 3.89 (brs, 4H, 2OCH_2_), 3.97 (brs, 4H, 2 NCH_2_), 7.28 (m, 2H, Ar-H), 7.39 (d, *J* = 8.0 Hz, 2H, Ar-H), 7.47 (d, *J* = 8.1 Hz, 2H, Ar-H), 7.47 (brs, 1H, NH), 8.13 (s, 1H, CH *_pyrazole_*), 9.92 (s, 1H, NH); ^13^C NMR (101 MHz, CDCl_3_) δ 22.7, 24.7, 37.1, 45.4, 66.2, 113.6, 116.4, 122.3, 123.6, 129.6, 134.7, 137.6, 154.4, 160.2, 161.3, 194.9; Anal. Calc. for C_20_H_21_N_7_O_2_ (391.44): C, 61.37; H, 5.41; N, 25.05. Found: C, 61.62; H, 5.52; N, 25.37; HRMS-ESI (*m*/*z*) calculated for [M + H]^+^ 392.44; found: 392.3455.

14. 1-(4-(Phenylamino)-6-(piperidin-1-yl)-1,3,5-triazin-2-yl)-1,5,6,7-tetrahydro-4*H*-indazol-4-one, **7n**

White precipitate from ethyl acetate in 87% yield; mp 256–258 °C; ^1^H NMR (400 MHz, DMSO-*d_6_*) δ 1.161 (s, 4H, 2 CH_2_), 1.68 (s, 2H, CH_2_), 2.10 (brs, 2H, CH_2_), 2.49 (brs, 2H, CH_2_), 3.33 (brs, 2H, CH_2_), 3.82 (s, 4H, 2NCH_2_), 7.05 (m, 1H, Ar-H), 7.32 (t, *J* = 8.4 Hz, 2H, Ar-H), 7.80 (d, *J* = 8.8 Hz, 2H, Ar-H), 8.08 (s, 1H, CH*_pyrazole_*), 10.05 (brs, 1H, NH); ^13^C NMR (101 MHz, DMSO-*d_6_*) δ 23.1, 24.1, 24.9, 25.4, 37.4, 44.3, 120.3, 121.2, 122.7, 128.6, 138.6, 139.3, 152.7, 162.5, 164.2, 193.0; Anal. Calc. for C_21_H_23_N_7_O (389.46): C, 64.76; H, 5.41; N, 25.05. Found: C, 61.62; H, 5.95; N, 25.18; HRMS-ESI (*m*/*z*) calculated for [M + H]^+^ 390.46; found: 390.3545.

15. 1-(4-((4-Chlorophenyl)amino)-6-morpholino-1,3,5-triazin-2-yl)-1,5,6,7-tetrahydro-4*H*-indazol-4-one, **7o**

Off-white precipitate from ethyl acetate in 90% yield; mp 292–294 °C; ^1^H NMR (400 MHz, DMSO-*d_6_*) δ 2.01 (brs, 2H, CH_2_), 2.43 (brs, 2H, CH_2_), 3.35 (brs, 2H, CH_2_), 3.70 (brs, 4H, 2OCH_2_), 3.79 (brs, 4H, 2NCH_2_-), 7.38 (d, *J* = 8.8 Hz, 2H, Ar-H), 7.77 (d, 2H, *J* = 8.8 Hz, Ar-H), 8.05 (s, 1H, CH*_pyrazole_*), 10.23 (brs, 1H, NH); ^13^C NMR (101 MHz, DMSO-*d_6_*) δ 23.1, 25.0, 37.4, 43.8, 65.9, 111.9, 121.3, 121.8, 128.5, 138.2, 140.4, 152.9, 163.2, 164.5, 193.1; Anal. Calc. for C_20_H_20_ClN_7_O_2_ (425.88): C, 56.41; H, 4.73; N, 23.02. Found: C, 56.61; H, 4.87; Cl, 8.51; N, 23.20; HRMS-ESI (*m*/*z*) calculated for [M + H]^+^ 426.88; found: 427.1144.

16. 1-(4-((4-Bromophenyl)amino)-6-morpholino-1,3,5-triazin-2-yl)-1,5,6,7-tetrahydro-4*H*-indazol-4-one, **7p**

Off-white precipitate from ethyl acetate in 95% yield; mp 290–292 °C; ^1^H NMR (400 MHz, CDCl_3_) δ 2.26 (t, *J* = 6.2 Hz, 2H, CH_2_), 2.63 (t, *J* = 6.6 Hz, 2H, CH_2_), 3.38 (t, *J* = 6.4 Hz, 2H, CH_2_), 3.88 (m, 4H, 2OCH_2_-), 3.97 (m, 4H, 2NCH_2_), 7.32 (d, *J* = 8.7 Hz, 2H, Ar-H), 7.51 (d, *J* = 8.3 Hz, 2H, Ar-H), 8.10 (s, 1H, CH*_pyrazole_*), 9.95 (s, 1H, NH); ^13^C NMR (101 MHz, CDCl_3_) δ 22.5, 24.6, 37.0, 45.5, 66.0, 110.6, 113.5, 116.3, 119.2, 120.1, 123.5, 124.2, 132.4, 133.6, 142.2, 153.2, 154.8, 160.5, 161.0, 195.8; Anal. Calc. for C_20_H_20_BrN_7_O_2_ (470.33): C, 51.07; H, 4.29; N, 20.85, Found: C, 51.37; H, 4.41; N, 20.99; HRMS-ESI (*m*/*z*) calculated for [M + H]^+^ 471.33; found: 471.3255.

17. 1-(4-((4-Methoxyphenyl)amino)-6-morpholino-1,3,5-triazin-2-yl)-1,5,6,7-tetrahydro-4*H*-indazol-4-one, **7q**

Off-white precipitate from ethyl acetate in 88% yield; mp 254–256 °C; ^1^H NMR (400 MHz, DMSO-*d_6_*) δ 2.04 (brs, 2H, CH_2_), 2.44 (brs, 2H, CH_2_), 3.35 (brs, 2H, CH_2_), 3.69 (brs, 4H, 2OCH_2_), 3.74 (brs, 4H, 2NCH_2_), 3.79 (s, 3H, OCH_3_), 6.91 (d, *J* = 8.8 Hz, 2H, Ar-H), 7.62 (d, *J* = 8.6 Hz, 2H, Ar-H), 8.04 (s, 1H, CH*_pyrazole_*), 10.00 (s, 1H, NH); ^13^C NMR (101 MHz, DMSO-*d_6_*) δ 23.1, 24.9, 37.8, 44.4, 55.2, 65.9, 113.9, 121.2, 121.7, 124.2, 138.5, 138.6, 152.8, 154.6, 164.9, 193.0; Anal. Calc. for C_21_H_23_N_7_O_3_ (421.46): C, 59.85; H, 5.50; N, 23.26. Found: C, 59.98; H, 5.66; N, 23.86; HRMS-ESI (*m*/*z*) calculated for [M + H]^+^ 422.46; found: 422.4566.

18. 1-(4-((4-Chlorophenyl)amino)-6-(piperidin-1-yl)-1,3,5-triazin-2-yl)-1,5,6,7-tetrahydro-4*H*-indazol-4-one, **7r**

White precipitate from ethyl acetate in 91% yield; mp. 265–267 °C; ^1^H NMR (400 MHz, CDCl_3_) δ 1.75–1.78 (d, *J* = 11.0 Hz, 6H, 3 CH_2_), 2.30 (d, *J* = 6.6 Hz, 2H, CH_2_), 2.63 (s, 2H, CH_2_), 3.37 (s, 2H, CH_2_), 3.88 (s, 4H, 2NCH_2_-), 7.38 (s, 2H, Ar-H), 7.48 (s, 2H, Ar-H), 8.13 (s, 1H, CH *_pyrazole_*), 10.21 (s, 1H, NH); ^13^C NMR (101 MHz, CDCl_3_) δ 22.86, 24.17, 25.29, 27.7, 37.30, 47.84, 111.52, 116.41, 124.39, 128.81, 133.79, 142.42, 154.16, 161.58, 162.63, 194.68; Anal. Calc. for C_21_H_22_ClN_7_O (423.91): C, 59.50; H, 5.23; Cl, 8.36; N, 23.13, O, 3.77. Found: C, 59.66; H, 5.39; Cl, 8.57; N, 23.41, O, 3.99. (*m*/*z*) Calcd: 423.91; LC-MS [M + H]^+^ Found: 425.0025.

19. 1-(4-((4-Bromophenyl)amino)-6-(piperidin-1-yl)-1,3,5-triazin-2-yl)-1,5,6,7-tetrahydro-4*H*-indazol-4-one, **7s**

Off-white precipitate from ethyl acetate in 96% yield; mp. 280–283 °C; ^1^H NMR (400 MHz, CDCl_3_) δ 1.73 (td, *J* = 10.1, 9.6, 4.5 Hz, 6H, 3 CH_2_), 2.24 (s, 2H, CH_2_), 2.57 (s, 2H, CH_2_), 3.36 (s, 2H, CH_2_), 3.88 (s, 4H, 2NCH_2_), 7.47 (s, 4H, Ar-H), 8.13 (s, 1H, CH*_pyrazole_*), 9.95 (s, 1H, -NH); ^13^C NMR (101 MHz, DMSO-*d_6_*) δ 22.77, 24.61, 25.45, 27.41, 36.16, 43.88, 111.49, 117.25, 120.79, 122.24, 124.34, 130.17, 138.83, 148.84, 154.24, 161.45, 162.24, 165.31, 193.05; Anal. Calc. for C_21_H_22_BrN_7_O (468.36): C, 53.85; H, 4.73; Br, 17.06, N, 20.93, O, 3.42, Found: C, 54.12; H, 4.89; Br, 17.39; N, 21.20, O, 3.55, (*m*/*z*) Calcd: 468.36; LC-MS [M + H]^+^ Found: 469.1269.

20. 1-(4-((4-Methoxyphenyl)amino)-6-(piperidin-1-yl)-1,3,5-triazin-2-yl)-1,5,6,7-tetrahydro-4*H*-indazol-4-one, **7t**

Off-white precipitate from ethyl acetate in 82% yield; mp. 233–235 °C; ^1^H NMR (400 MHz, DMSO-*d_6_*) δ 1.47 (s, 4H, 2 CH_2_), 1.66 (s, 2H, CH_2_), 2.09 (s, 2H, CH_2_), 2.34 (s, 2H, CH_2_), 3.05 (2H, CH_2_), 3.74 (s, 4H, 2NCH_2_-), 3.81 (s, 3H, -OCH_3_), 6.90 (s, 2H, Ar-H), 7.61 (s, 2H, Ar-H), 8.04 (s, 1H, CH*_pyrazole_*), 10.06 (s, 1H, NH); ^13^C (101 MHz, DMSO-*d_6_*) δ 20.80, 24.66, 25.82, 26.55, 37.26, 43.83, 55.72, 112.27, 120.79, 122.09, 123.00, 125.76, 133.16, 137.58, 153.21, 155.94, 163.01, 164.45, 166.42, 193.57, Anal. Calc. for C_22_H_25_N_7_O_2_ (419.49): C, 62.99; H, 6.01; N, 23.37, O, 7.63. Found: C, 63.15; H, 6.17; N, 23.52, O, 7.85. (*m*/*z*) Calcd: 419.49; LC-MS [M + H]^+^ Found: 420.2336.

### 2.2. Biology

#### 2.2.1. Cell Culture

The parental MCF-7 (breast cancer), MDA-MB-231 (triple-negative breast cancer), U-87 MG (glioblastoma), A549 (non-small cell lung cancer), and PANC-1 (pancreatic cancer) cell lines and HDFs (human dermal fibroblasts) were obtained from the American Type Culture Collection (ATCC, Manassas, VA, USA). MCF-7 and A549 cells were cultured as an attached monolayer and maintained in RPMI 1640 medium (EuroClone, Milan, Italy) supplemented with 10% (*v*/*v*) heat-inactivated fetal bovine serum (FBS) (EuroClone, Milan, Italy), 1% penicillin-streptomycin (EuroClone, Milan, Italy), and 2 mM L-glutamine. MDA-MB-231 cells were cultured as an attached monolayer and maintained in MEM (EuroClone, Boston, MA, USA) supplemented with 10% (*v*/*v*) heat-inactivated fetal bovine serum (FBS) (EuroClone, Milan, Italy), 1% penicillin-streptomycin (EuroClone, Milan, Italy), and 2 mM L-glutamine. U-87, PANC-1 and HDFs were cultured as an attached monolayer and maintained in DMEM (EuroClone, Milan, Italy) supplemented with 10% (*v*/*v*) heat-inactivated FBS (EuroClone, Milan, Italy), 1% penicillin-streptomycin (EuroClone, Milan, Italy), and 2 mM L-glutamine. All cells were incubated at 37 °C in a 5% CO_2_ tissue culture incubator (Memmert, Schwabach, Germany).

#### 2.2.2. Cell Viability Assay (MTT)

To determine the IC_50_ of the synthesized compounds **5a**–**i** and **7a**–**t** on the cell lines, an MTT assay was performed [51]. MDA-MB-231, MCF-7, and PANC-1 cells were seeded into 96-well plates at 8 × 10^3^ cells/well (Corning, New York, NY, USA), and U-87 and A549 cells and HDFs were seeded at 6.5 × 10^3^ cells/well. All cell lines were treated with concentrations of the tested compounds ranging from 0.5 to 500 μg/mL. Cells were then incubated at 37 °C in a 5% CO_2_ incubator for 72 h, after which the old media was aspirated and the MTT assay salt (Bioworld, Visalia, CA, USA) in 100 μL of fresh media was added to each well. Next, plates were incubated at 37 °C for 3 h, then 50 μL of solubilization solution (DMSO) was added to each well to determine cell viability. The absorbance of the solution was measured at 560 nm using a Glomax plate reader (Promega, Madison, WI, USA).

#### 2.2.3. EGFR Protein Kinase (PK) Inhibition

The EGFR-TK assay kit (ADP-Glo^TM^ kinase assay, Cat No. V9261, Promega, USA) was used to determine the inhibitory capacity of compounds **7d**, **7f** and **7c** against EGFR. The autophosphorylation percentage inhibition by the compounds was calculated using the following equation: $100-\left[\frac{Control}{Treated}-Control\right]$ using the curves of percentage inhibition of 8 concentrations of each compound. IC_50_ values were calculated using GraphPad Prism 7 software (Dotmatics, San Diego, CA, USA) [52].

#### 2.2.4. PI3K/AKT/mTOR Downstream Signaling Pathway

ELISA kits of PI3K assay kit (Cat. No. MBS268899, Promega, Madison, MI, USA), AKT assay kit (Cat. No. MBS9511022, Promega, USA), and mTOR assay kit (Cat. No. LS-F21147, Promega, USA) were used to study the PI3k/AKT/mTOR downstream signaling pathway in MDA-MB-231 cells treated with **7d**, **7f** and **7c** at their IC_50_ values or in untreated cells of the same line.

#### 2.2.5. Apoptosis by Flow Cytometry

To study the growth inhibition of all the cell lines treated with **5a**–**i** and **7a**–**t**, the mechanism of apoptosis was determined by Annexin V/Propidium iodide (PI) stain using flow cytometry. Each cell line was seeded at 1 × 10^5^ cells/well in 6-well plates and exposed to the IC_50_ concentration of each compound for that specific cell line, as shown in Table 1. After 72 h, the cells were trypsinized using StemPro™ Accutase™ Cell Dissociation Reagent (Gibco™, Inchinnan, UK). The collected cells were then washed with PBS. Next, the Annexin V/PI apoptosis kit (Invitrogen, Waltham, MA, USA) was used to stain the cell pellets, following the manufacturer’s instructions. 10,000 events were counted by BD FACS CANTO II and analyzed using BD FACS Diva™ software version 7.0.

#### 2.2.6. Gene Expression Analysis Using RT-qPCR

To further examine the apoptotic pathway, we assessed the gene expression of P53, Bax, Caspases-3, -8, and -9 as proapoptotic genes, Bcl-2 as the anti-apoptotic gene, and the downstream pathway of PI3K/AKT/mTOR. MDA-MB-231 cells were treated with compound **7f** at its IC_50_ value for 48 h. After completing the treatment, cells were collected, and total RNA was extracted using the RNeasy^®^ Mini Kit (Qiagen, Hilden, Germany). cDNA was then synthesized using 500 ng of RNA (*i*-Script cDNA synthesis kit, BioRad, Hercules, CA, USA). Finally, each RT-qPCR reaction was performed following routine work [53]. The Ct values were then collected to calculate the relative gene expression in all samples by normalization to the β-actin housekeeping gene [54,55].

#### 2.2.7. CDOCKER Docking

CDOCKER is a CHARMm-based simulated annealing/molecular dynamics method that uses rigid receptors for docking [56]. The CDOCKER protocol includes the following steps: (i) A set of ligand conformations is generated using high-temperature molecular dynamics starting with different random seeds. (ii) Random orientations of the conformations are produced by translating the center of the ligand to a specified location within the receptor active site and performing a series of random rotations. (iii) A softened energy is calculated, and the orientation is kept if the energy is less than a specified threshold. This process continues until either the desired number of low-energy orientations is found, or the maximum number of poor orientations has been attempted. (iv) Each orientation is subjected to simulated annealing molecular dynamics. The temperature is heated to a high preset temperature and then cooled to the target temperature. (v) A final minimization of the ligand in the rigid receptor using non-softened potential is performed. For each final pose, the CHARMm energy (interaction energy plus ligand strain) and the interaction energy alone is calculated. The poses are sorted by CHARMm energy and the top-scoring (most negative, thus favorable to binding) poses are retained. To enhance performance and shorten calculation times, a non-bond energy grid is used to calculate interaction energy rather than the full potential energy terms commonly used by CHARMm.

The following CDOCKER parameters were implemented in this study. A binding site sphere of 10.14 Å radius surrounding the copied co-crystallized ligand from the EGFR structure (PDB code: 6v6o) was implemented. The conformers of the starting ligands were energy-minimized and then heated to 1000 K over 1000 molecular dynamics steps to generate 50 starting random conformations for each ligand. Each random conformer was rotated 50 times within the binding pocket for subsequent energy refinement. The van der Waals energies of the resulting conformers/poses were examined and those of ≥300 kcal/mol were discarded. Surviving conformers/poses were subjected to a cycle of simulated annealing over 2000 heating steps to the targeted temperature of 700 K, followed by 5000 cooling steps to the targeted temperature of 300 K. The docked poses were energy-minimized to a gradient tolerance of zero kcal/mol/Å. A total of 599 poses were saved for subsequent scoring.

#### 2.2.8. Scoring of Docked Poses

The highest-ranking docked conformers/poses generated by CDOCKER were scored using 9 scoring function: Jain [57,58], LigScore1, LigScore2 [59], PLP1, PLP2 [57], PMF, PMF04 [60,61], -CDOCKER Energy, and -CDOCKER Interaction Energy [56].

LigScore1 and LigScore2 scores were calculated using the CFF force field (version 1.02) and grid-based energies with a grid extension of 7.5 Å across the binding site. PMF scores were calculated using cutoff distances of 12.0 Å for carbon–carbon interactions and other atomic interactions, while PMF04 scores were calculated employing cutoff values of 6.0 and 9.0 Å for carbon-carbon interactions and other atomic interactions, respectively. -CDOCKER Energy and -CDOCKER Interaction Energy were calculated using the Momany-Rone ligand partial charge method. Docked conformers/poses were selected based on consensus among the 9 scoring functions [62,63]. The consensus function assigned a value of 1 for any molecular pose ranked within the highest 20% by the particular scoring function; otherwise, it was assigned a zero value (i.e., fit was within the lowest 80%). Subsequently, the consensus function summed up the scores for each molecular pose/conformer and ranked the molecular orientation. Docked poses of a particular ligand that achieved consensus among at least 8 scoring functions were selected and saved.

## 3. Results and Discussion

### 3.1. Chemistry

Hydrazino *s*-triazine derivatives **3a**–**l** required for this study were prepared following our reported method [32,33,50] (Scheme 1), where cyanuric chloride **1** was reacted with the first nucleophile at 0 °C in the presence of NaHCO_3_ as a base and acetone–water as a solvent for 2 h. The second nucleophile was added at the same temperature in the presence of 1 equiv. of NaHCO_3_ and the reaction was stirred at room temperature (rt) for 24 h. The products 2-chloro-4,6-disubstituted *s*-triazine derivatives **2a**–**l** were treated with hydrazine hydrate in ethanol under reflux for 6–8 h to afford the hydrazino derivatives **3a**–**l** [32,33,50], which were used directly in the next step without further purification.

To optimize the reaction conditions for the incorporation of the pyrazole ring into the *s*-triazine scaffold, we started with the study of 4-hydrazinyl-6-morpholino-*N*-phenyl-1,3,5-triazin-2-amine **3d** as the model substrate (Table 1). Initially, ethylacetocacetate 4 (1 equiv.) was reacted with neat DMF-DMA (1.2 equiv.) at rt for 5–10 min to generate the enaminedinone intermediate I (Scheme 2), followed by the addition of 4-hydrazinyl-6-morpholino-*N*-phenyl-1,3,5-triazin-2-amine **3d** (1 equiv.) in 5% acetic acid in ethanol. The reaction mixture was refluxed for 4 h and monitored by TLC (*n*-hexane-ethylacetate, 1:1), which showed two products. The reaction did not promote at all with longer time (8 h) (Table 1, entries 1 and 2). The ^1^H-NMR spectrum (Supporting information, Figure S1) showed the two products **5d** and **5d’** in a 1:1 ratio. Given that the yield of these products (**5d** and **5d’**, Scheme 2) was low, we hypothesized that this parameter could be affected by reaction time or acid loading in the medium.

Encouraged by this result, we focused on the solvent effect (Table 1). The reaction was repeated by altering the AcOH ratio (10%, 20% and 33.3%). Screening of AcOH loading revealed that the increasing percentage of AcOH was crucial, as the chemical yield of the cyclized product rather than the open form was enhanced. The optimal reaction condition was 33.3% AcOH, affording the final cyclized compound **5d** in 90% yield in 6 h (Entry 5), which increased to 95% after 8 h (Entry 6).

**Table 1:** Optimization of the cycloaddition reaction for the formation of the pyrazole derivatives.

Entry	Solvent	Time (h)	5d’ %	5d%
1	5% AcOH-EtOH	4	50	50
2	5% AcOH-EtOH	8	50	50
3	10% AcOH-EtOH	8	40	60
4	20% AcOH-EtOH	6	~20	~80
5	AcOH-EtOH (1:2)	6	traces	~90
6	AcOH-EtOH (1:2)	8	traces	~95

The tentative synthetic reaction pathway is shown in Scheme 2. It comprises the initial formation of the enamine intermediate **I** in situ, followed by nucleophilic attack by the hydrazine derivative in the presence of AcOH to afford the final cyclized product. The hydrazine derivative **3d** could possible attack the enamine intermediate **I** *via* two pathways (**A** or **B**). The enamine intermediate **I** is typically more reactive in bath **A** (**5d**) *via* initial addition-elimination amine-exchange of the dimethylamino group by the hydrazine derivative to afford the product **5d** through the open analogue **5d’** after removal of water molecule form the intermediate **II** [64,65]. This analogue of pyrazole derivative **5d** is more favor than its analogue in path **B** which first formation of the Schiff base (intermediate **III**) then Nu-attack to the enamine to afford the cyclized analogue **5d’’** after removal of NEMe_2_ (Scheme 3) [64,65]. 

Under the optimized conditions and configured the synthetic pathway, various hydrazine derivatives **3a**–**i**, including various heterocycles (morpholine and piperidine) and, substituted aryls employed in this reaction and furnished the products **5a**–**i**, as indicated in Scheme 4 in excellent yield. The spectral data for the synthesized compounds **5a**–**i** are provided in the supporting information, see Figures S2–S9).

To explore a cyclized 1,3-dicarbonyl compounds instead of ethylacetoacetate, for example, 5,5-dimethyl-1,3-cyclohexandione **6a** or 1,3-cyclohexadionone **6b** were employed in the reaction under the optimized reaction condition used for the synthesis of **5a**–**i** but did not afford the final compound **7**. In contrast, when the reaction was run in a neat AcOH, complete reaction occurred after 8–12 h, as shown by TLC (*n*-hexane-ethylacetate, 1:1). After completion of the reaction, the acidic solution was poured into ice-cold water and extracted with AcOEt or CHCl_3_. Next, the organic phase was washed with 10% Na_2_CO_3_ solution and NaCl solution and then dried over anhydrous MgSO_4_. The desired products **7a**–**t** (Scheme 5) were obtained after evaporation of the solvent.

The structures of all the products obtained were established by IR, NMR (^1^H and ^13^C), elemental analysis, and HRMS-ESI (Figures S10–S29 for the NMR spectrum and Figures S30–S38 for the HRMS). In addition, compound **7t** (CCDC No.: 2177427) was assigned based on single crystal X-ray diffraction analysis [66,67,68,69] (see Supporting Information).

### 3.2. Biology

#### 3.2.1. Cytotoxicity

**Figure 2:**
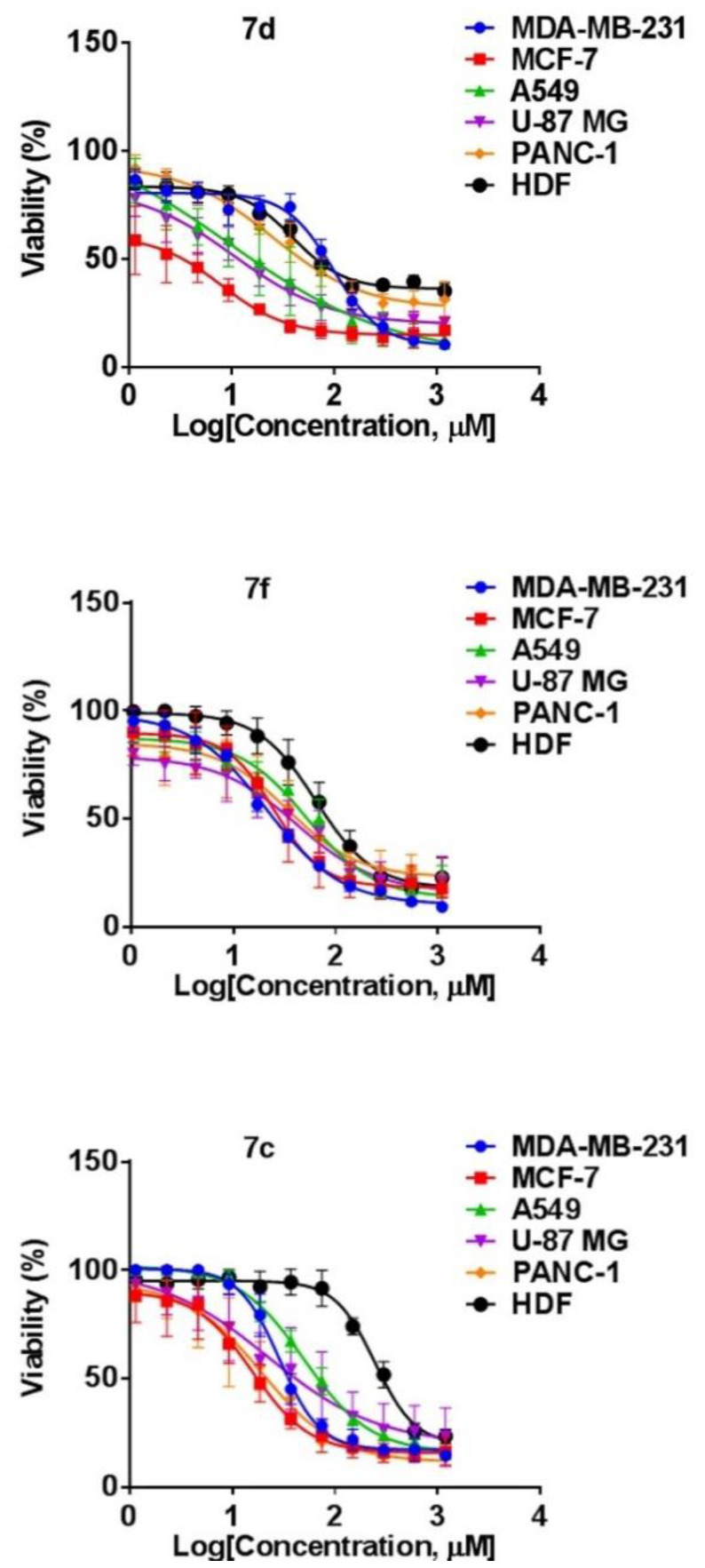
Representative cell viability results used to calculate the IC_50_ values for compounds **7d**, **7f**, and **7c**.

The anticancer activity of the pyrazolyl-*s*-triazine derivatives (**5a**–**i** and **7a**–**t**; Table 2) was studied in the following cell lines: human breast cancer (MCF-7 and MDA-MB-231); glioblastoma (U-87 MG); non-small cell lung cancer (A549); pancreatic cancer (PANC-1); and human dermal fibroblasts (HDFs). Most of the derivatives affected the viability of the six cancer cell lines, as determined by the MTT cell viability assay [51] (Table 2 and Figure 2). The most effective compounds were **7d**, **7f**, and **7c**. Of note, the substitution of the triazine ring had a marked impact on anticancer activity (Table 2). In most cases, compounds **5a**–**i** (carboxylate derivatives) showed lower activity than **7a**–**t** (5,5-dimethyl-1,3-cyclohexadione and 1,3-cyclohexadionone derivatives). Moreover, 5,5-dimethyl-1,3-cyclohexadione *s*-triazine derivatives **7a**–**i** showed higher activity against most cancer cells compared with 1,3-cyclohexadionone derivatives **7j**–**t**. In general, compounds with the aniline moiety exerted greater activity compared to those with a morpholine or piperidine ring. Interestingly, piperidine and morpholine can be considered to belong to the same family in some aspects of their synthetic chemistry. The presence of the piperidine moiety was detrimental for anticancer activity, as observed in compounds with both the piperidine and aniline ring attached to the triazine ring, compared to morpholine analogs. Compounds with two aniline moieties showed the highest activity (Table 2). Thus, **5d**, which contains morpholine and aniline, showed IC_50_ values of 39.4 ± 1.9, 42.2 ± 3.4, 73.8 ± 21.0, 26.4 ± 2.7, 10.5 ± 2.4, and 33.4 ± 3.7 (µM) against MDA-MB-231, U-87 MG, PANC-1, A549, MCF-7 and HDFs, respectively. In contrast, compounds with two morpholine **5a**, two piperidine **5b**, one morpholine and one piperidine **5c** and one piperidine and aniline **5e** showed lower activity, with higher IC_50_ values. In addition, compounds with substituted aniline (*p*-bromo-, *p*-chloro-, or *p*-methoxyanilne) exerted lower activity than unsubstituted aniline in this series of compounds. The same behavior was observed with series of compounds **7a**–**t** (Table 2).

In general, the IC_50_ of **7d**, **7f**, and **7c** revealed a reduction in the viability of the cancer cell lines tested compared to the normal fibroblasts. Interestingly, there were some variations in the IC_50_ values of the compounds in the distinct cancer cell lines. For instance, **7c** showed similar IC_50_ values against MCF-7, U-87 MG, and PANC-1 cell lines (15.7–17.1 µM) while the values against the MDA-MB-231 and A549 cells were 28.2 and 49.8 µM, respectively. Compound **7d** was found to be more powerful against MCF-7, U-87 MG, and A549 cells, with IC_50_ values of 8.3, 10.9, and 12.4 µM, respectively. On the other hand, the MDA-MB-231 cancer cell line showed resistance to **7d** compared to the HDFs. Compound **7f** was the least effective against the cancer cell lines. However, it showed selectivity against the MDA-MB-231 cell line compared to the other compounds and the IC_50_ of other cell lines (Table 2, Figure 2).

#### 3.2.2. EGFR Enzymatic Assay

The capacity of **7d**, **7f**, and **7c** to inhibit EGFR was tested (Table 3). Compounds **7d** and **7f** exhibited potent EGFR inhibitory activity, with IC_50_ values of 59.24 and 70.3 nM, compared to Tamoxifen, with an IC_50_ value of 69.1 nM. Compound **7c** showed moderate activity (IC_50_ value of 81.6 nM).

#### 3.2.3. PI3K/AKT/mTOR Downstream Signaling Pathway

To study the molecular target for the promising cytotoxicity of **7d**, **7f**, and **7c**, which showed the highest cytotoxic activity and promising EGFR inhibitory capacity, these compounds were tested against the PI3K, AKT, and mTOR downstream inhibition pathway. The PI3K/AKT/mTOR signaling cascade is important in many cellular processes, including growth and proliferation, apoptosis, survival, and metabolism, all of which contribute to tumor progression [70,71].

These compounds showed promising capacity to inhibit PI3K/AKT/mTOR (Table 4). In this regard, **7d** and **7f** exhibited remarkable PI3K/AKT/mTOR inhibitory activity by 0.66/0.82/0.8 and 0.35/0.56/0.66-fold, respectively, by inhibiting their concentrations to 4.39, 37.3, and 69.3 ng/mL in the **7d**-treatment, and to 2.39, 25.34, and 57.6 ng/mL in the **7f**-treatment compared to the untreated control; while compound **7c** did not show inhibitory activity compared to the control.

#### 3.2.4. Apoptosis by Flow Cytometry

**Figure 3:**
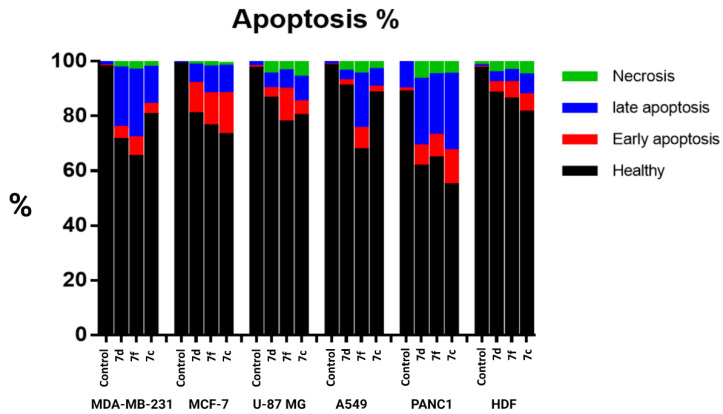
A representative diagram showing the percentage of apoptosis induction by **7d**, **7f**, and **7c** in MDA-MB-231, MCF-7, U87 MG, A459, and PANC-1 cancer cell lines compared to HDFs. The results showed a higher percentage of apoptosis induced in all cancer cell lines compared to HDFs.

**Figure 4:**
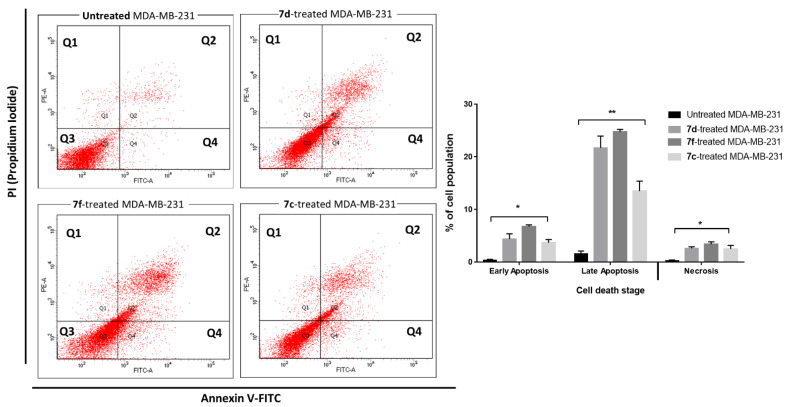
Annexin V/PI staining for apoptosis–necrosis assessment. Q1: Necrosis, Q2: Late apoptosis, Q3: Healthy cells, Q4: Early apoptosis in untreated and treated MDA-MB-231 cells with bar representation. * (*p* ≤ 0.05) and ** (*p* ≤ 0.001) significantly different using the unpaired test in GraphPad Prism.

The results of the apoptosis assay showed significant induction of apoptosis by **7d**, **7f**, and **7c** in all the cancer cell lines compared to the normal cell line. In particular, the greatest induction of apoptosis by these three compounds occurred in the MDA-MB-231 and PANC1 cancer cell lines. In this regard, they induced total apoptosis in MDA-MB-231 cells by 26.1%, 31.54%, and 17.2%, respectively, compared to 1.43% in the untreated control (Figure 3 and Figure 4). Additionally, they induced total apoptosis in PANC1 cells by 31.7%, 30.4%, and 40.3%, respectively, compared to 11% in the untreated control (Figure 3 and Figure S39). Furthermore, **7f** showed more specific activation of apoptosis in the A549 cancer cell line (27.7% compared to 0.11% in control) compared to the other two compounds (Figure 3 and Figure S39), and it induced total apoptosis in MCF-7 cells (21.53% compared to 0.67% in control). Histograms for Annexin V/PI stainting for the tested compounds in cancer cells were supported in the Supporting Information Figure S39. Results elucidated that cytotoxic activities in cancer cells were due to apoptosis rather than necrosis.

#### 3.2.5. Compound **7f** Upregulated Pro-Apoptotic Genes and Downregulated Anti-Apoptotic Ones

**Figure 5:**
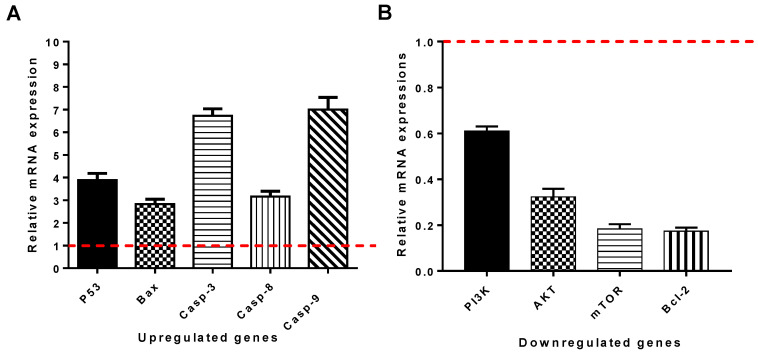
Fold of change of apoptosis-related genes (**A**): Upregulated genes and (**B**): Downregulated genes in untreated and treated MDA-MB-231 cells with compound **7f**. Values are expressed as Mean ± SD of three independent replicates. Data were normalized using β-actin as house-keeping gene. Red dashed line represents the untreated control (Fold change = 1).

To confirm the apoptosis-inducing activity of the compounds in MDA-MB-231 cells, we conducted gene expression analysis using RT-qPCR in both untreated and treated cells. As seen in Figure 5, treatment with compound **7f** increased the expression of the following pro-apoptotic genes: a 3.8-fold increase in P53, a 2.8-fold increase in Bax, and a 6.7-, 3.06-, and 7-fold increase in caspases 3, 8, and 9, respectively. In contrast, this treatment caused a 0.17-fold decrease in the expression of the anti-apoptotic gene Bcl-2. In addition, this treatment induced a 0.61-, 0.32-, and 0.18-fold decrease in the PI3K/AKT/mTOR downstream pathway. These results regarding behavior of upregulating the proapoptotic genes and down- regulating the antiapoptotic gene agreed with previous literatures [50,72,73] on proving apoptosis induction in cancer cells. Apoptosis activity upon treatment with compound **7f**, a derivative of pyrazolyl *s*-triazine moieties, was elucidated via EGFR inhibition and its downstreaming pathway of PI3K/AKT/mTOR.

#### 3.2.6. Molecular Docking Study

**Figure 6:**
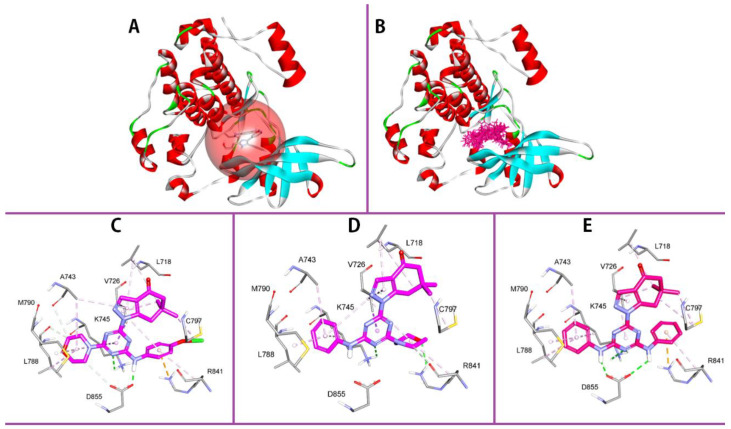
(**A**) X-Ray structure of EGFR in complex with LN2380 ligand (PDB code: 6v6o), with active binding site highlighted in pink. (**B**) The best-docked poses (pink) of the three tested compounds with consensus among at least 8 scoring functions. (**C**–**E**) show the best docking poses of **7d**, **7f**, and **7c** with the interacting amino acids in the active site. The carbon atoms of the ligands are depicted in pink. Hydrogen bonding, hydrophobic interactions, and electrostatic interactions are indicated by the dashed lines in green, light pink, and magenta, respectively. As shown in the figures, the three ligands are involved in a variety of interactions and have similar binding modes.

We implemented CDOCKER, with a binding site sphere of 10.14 Å radius (Figure 6). Docked poses (i.e., 599) generated by CDOCKER were scored by means of the following 9 scoring functions: Jain [57,58], LigScore1, LigScore2 [59], PLP1, PLP2 [57], PMF, PMF04 [60,61], -CDOCKER Energy, and -CDOCKER Interaction Energy [56].

We selected docked conformers/poses based on consensus among the 9 scoring functions [60,61]. The consensus function assigned a value of 1 for any molecular pose ranked within the highest 20% by the particular scoring function; otherwise, a zero value was assigned (i.e., the fit was within the lowest 80%). Docked poses of a particular ligand that achieved consensus among at least 8 scoring functions were selected.

The best-docked poses of **7d**, **7f**, and **7c** interacted with several amino acids in the active site (Figure 6). Interactions included hydrogen bonding, and hydrophobic and electrostatic interactions. The three ligands showed slightly different binding modes in the active site (Figure 6), particularly **7d** and **7f**.

The central triazine ring of the three compounds is involved in hydrogen bonding with Lys745 (K745) and hydrophobic interactions with Val726 (V726). One terminal of the three compounds also participates in hydrophobic interactions and either hydrogen or electrostatic bonding with Arg841 (R841). The other terminals of the three compounds are involved in several hydrophobic interactions with Leu718 (L718), Met790 (M790), Ala743 (A743), and Leu788 (L788). Of note, the binding site of the three compounds is enriched in basic amino acids (K745 and R841) and hydrophobic amino acids (V726, L718, M790, A743, and L788). All the binding poses in Figure 6 show a comparable number and type of interactions (i.e., consensus score ≥ 8), which are believed to play a significant role in the high affinity of these compounds.

## 4. Conclusions

Here, we reported an easy one-pot procedure for the synthesis of pyrazole-*s*-triazine derivatives *via* the reaction of β-dicarbonyl compounds in the presence of DMF-DMA with 4,6-disubstituted 2-hydrazinyl-*s*-triazine in the presence of acetic acid. This method achieved a novel pyrazole and pyrazole-fused cycloalkanones in 80–95% yield and could find applications for the preparation of a variety of pyrazolo-*s*-triazine derivatives with biological activity of interest. Most of the tested compounds showed promising cytotoxicity against a panel of cancer cells and a safe profile against normal cells. Interestingly, compounds **7c**, **7d**, and **7f** induced apoptosis in MDA-MB-231 cells through the EGFR/PI3K/AKT/mTOR signaling pathway. Hence, these compounds emerge as potential target-oriented chemotherapeutic agents against breast cancer.

## Figures and Tables

**Table 2:** IC_50_ values of the tested compounds after the MTT assay showing variable responses of the treated cell lines with different specificity.

Compound	Anticancer Activity in Human Cancer Cells Lines IC_50_ ± STDEV (µM/mL)					
	MDA-MB-231	U-87 MG	PANC-1	A549	MCF-7	HDFs
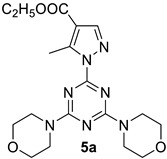	69.4 ± 2.5	65.4 ± 4.2	78.3 ± 7.7	74.6 ± 8.7	57.3 ± 6.2	74.6 ± 9.4
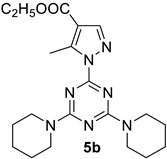	307.1 ± 64.8	355.2 ± 8.5	311.6 ± 30.4	503.4 ± 27.5	306.1 ± 47.6	351.2 ± 52.8
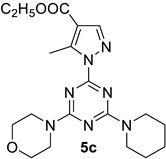	313.8 ± 13.9	840.4 ± 333.8	403.0 ± 47.8	404.0 ± 49.6	249.6 ± 26.7	356.2 ± 14.7
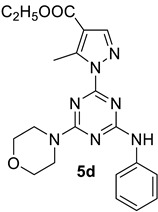	39.4 ± 1.9	42.2 ± 3.4	73.8 ± 21.0	26.4 ± 2.7	10.5 ± 2.4	33.4 ± 3.7
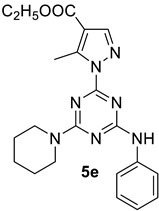	74.7 ± 10.3	193.8 ± 55.5	436.3 ± 59.9	608.4 ± 143.1	242.9 ± 87.9	140.6 ± 5.9
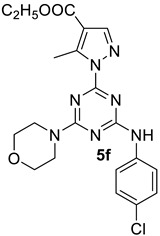	Not converged	10.4 ± 6.1	28.7 ± 7.7	Not converged	207.4 ± 9.5	11.5 ± 2.7
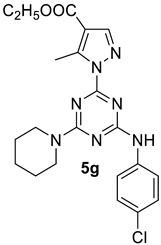	93.1 ± 7.7	206.2 ± 19.7	169.0 ± 10.9	202.5 ± 9.3	186.9 ± 34.8	155.5 ± 19.5
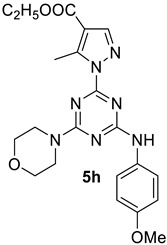	29.7 ± 1.8	76.7 ± 26.4	57.6 ± 9.8	592.1 ± 31.9	46.6 ± 11.1	126.1 ± 29.6
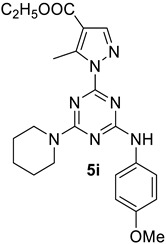	110.7 ± 20.8	330.5 ± 84.6	183.1 ± 30.2	249.4 ± 87.5	328.5 ± 92.6	170.3 ± 30.6
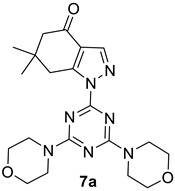	Not converged	Not converged	Not converged	Not converged	431.9 ± 58.5	Not converged
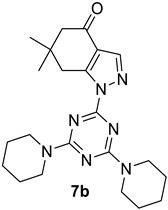	338.4 ± 114.5	292.8 ± 26.1	472.9 ± 73.3	228.5 ± 24.7	199.5 ± 13.2	3779.9 ± 627.3
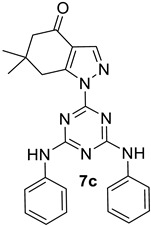	28.2 ± 4.4	17.1 ± 4.2	16.4 ± 11.8	49.8 ± 4.5	15.7 ± 3.1	245.6 ± 22.8
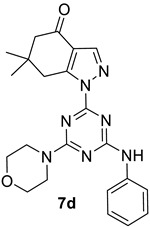	97.3 ± 9.3	10.9 ± 4.1	26.7 ± 8.3	12.4 ± 6.4	8.3 ± 2.1	38.9 ± 8.3
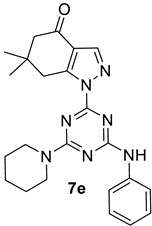	47.4 ± 13.2	73.1 ± 32.6	Not converged	89.8 ± 22.8	132.2 ± 28.0	47.4 ± 8.9
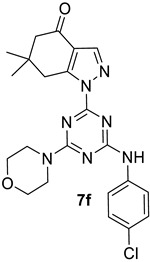	21.1 ± 2.6	41.6 ± 14.9	32.8 ± 12.3	52.0 ± 10.8	27.0 ± 11.0	65.0 ± 15.6
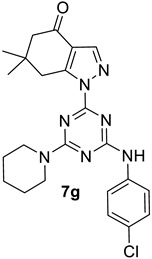	Not converged	Not converged	Not converged	Not converged	175.0 ± 93.6	4.2 ± 2.0
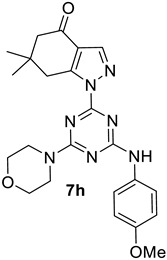	402.2 ± 11.6	Not converged	37.7 ± 7.1	Not converged	206.2 ± 15.8	316.8 ± 23.4
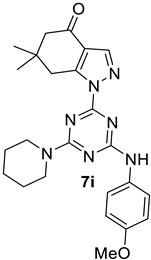	567.9 ± 67.5	348.4 ± 126.6	939.3 ± 204.1	579.6 ± 285.1	25.8 ± 89.4	2922.4 ± 251.1
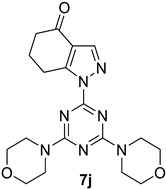	256.6 ± 51.1	Not converged	339.6 ± 67.2	507.7 ± 57.3	529.8 ± 50.3	1326.6 ± 128.2
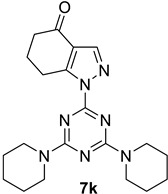	437.5 ± 105.1	Not converged	515.9 ± 79.2	379.8 ± 90.2	290.2 ± 37.0	1337.7 ± 144.4
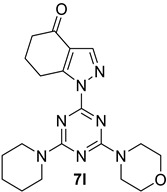	Not converged	Not converged	Not converged	1744.1 ± 126.7	654.0 ± 144.2	638.7 ± 78.0
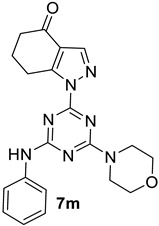	90.6 ± 13.0	74.7 ± 21.4	61.3 ± 16.9	43.4 ± 12.5	34.2 ± 4.1	117.0 ± 28.4
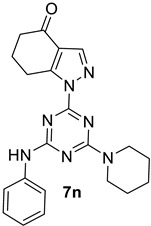	126.3 ± 41.9	185.4 ± 52.9	Not converged	92.9 ± 12.1	368.7 ± 42.1	189.5 ± 50.3
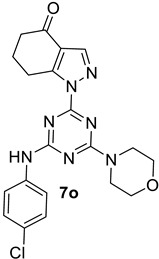	464.2 ± 105.7	51.4 ± 26.3	Not converged	85.7 ± 47.9	228.2 ± 100.0	54.5 ± 26.3
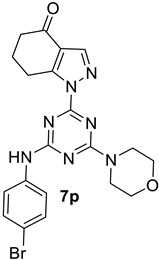	175.4 ± 64.0	345.1 ± 58.0	4.46 ± 2.3	Not converged	17.0 ± 56.3	93.8 ± 40.2
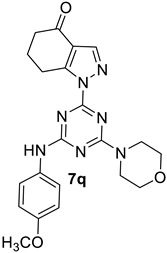	845.2 ± 205.0	Not converged	Not converged	179.4 ± 78.8	3430.9 ± 481.7	Not converged
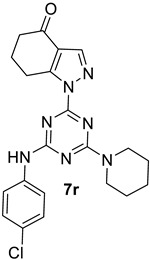	16.1 ± 2.5	44.8 ± 3.5	60.0 ± 8.4	83.9 ± 12.1	2182.0 ± 221.7	20.7 ± 6.3
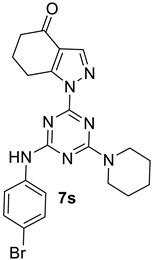	40.3 ± 9.3	71.5 ± 15.7	47.5 ± 2.6	18.6 ± 2.9	27.0 ± 4.7	74.7 ± 9.9
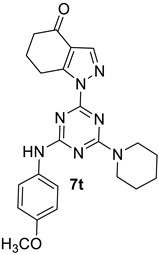	60.2 ± 12.7	269.0 ± 33.8	807.6 ± 165.3	12.4 ± 1.1	Not converged	122.9 ± 16.7
**Doxorubicin**	0.287 ± 0.035	0.057 ± 0.033	2.99 ± 1.56	0.278 ± 0.107	0.055 ± 0.0183	0.423 ± 0.270

**Table 3:** IC_50_ values of the compounds tested in the EGFR-PK assay.

Compound	EGFR-PK Inhibition, IC_50_ [nM] *^,#^
**7d**	70.3 ± 1.34
**7f**	59.24 ± 1.16
**7c**	81.6 ± 1.67
**Tamoxifen**	69.1 ± 1.39

* Values are expressed as Mean ± SD of three independent replicates. ^#^ IC_50_ values were calculated using sigmoidal non-linear regression curve fit of percentage inhibition against five concentrations of each compound.

**Table 4:** Activity of the tested compounds **7d**, **7f**, and **7c**, against the EGFR downstream signaling pathway (PI3K/AKT/mTOR) in untreated and treated MDA-MB-231 cells.

Compound	PI3K (ng/mL)	AKT (ng/mL)	mToR (ng/mL)
**Control**	6.64 ± 0.15	45.39 ± 0.68	86.39 ± 2.1
**7d**	4.39 ± 0.16	37.3 ± 0.69	69.3 ± 1.98
**7f**	2.39 ± 0.12	25.34 ± 0.39	57.6 ± 1.23
**7c**	6.1 ± 0.36	42.6.3 ± 0.49	86.3 ± 1.67

Values are expressed as Mean ± SD of three independent replicates.

## Data Availability

Not applicable.

## Supplementary Materials

The following supporting information can be downloaded at: https://www.mdpi.com/article/10.3390/pharmaceutics14081558/s1, X-Ray determination of compound **7t**; Figures S1–S29: Selected NMR and MS spectrum data for the synthesized compounds **5a**–**i** and **7a**–**t**; Figures S30–S38: Selected HRMS spectrum data for some of the synthesized compounds **5** and **7** series; and Figure S39. Flow cytometric analysis (Annexin V-FTIC/PI assay).
