# Supplementary material for: Acetic Acid Mediated for One-Pot Synthesis of Novel Pyrazolyl s-Triazine Derivatives for the Targeted Therapy of Triple-Negative Breast Tumor Cells (MDA-MB-231) via EGFR/PI3K/AKT/mTOR Signaling Cascades
Source: Pharmaceutics. 2022 Jul 27;14(8):1558. doi: 10.3390/pharmaceutics14081558 (PMC9414415; doi:10.3390/pharmaceutics14081558)
Supplement: Supplementary file 1 [file pharmaceutics-14-01558-s001.zip › pharmaceutics-1804260-supplementary.pdf]

SUPPORTING INFORMATION

Acetic Acid Mediated for One-Pot Synthesis of Novel Pyrazolyl *s*-Triazine Derivatives for the Targeted Therapy of Triple-Negative Breast Tumor Cells (MDA-MB-231) *via* EGFR/PI3K/AKT/mTOR Signaling Cascades

Ihab Shawish [1,2], Assem Barakat [2,*], Ali Aldalbahi [2], Walhan Alshaer [3], Fadwa Daoud [3], Dana A. Alqudah [3], Mazhar Al Zoubi [4], Ma'mon M. Hatmal [5], Mohamed S. Nafie [6], Matti Haukka [7], Anamika Sharma [8,9], Beatriz G. de la Torre [8,9], Fernando Albericio [9,10,11,*] and Ayman El-Faham [12,*]

1 Department of Math and Sciences, College of Humanities and Sciences, Prince Sultan University, P.O. Box 66833, Riyadh 11586, Saudi Arabia; ishawish@psu.edu.sa (I. S.)
2 Department of Chemistry, College of Science, King Saud University, P.O. Box 2455, Riyadh 11451, Saudi Arabia; aaldalbahi@ksu.edu.sa (A.A.)
3 Cell Therapy Center, The University of Jordan, Amman 11942, Jordan; walhanjordan@yahoo.com (W.A.); fadwadaoud22@gmail.com (F.D.); pharmd.dana.alqudah@gmail.com (D.A.A.)
4 Department of Basic Medical Sciences, Faculty of Sciences, Yarmouk University, Irbid 21163, Jordan; mszoubi@yu.edu.jo
5 Department of Medical Laboratory Sciences, Faculty of Applied Medical Sciences, The Hashemite University, P.O. Box 330127, Zarqa 13133, Jordan; mamon@hu.edu.jo
6 Department of Chemistry, Faculty of Science, Suez Canal University, Ismailia 41522, Egypt; mohamed_nafie@science.suez.edu.eg
7 Department of Chemistry, University of Jyväskylä, P.O. Box 35, FI-40014 Jyväskylä, Finland; matti.o.haukka@jyu.fi
8 KwaZulu-Natal Research Innovation and Sequencing Platform (KRISP), School of Laboratory Medicine and Medical Sciences, College of Health Sciences, University of KwaZulu-Natal, Durban 4041, South Africa; anamika.aug14@gmail.com (A.S.); garciadelatorreb@ukzn.ac.za (B.G.d.l.T.)
9 Peptide Science Laboratory, School of Chemistry and Physics, University of KwaZulu-Natal, Durban 4001, South Africa.
10 CIBER-BBN (Networking Centre on Bioengineering, Biomaterials and Nanomedicine) and Department of Organic Chemistry, University of Barcelona, 08028 Barcelona, Spain
11 Institute for Advanced Chemistry of Catalonia (IQAC-CSIC), 08034 Barcelona, Spain
12 Chemistry Department, Faculty of Science, Alexandria University, P.O. Box 426, Ibrahimia, Alexandria 12321, Egypt.
* Correspondence: ambarakat@ksu.edu.sa (A.B.); albericio@ukzn.ac.za (F.A.); ayman.elfaham@alexu.edu.eg or aymanel_faham@hotmail.com (A.E.-F.)

Table of content

1. **X-Ray structure determinations**
2. **Selected copy of the (^{1}HNMR, ^{13}CNMR and MS) spectrum of the synthesized compounds.**

**Figure S1**	**^{1}H NMR of 5d and 5d′ mixture in low acetic acid load**
**Figure S2**	**^{1}H and ^{13}C compound 5a**
**Figure S3**	**^{1}H and ^{13}C compound 5b**
**Figure S4**	**^{1}H and ^{13}C compound 5c**
**Figure S5**	**^{1}H and ^{13}C compound 5e**
**Figure S6**	**^{1}H and ^{13}C compound 5f**
**Figure S7**	**^{1}H and ^{13}C compound 5g**
**Figure S8**	**^{1}H and ^{13}C compound 5h**
**Figure S9**	**^{1}H and ^{13}C compound 5i**
**Figure S10**	**^{1}H and ^{13}C compound 7a**
**Figure S11**	**^{1}H and ^{13}C compound 7b**
**Figure S12**	**^{1}H and ^{13}C compound 7c**
**Figure S13**	**^{1}H and ^{13}C compound 7d**
**Figure S14**	**^{1}H and ^{13}C compound 7e**
**Figure S15**	**^{1}H and ^{13}C compound 7f**
**Figure S16**	**^{1}H and ^{13}C compound 7g**
**Figure S17**	**^{1}H and ^{13}C compound 7h**
**Figure S18**	**^{1}H and ^{13}C compound 7i**
**Figure S19**	**^{1}H and ^{13}C compound 7j**
**Figure S20**	**^{1}H and ^{13}C compound 7k**
**Figure S21**	**^{1}H and ^{13}C compound 7l**
**Figure S22**	**^{1}H and ^{13}C compound 7m**
**Figure S23**	**^{1}H and ^{13}C compound 7n**
**Figure S24**	**^{1}H and ^{13}C compound 7o**
**Figure S25**	**^{1}H and ^{13}C compound 7p**
**Figure S26**	**^{1}H and ^{13}C compound 7q**
**Figure S27**	**^{1}H and ^{13}C compound 7r**
**Figure S28**	**^{1}H and ^{13}C compound 7s**

Figure S29 ^{1}H and ^{13}C compound 7t
Figure S30 HRMS analysis for compound 5a
Figure S31 HRMS analysis for compound 5b
Figure S32 HRMS analysis for compound 5c
Figure S33 HRMS analysis for compound 5f
Figure S34 HRMS analysis for compound 7a
Figure S35 HRMS analysis for compound 7b
Figure S36 HRMS analysis for compound 7c
Figure S37 HRMS analysis for compound 7h
Figure S38 HRMS analysis for compound 7i

3. **Flow cytometric analysis (Annexin V-FTIC/PI assay).**
Figure S39: Flow cytometric analysis (Annexin V-FTIC/PI assay) MDA-MB-231, MCF-7, U87 MG, A459 and PANC1 cancer cell lines compared to normal cell line HDF for 24 h **7d, 7f,** and **7c**. The represented dot plots showing percentage of viable, early apoptotic, late apoptotic, and necrotic cells. in. The results showed a higher percentage in the apoptosis mechanism induced in all cancer cell lines compared to the normal cell line.

1. X-Ray structure determinations

The crystal of **7t** was immersed in cryo-oil, mounted in a loop, and measured at a temperature of 120 K. The X-ray diffraction data was collected on a Rigaku Oxford Diffraction Supernova diffractometer using Mo Kα radiation. The *CrysAlisPro* [S1] software package was used for cell refinement and data reduction. An analytical absorption correction (*CrysAlisPro* [S1]) was applied to the intensities before structure solution. Structure was solved by intrinsic phasing (*SHELXT* [S2]) method. Structural refinement was carried out using *SHELXL* [S3] software with *SHELXLE* [S4] graphical user interface. The carbon atoms C24 C25 C26 and C27 were disordered over two sites with occupancy ratio of 0.77/0.23. The disordered atom pairs were refined with equal anisotropic displacement parameters. The NH hydrogen atom was located from the difference Fourier map and refined isotropically. All other hydrogen atoms were positioned geometrically and constrained to ride on their parent atoms, with C-H = 0.95 – 0.99 Å and U_{iso} = 1.2-1.5·U_{eq}(parent atom). The crystallographic details are summarized in Table **S1**.

Table S1. Crystal Data of **7t**.

	**7t**
CCDC	2177427
empirical formula	$C_{24}H_{31}N_7O_3S$
fw	497.62
temp (K)	120(2)
λ(Å)	0.71073
cryst syst	Monoclinic
space group	*C2/c*
a (Å)	17.8511(5)
b (Å)	13.9801(3)
c (Å)	20.2210(5)
β (deg)	100.498(3)
V (Å^3)	4961.9(2)
Z	8
ρ_{calc} (Mg/m^3)	1.332
μ(Mo Kα) (mm^{-1})	0.171
No. reflns.	23722
Unique reflns.	6141
Completeness to θ=25.242°	99.7%
GOOF (F^2)	1.141
R_{int}	0.0306
R_1^{a} ($I \geq 2\sigma$)	0.0610
wR_2^{b} ($I \geq 2\sigma$)	0.1582

^{a} $R_1 = \Sigma||F_o| - |F_c||/\Sigma|F_o|$. ^{b}$wR_2 = \{\Sigma[w(F_o^2 - F_c^2)^2]/\Sigma[w(F_o^2)^2]\}^{1/2}$

References

S1. Rikagu Oxford Diffraction. *CrysAlisPro*; Agilent Technologies Inc.: Oxfordshire, UK, 2018.

S2. Sheldrick, G.M. SHELXT-Integrated Space-Group and Crystal-Structure Determination. *Acta Crystallogr. Sect. A Found. Adv.* **2015**, *71*, 3–8.

S3. Sheldrick, G.M. Crystal Structure Refinement with SHELXL. *Acta Crystallogr. Sect. C Struct. Chem.* **2015**, *71*, 3–8.

S4. Hübschle, C.B.; Sheldrick, G.M.; Dittrich, B. *ShelXle*: A Qt graphical user interface for *SHELXL*. *J. Appl. Crystallogr.* **2011**, *44*, 1281–1284.

2. NMR and MS Spectrum

Figure S1. ^{1}H NMR of 5d and 5d' mixture (reaction conditions: 5%AcOH-EtOH, reflux 4-8 h)

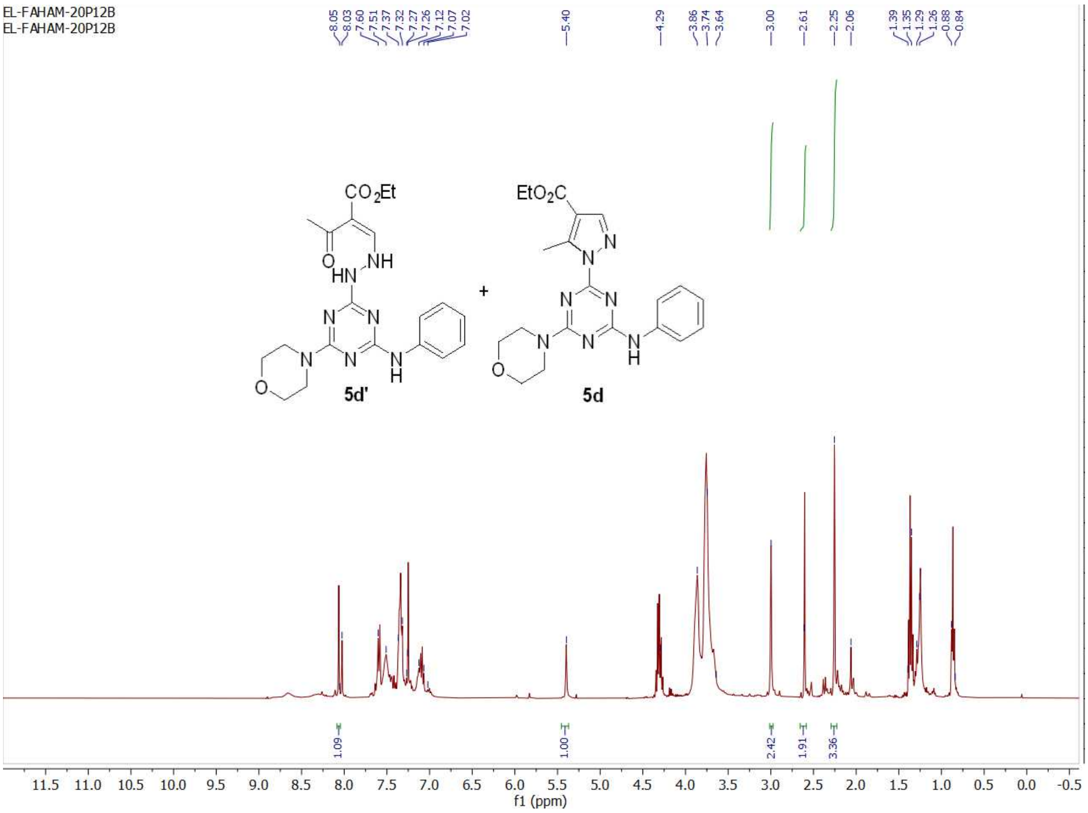

Figure S2. [1]H and ^{13}C compound 5a

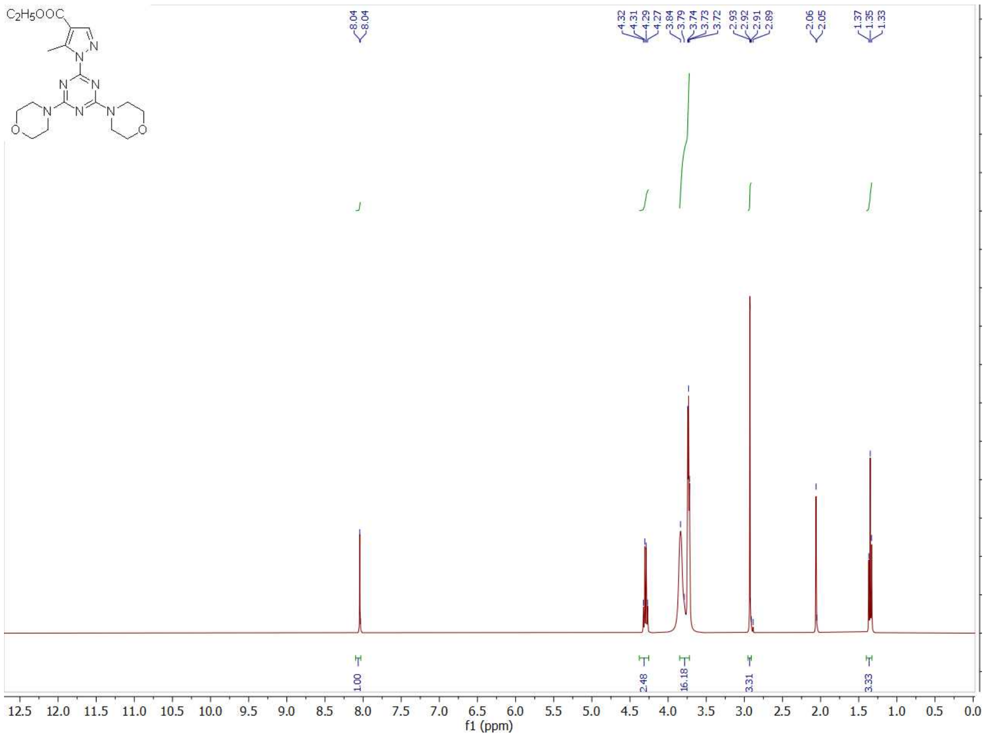

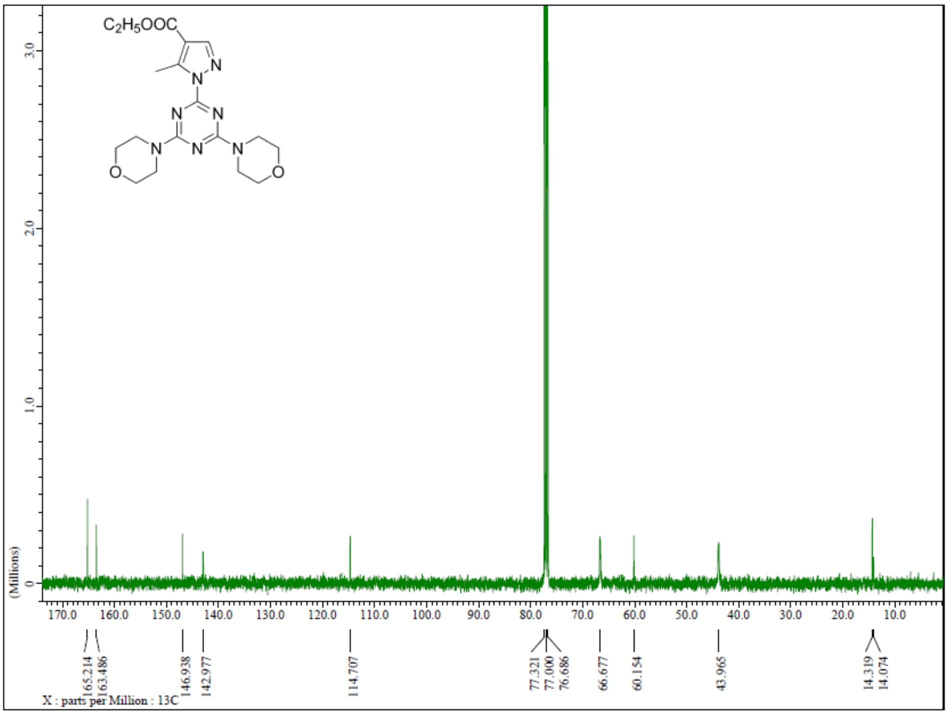

Figure S3. ^{1}**H and** ^{13}**C compound 5b**

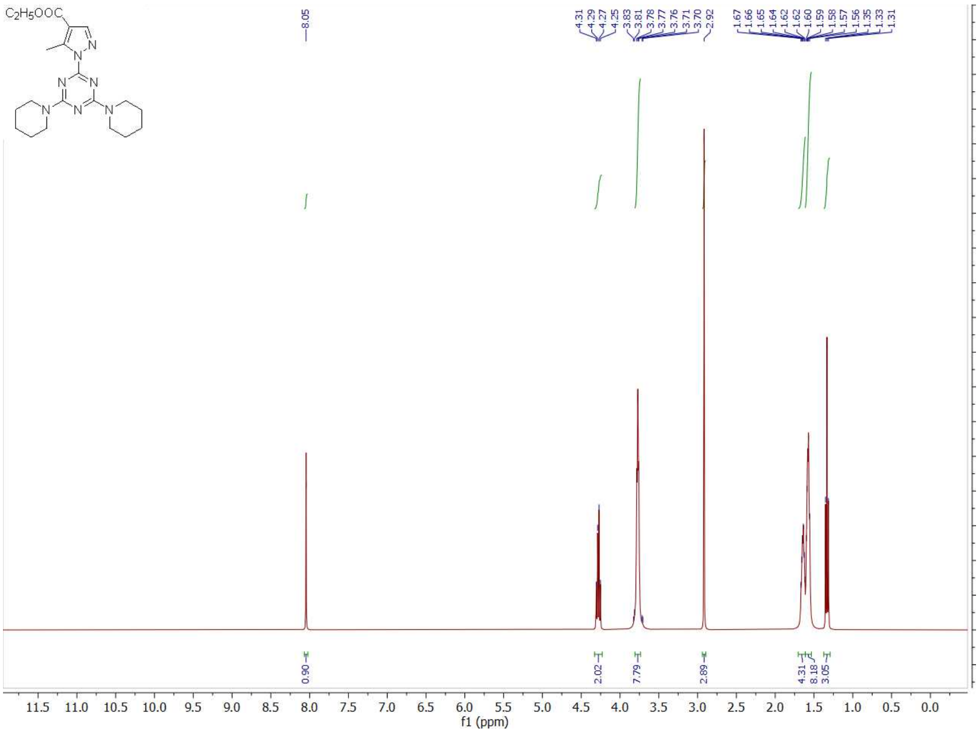

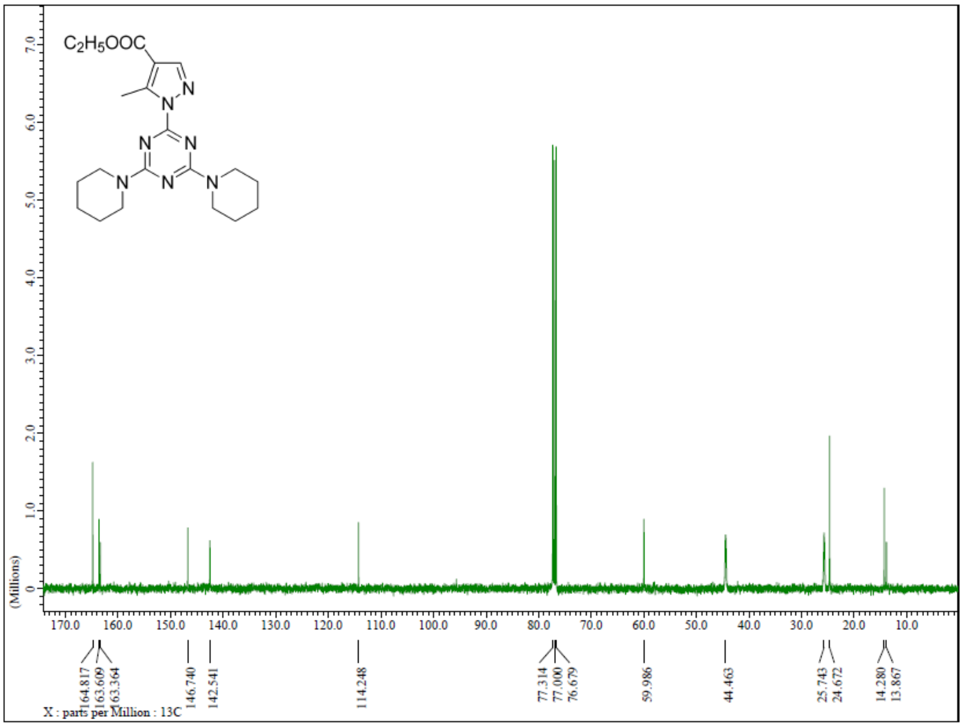

Figure S4. ^{1}H and ^{13}C compound 5c

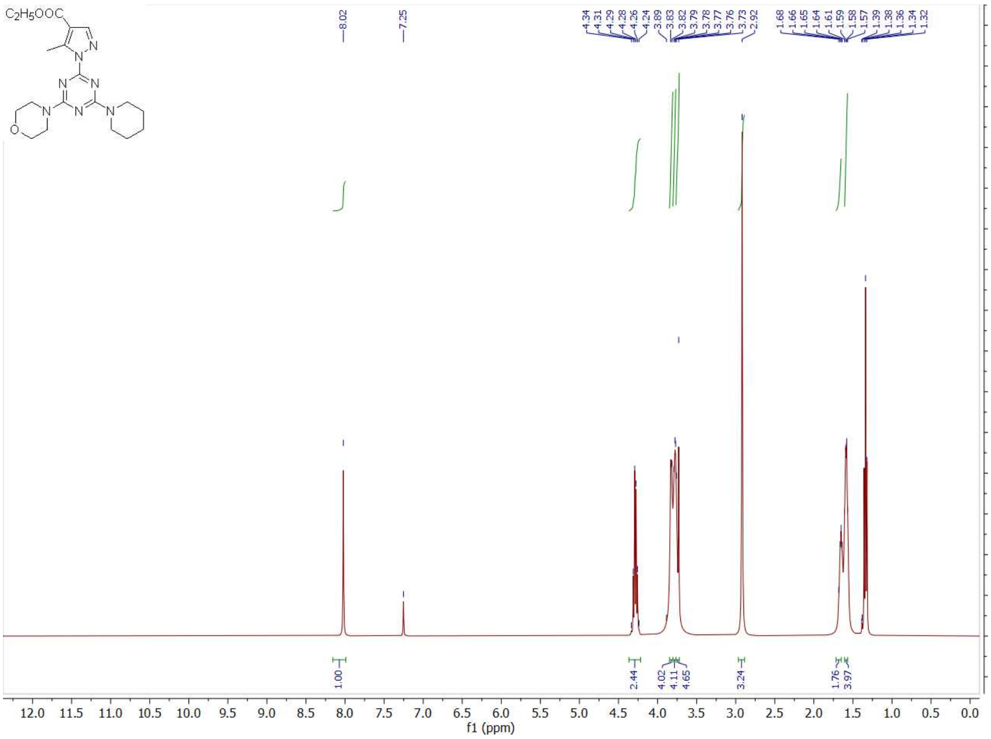

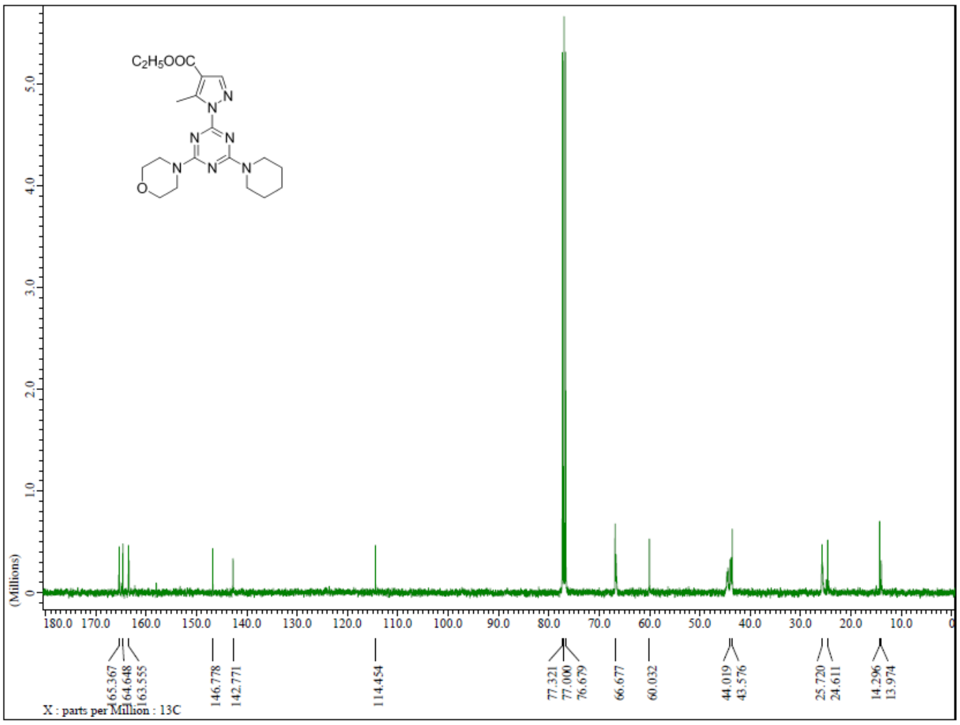

Figure S5. ^{1}H and ^{13}C compound 5e

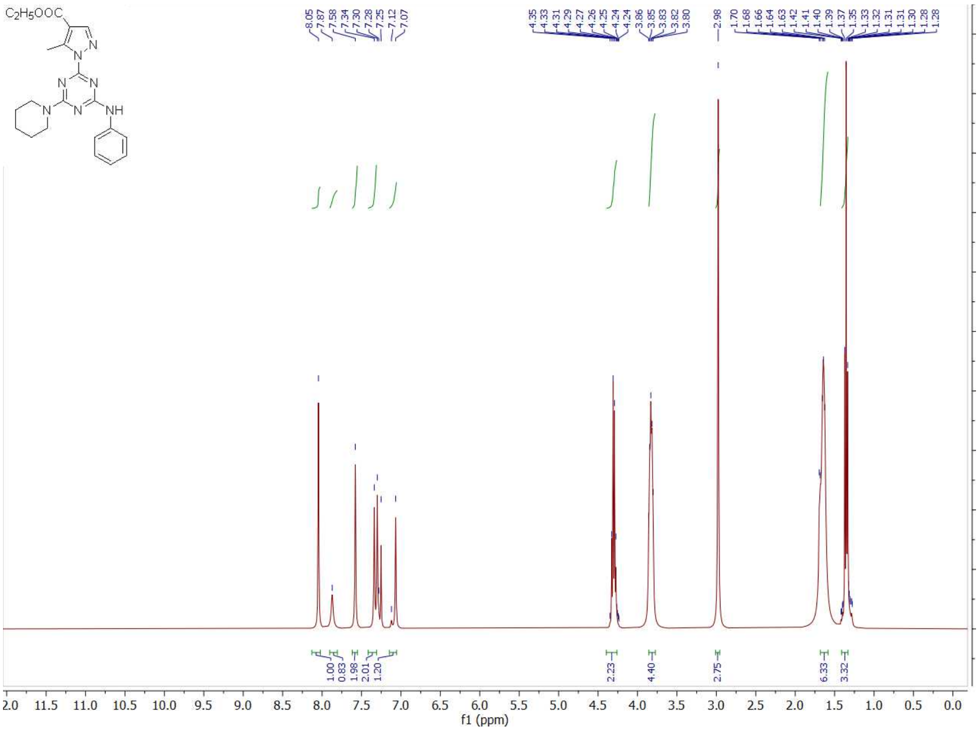

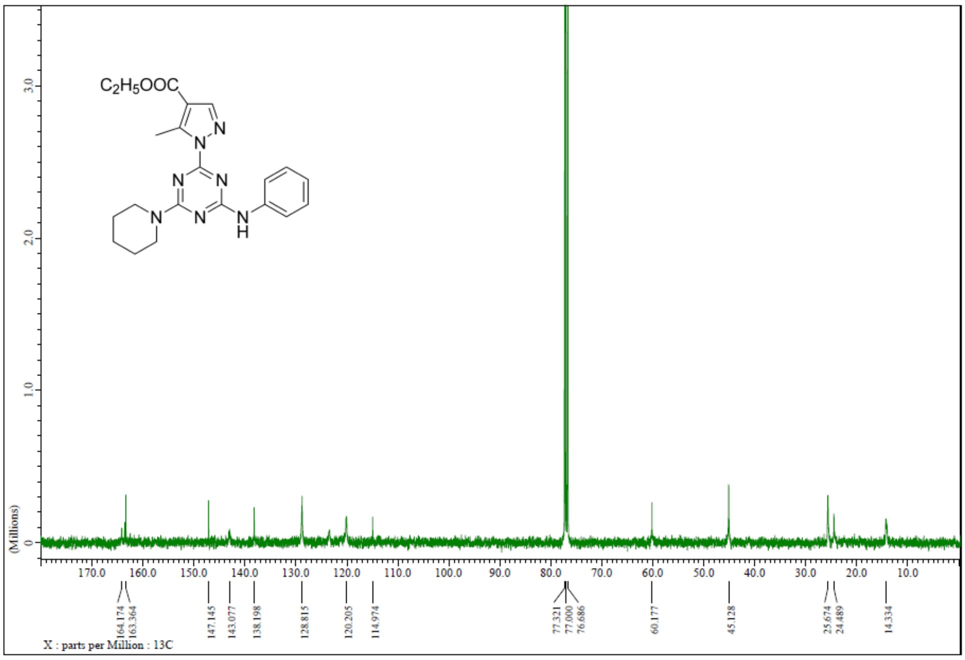

Figure S6.[1]H and ^{13}C compound 5f

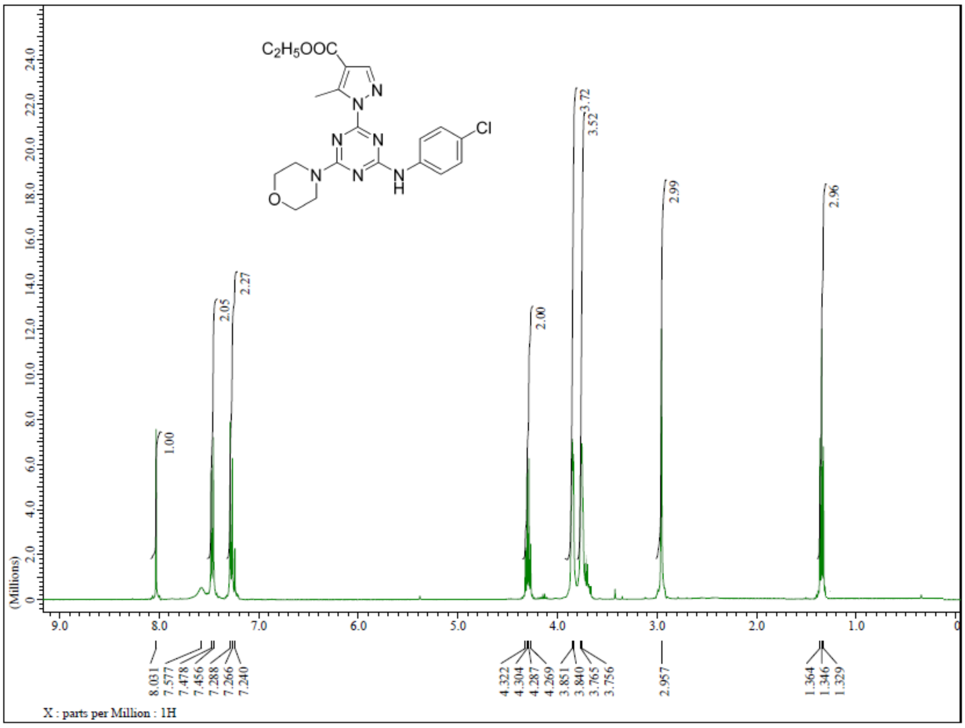

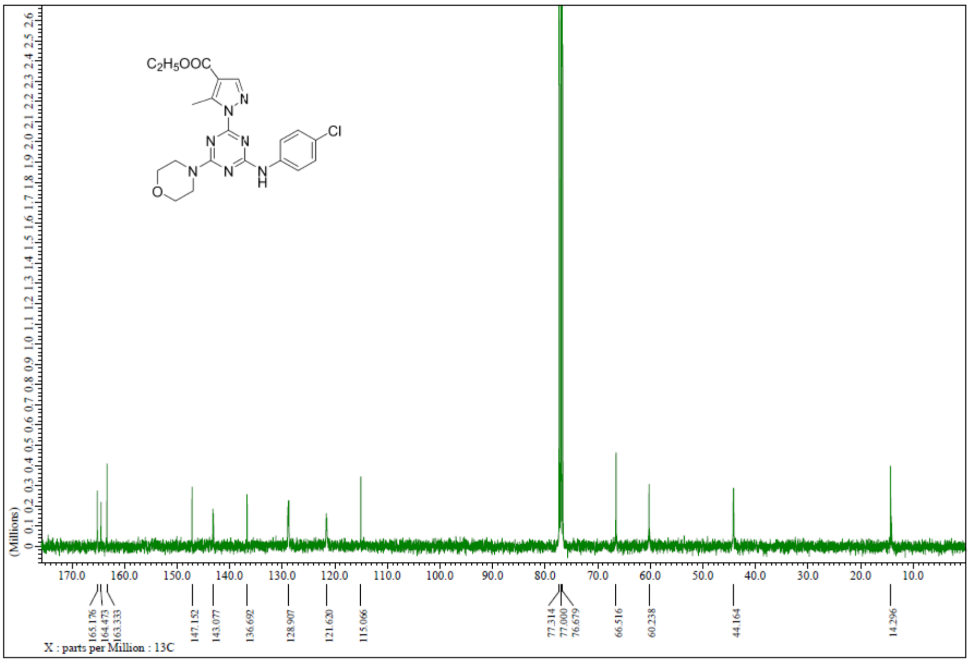

Figure S7. [1]H and ^{13}C compound 5g

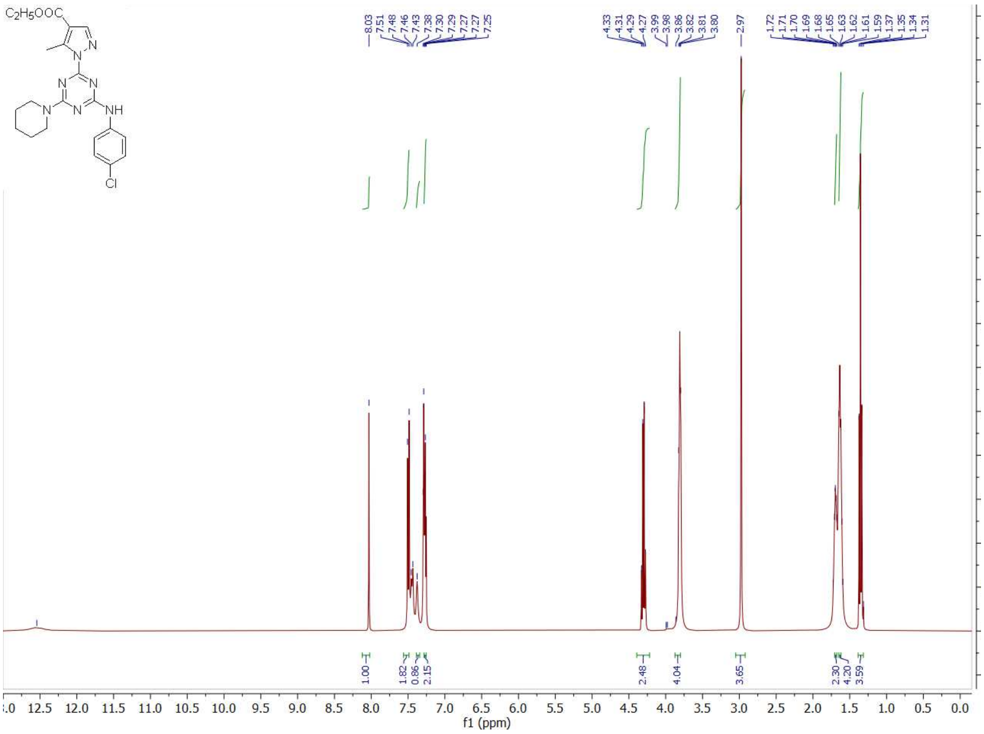

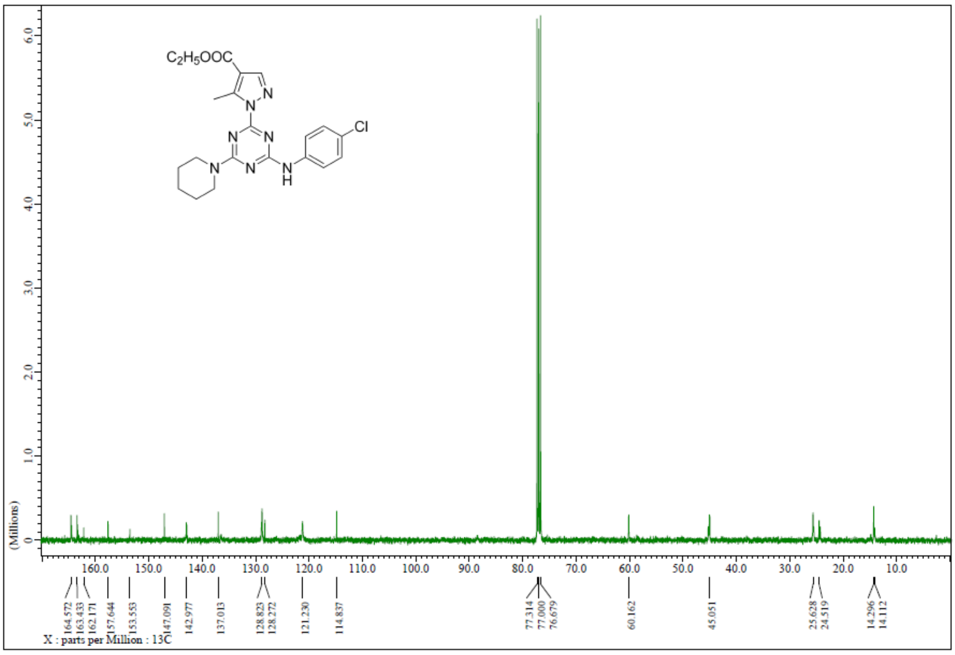

Figure S8. ^{1}H and ^{13}C compound 5h

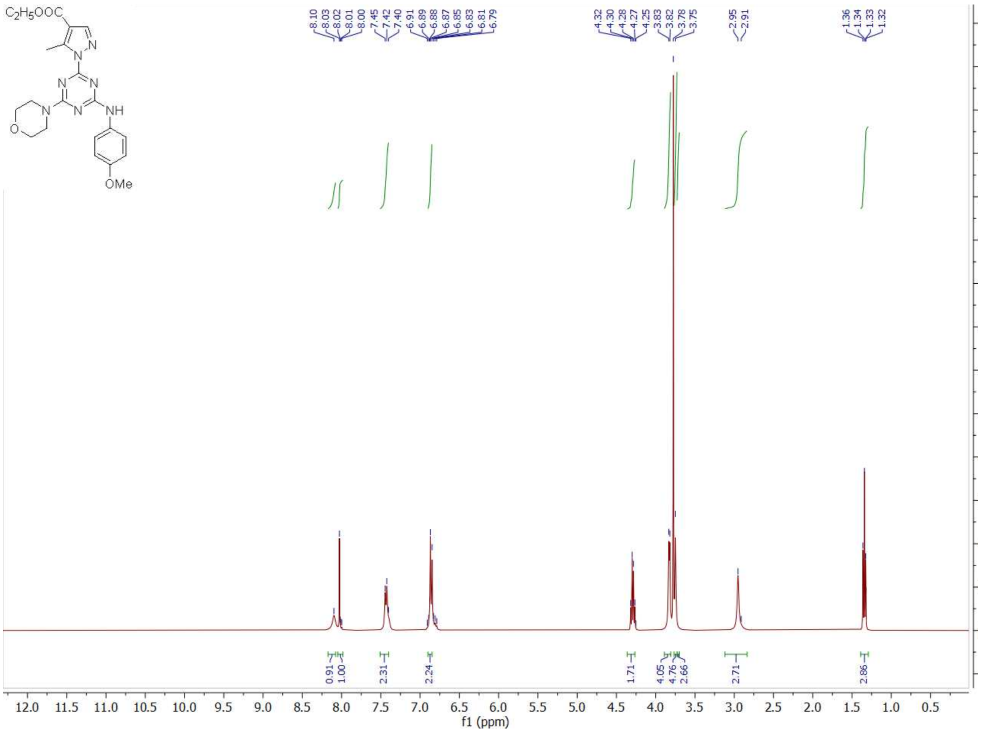

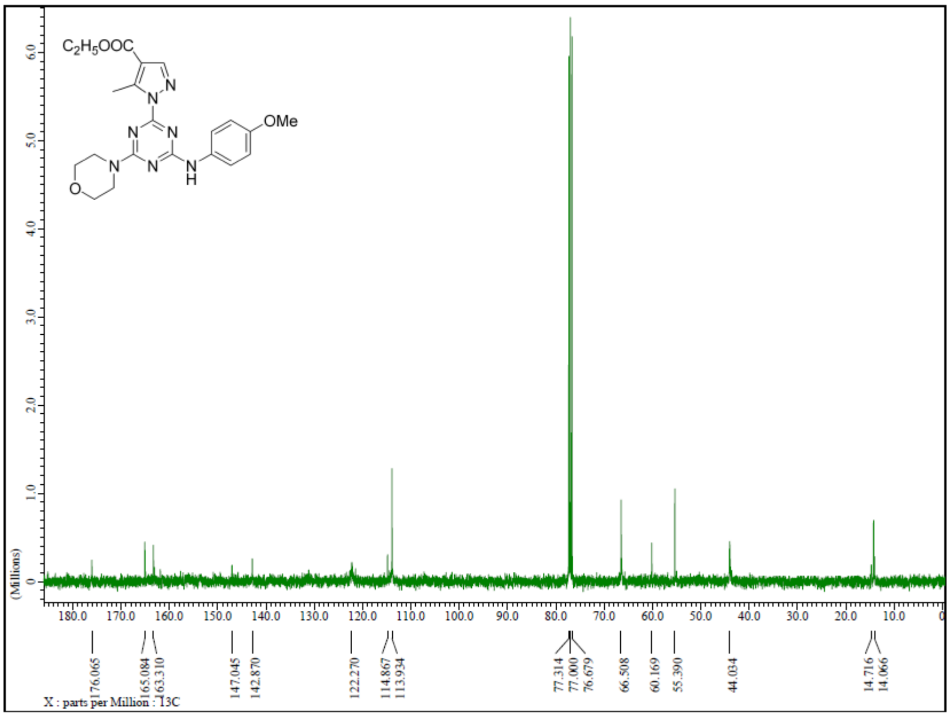

Figure S9. [1]H and ^{13}C compound 5i

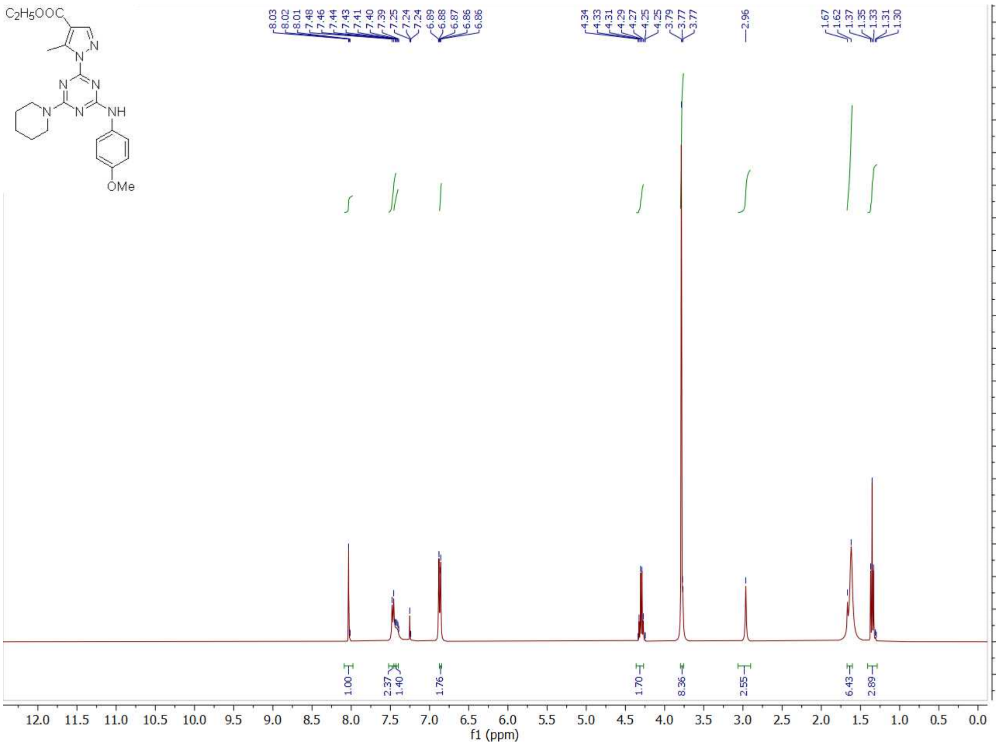

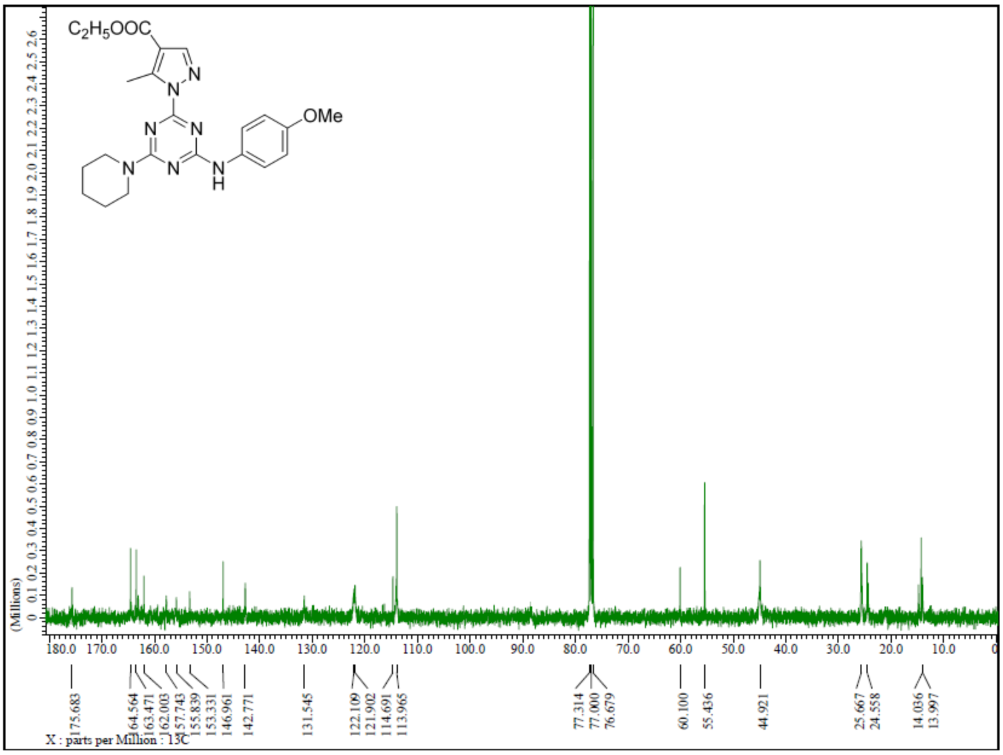

Figure S10. ^{1}H and ^{13}C compound 7a

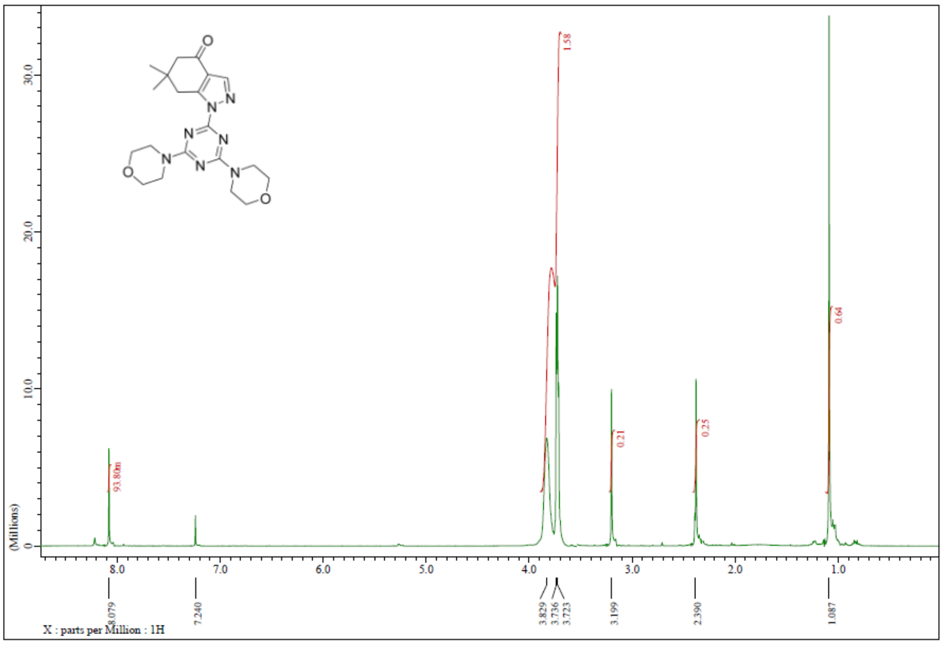

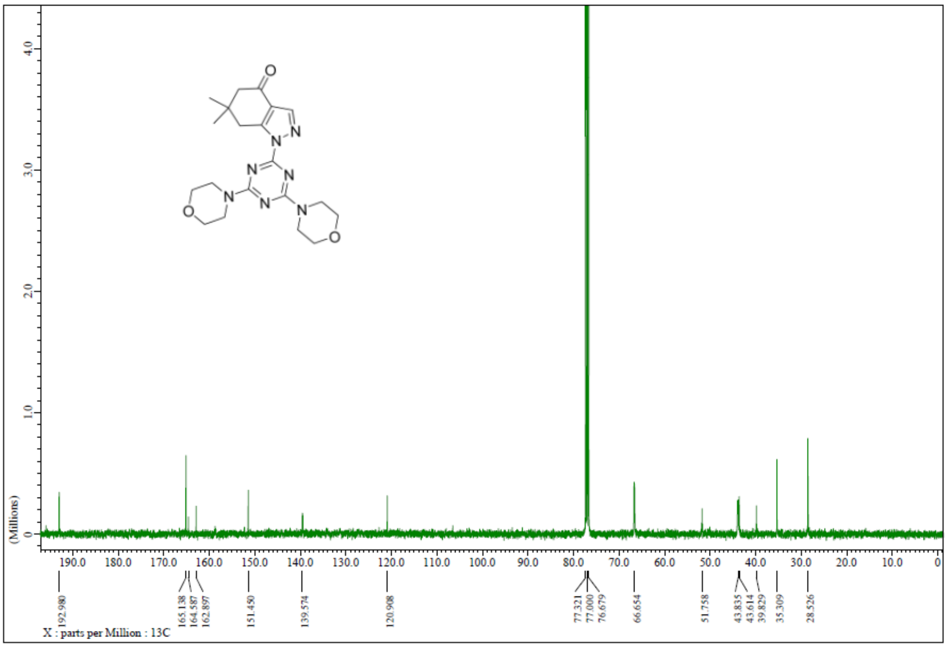

Figure S11. ^{1}H and ^{13}C compound 7b

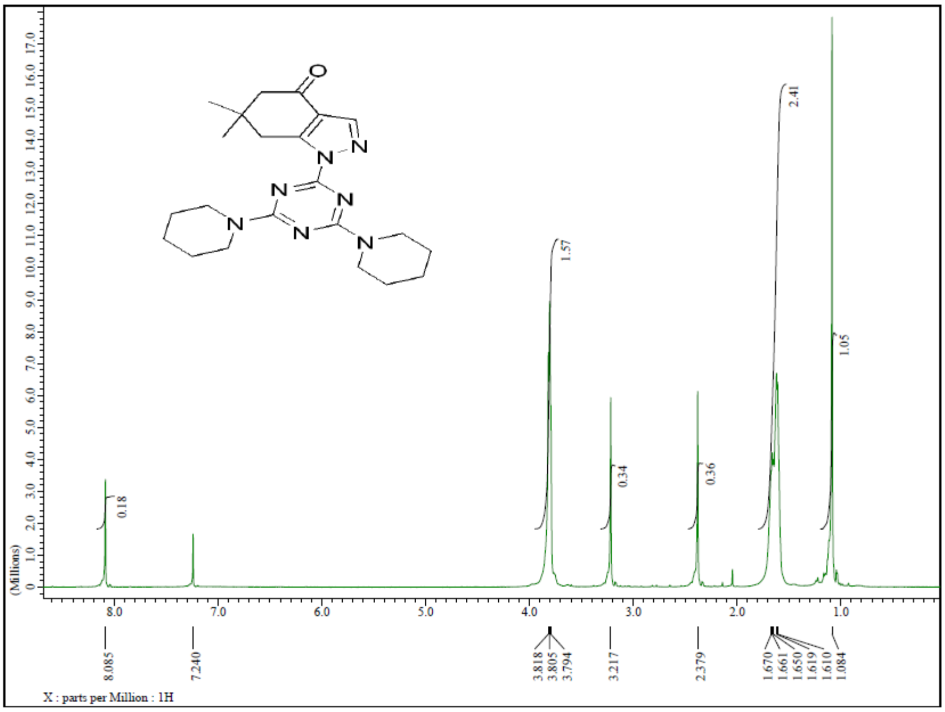

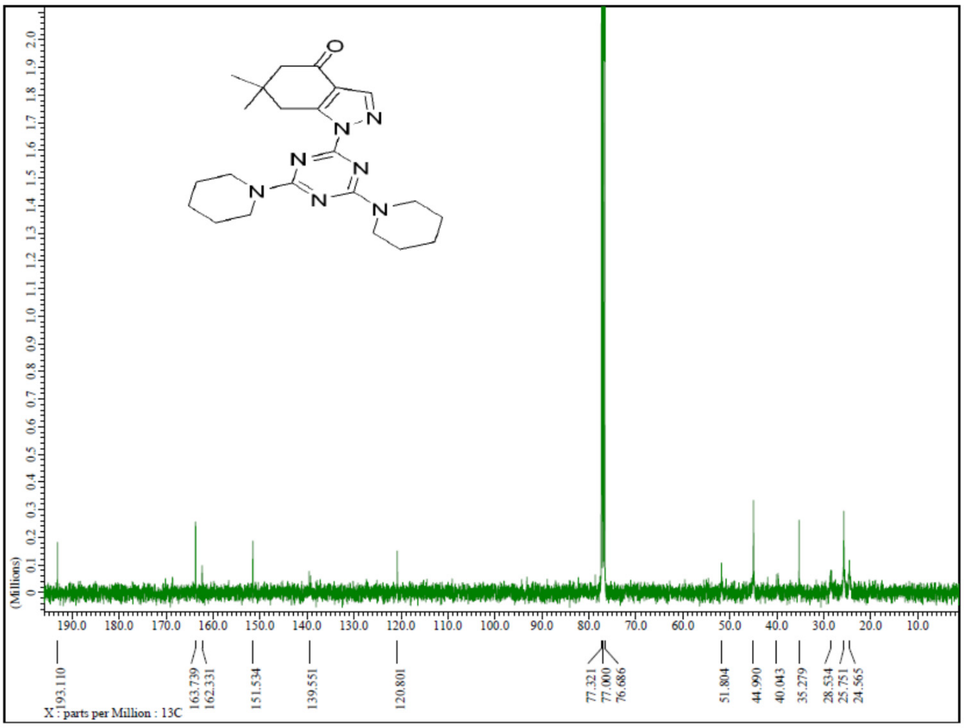

Figure S12. ^{1}H and ^{13}C compound 7c

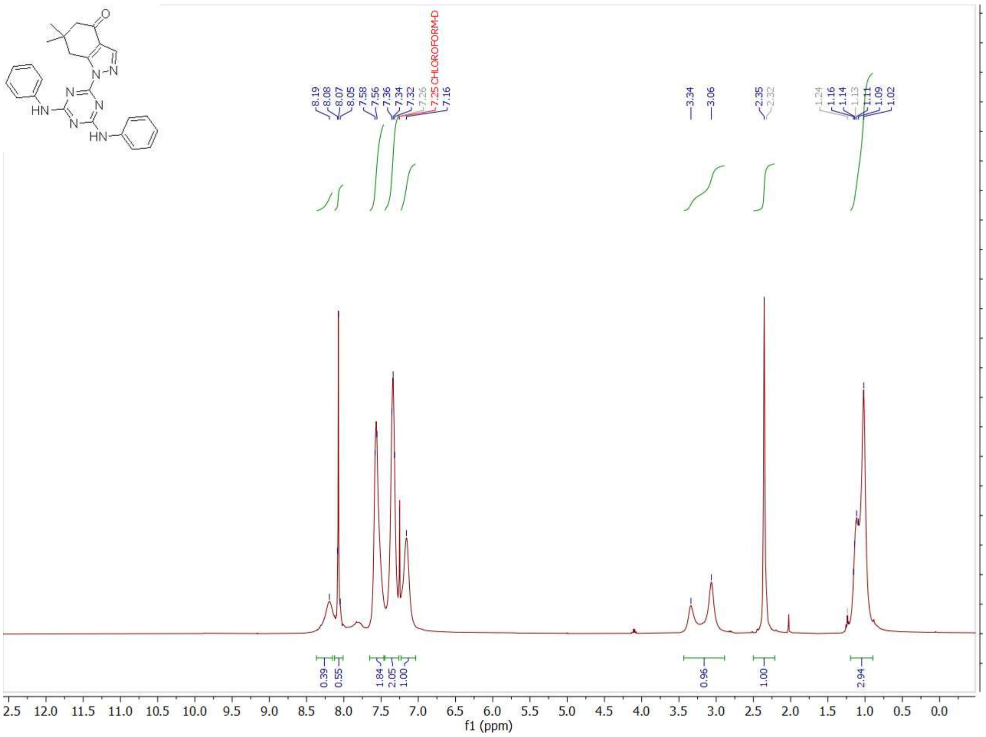

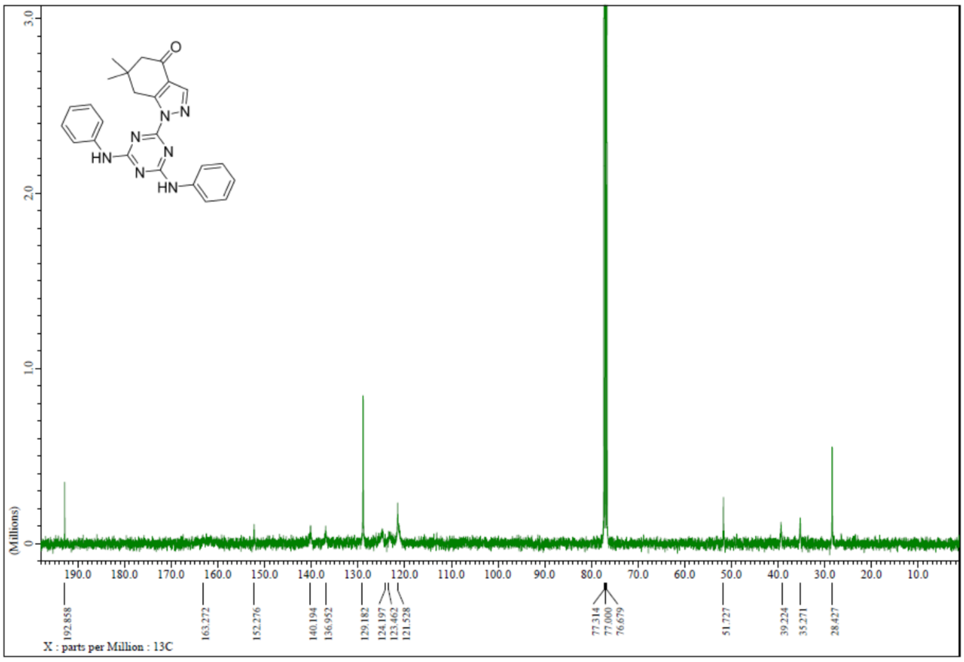

Figure S13. [1]H and ^{13}C compound 7d

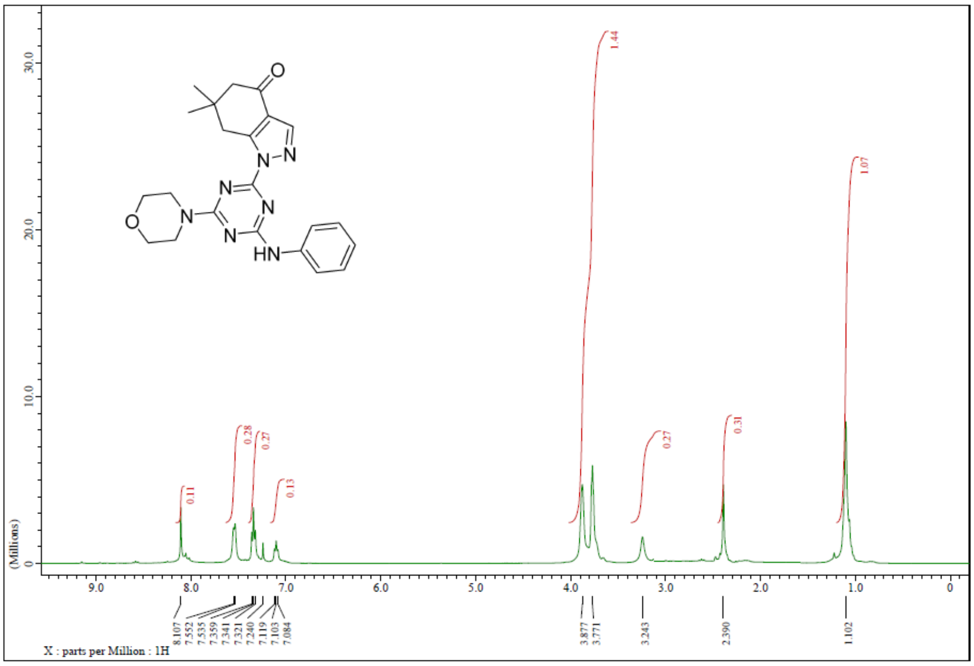

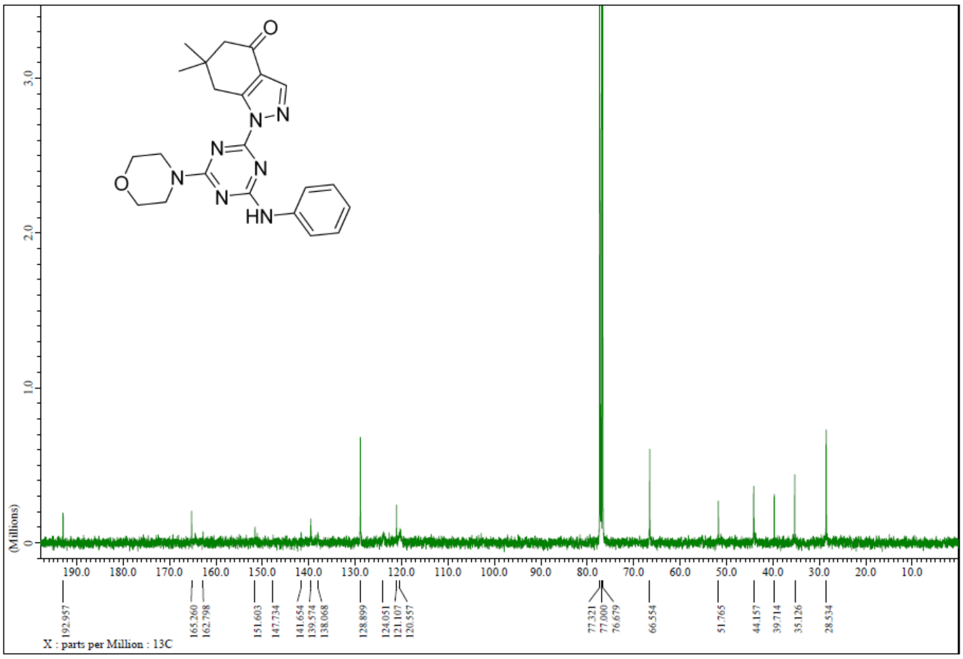

Figure S14. [1]H and ^{13}C compound 7e

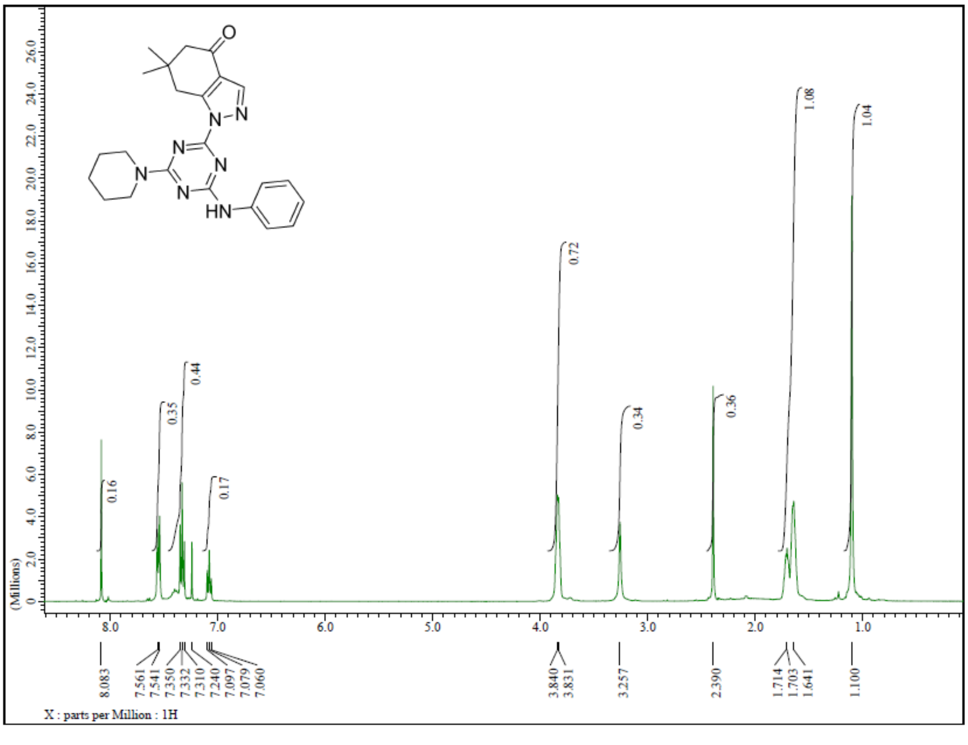

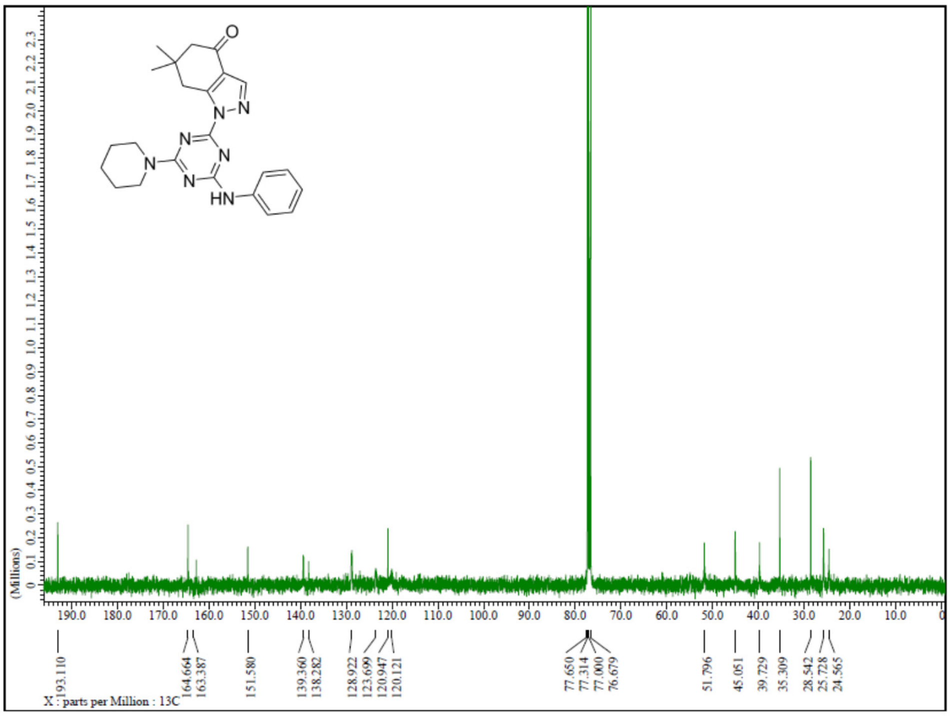

Figure S15. [1]H and ^{13}C compound 7f

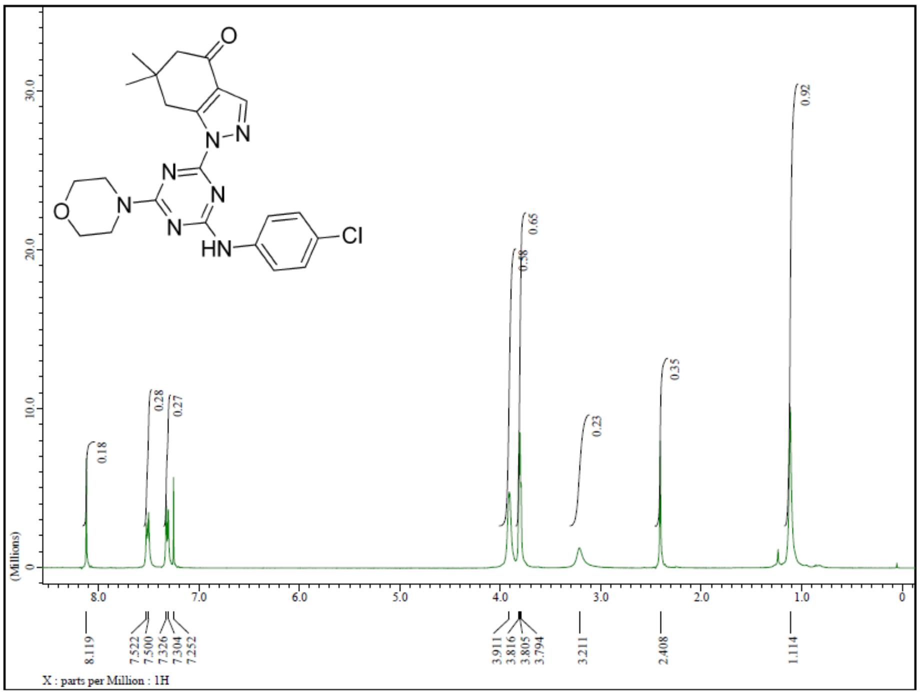

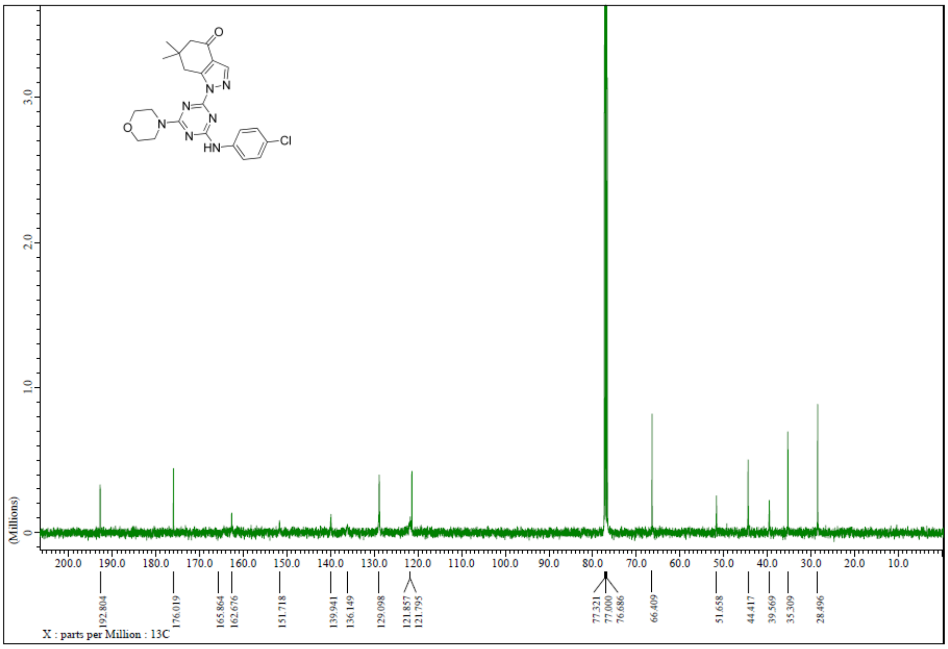

Figure S16. [1]H and ^{13}C compound 7g

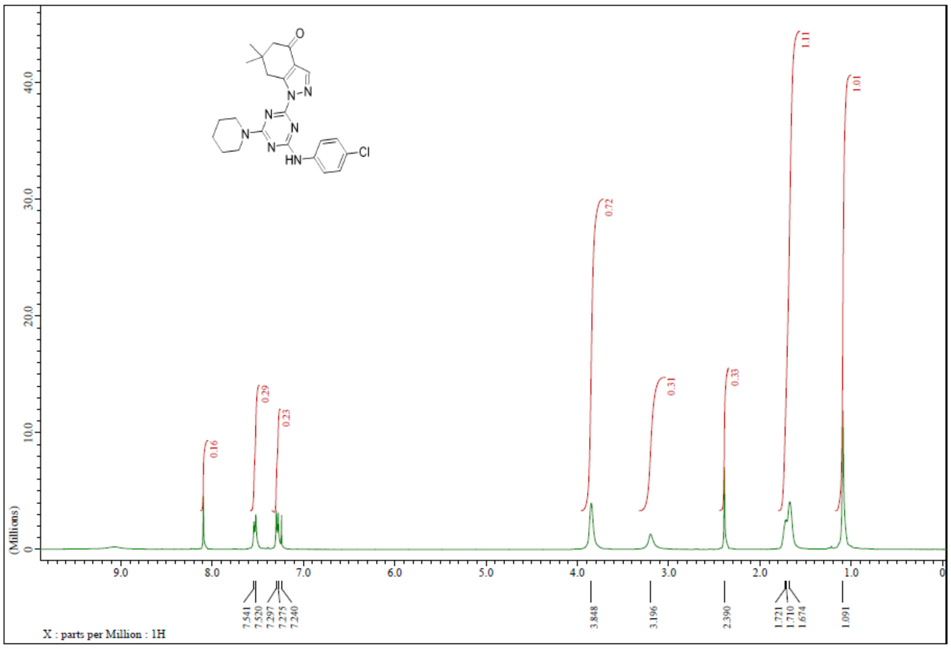

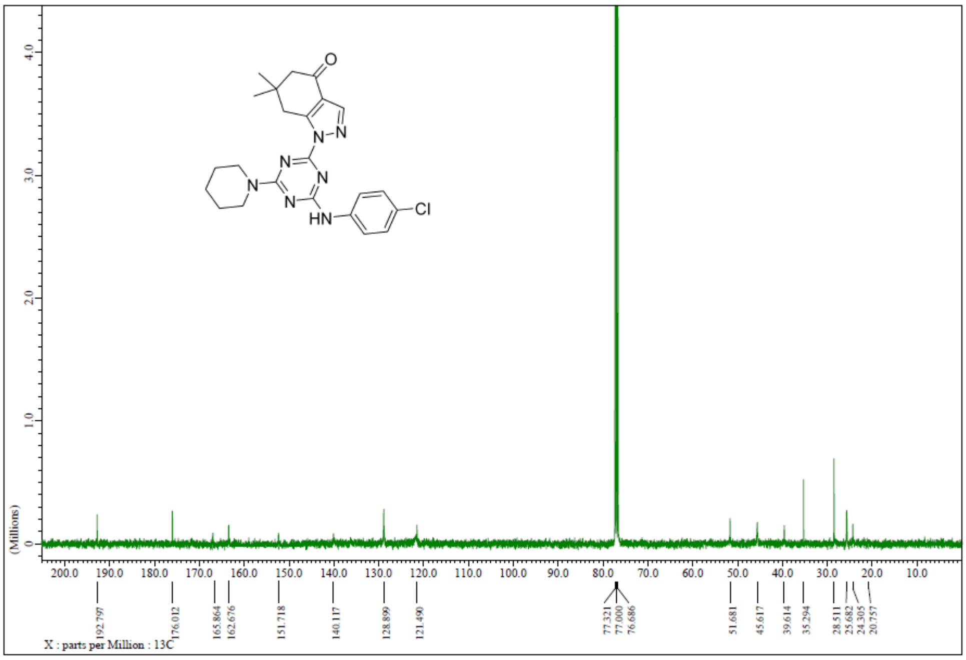

Figure S17. ^{1}H and ^{13}C compound 7h

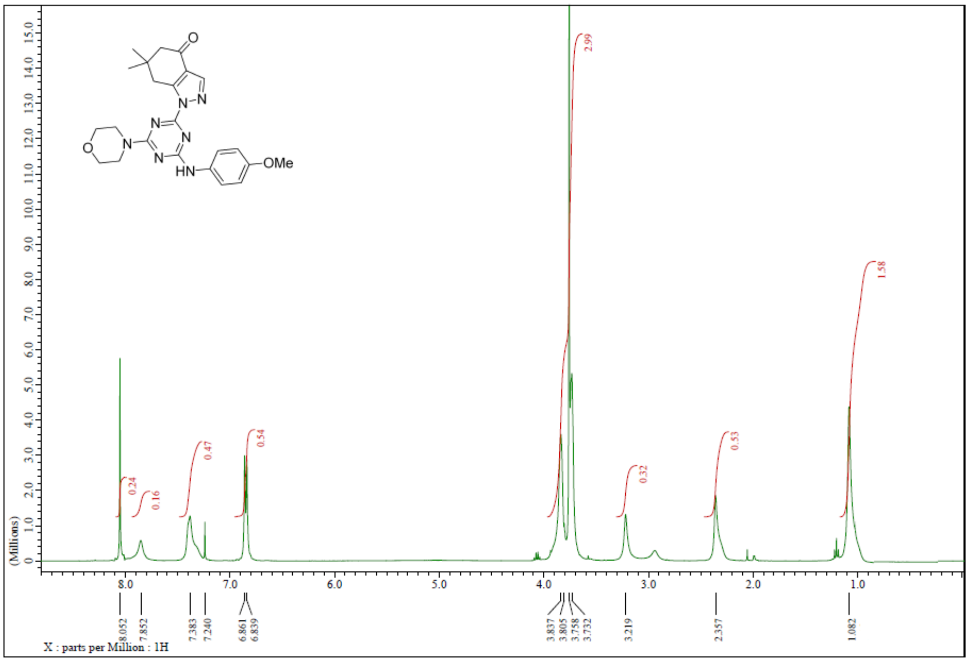

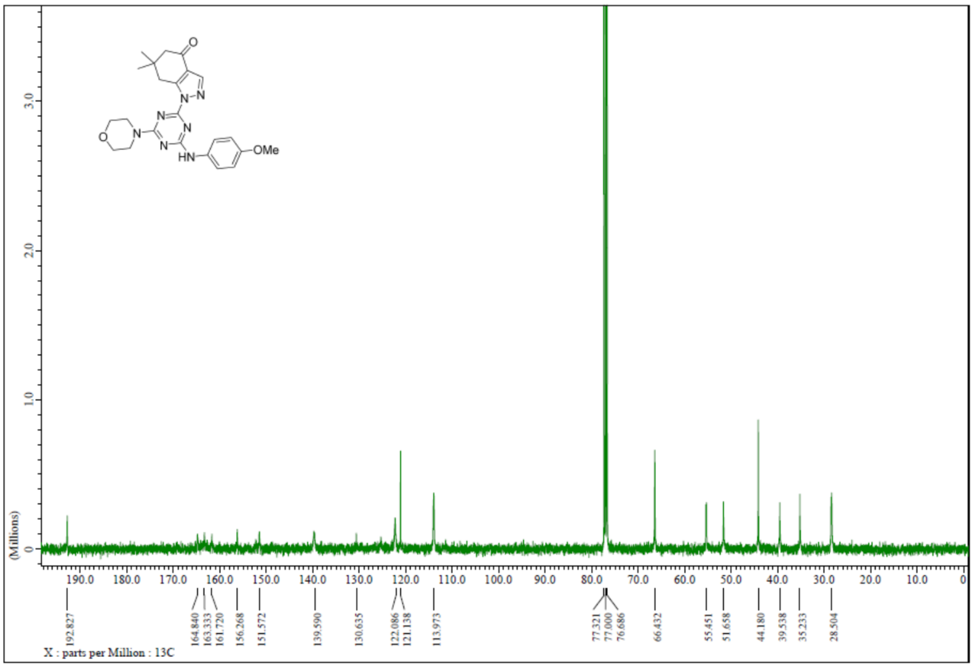

Figure S18. ^{1}H and ^{13}C compound 7i

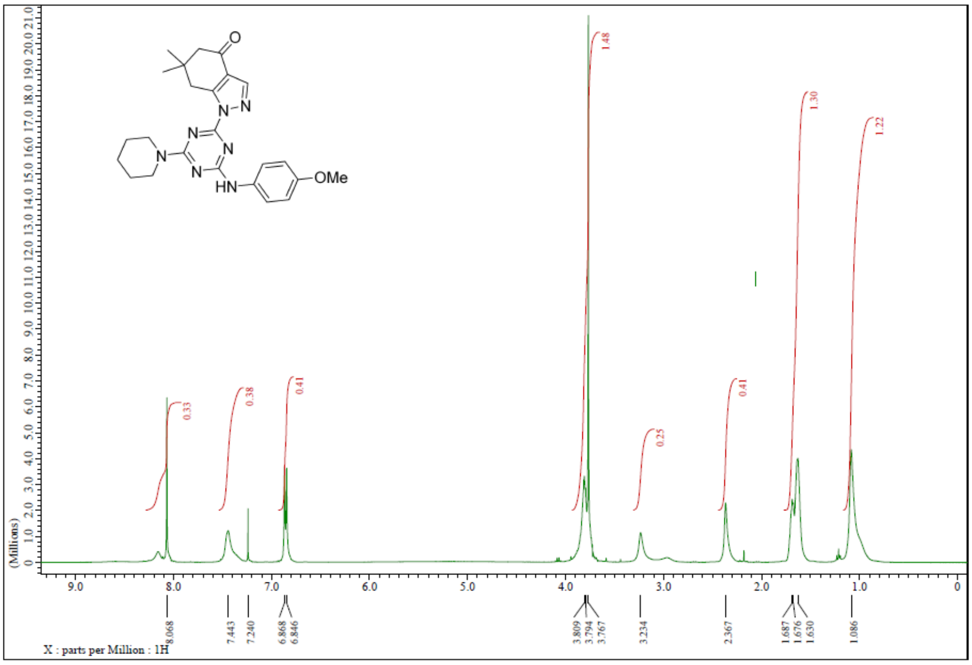

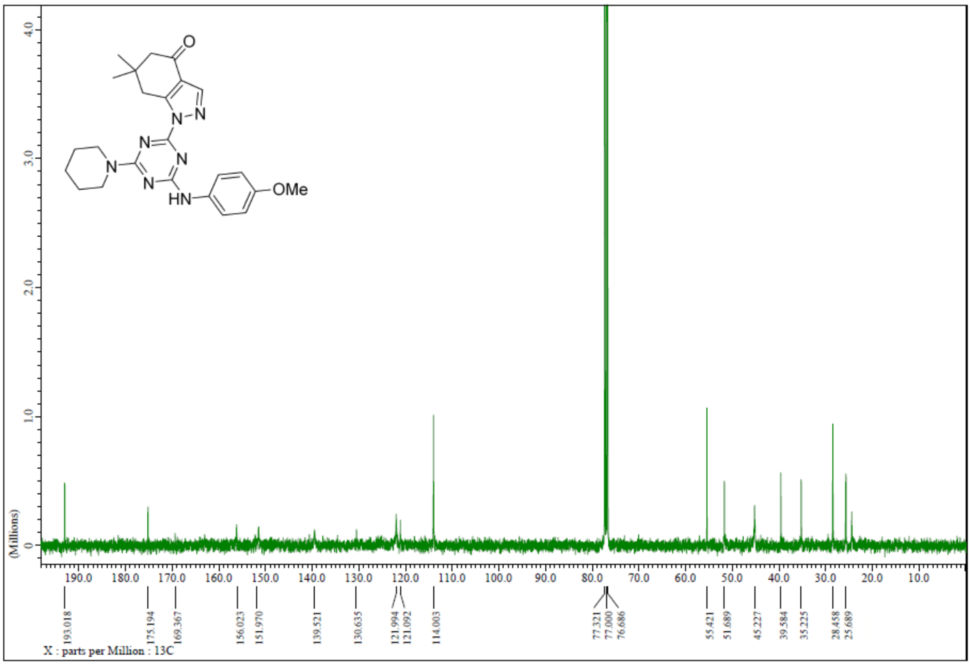

Figure S19. ^{1}H and ^{13}C compound 7j

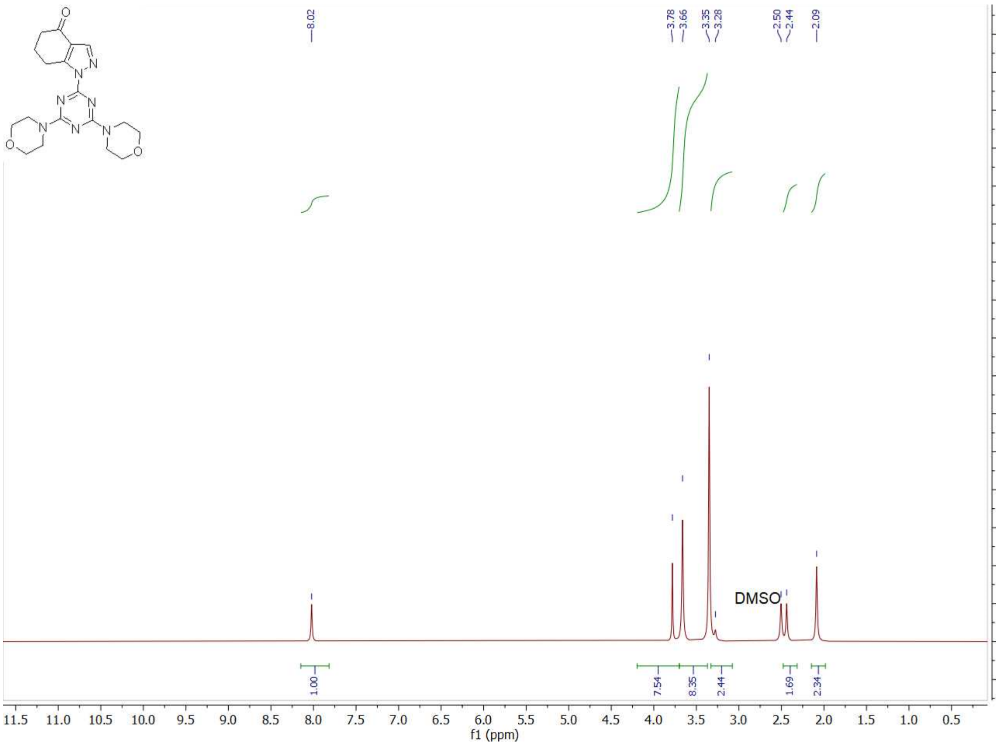

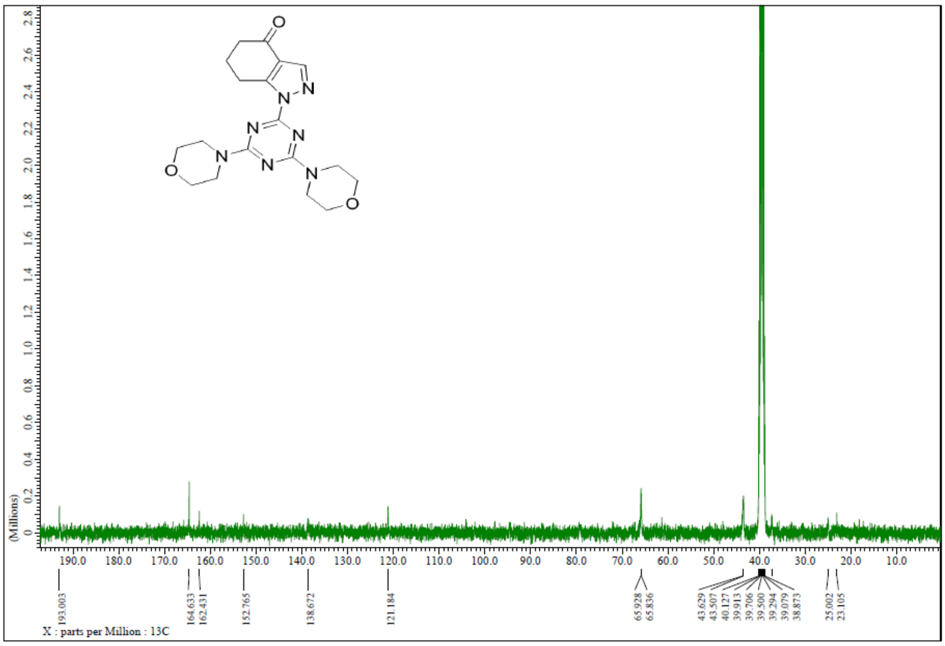

Figure S20. [1]H and ^{13}C compound 7k

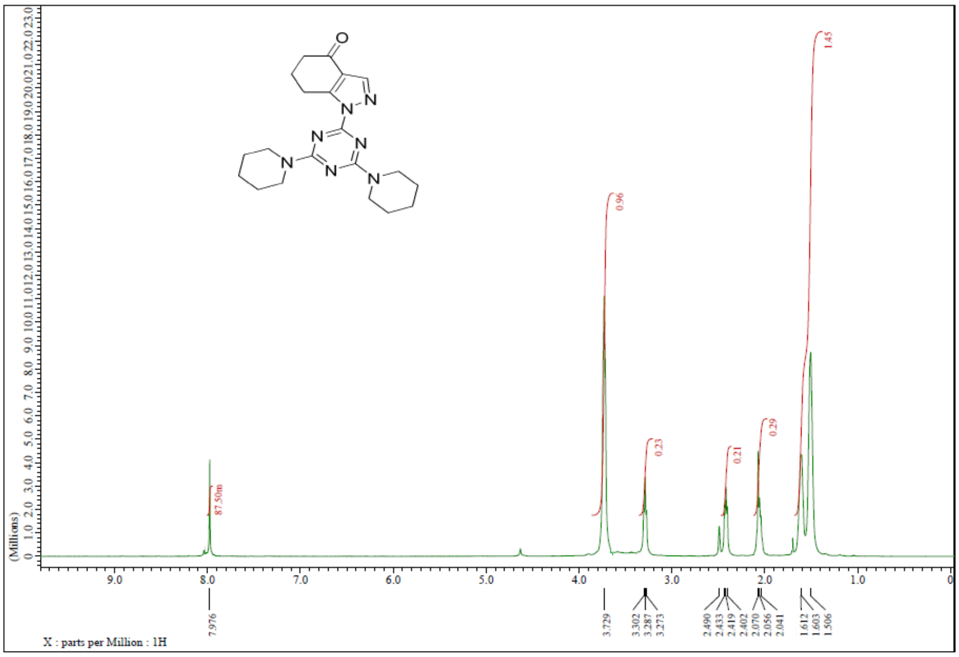

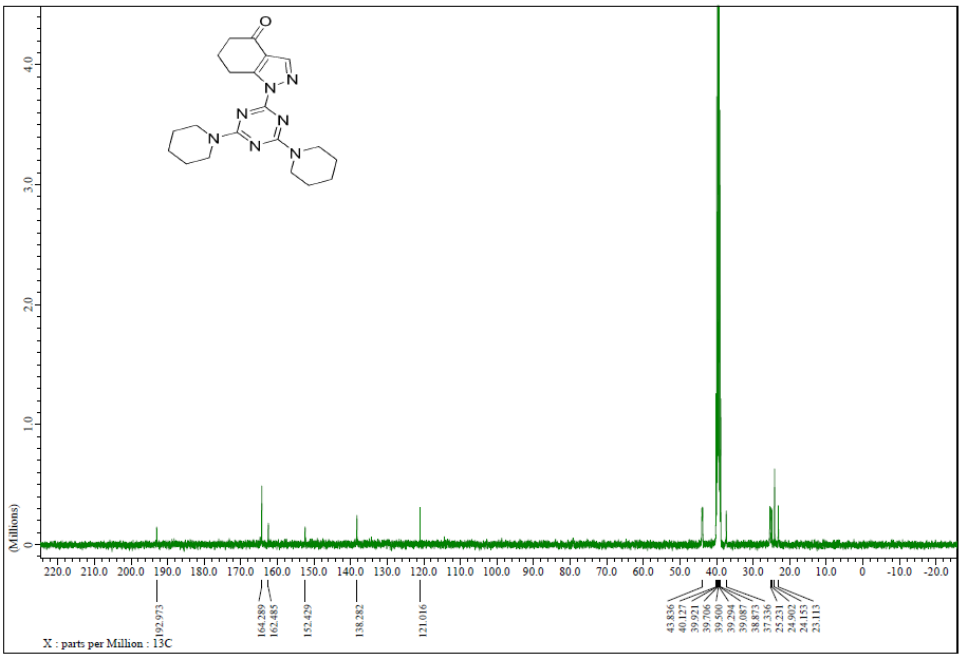

Figure S21. ^{1}H and ^{13}C compound 7l

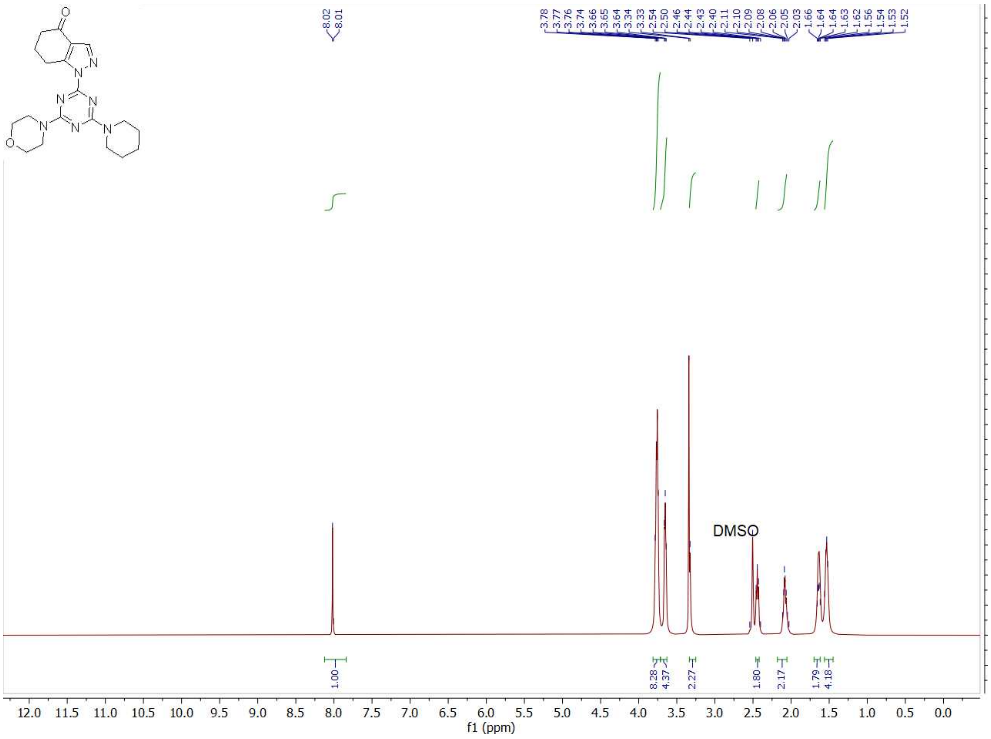

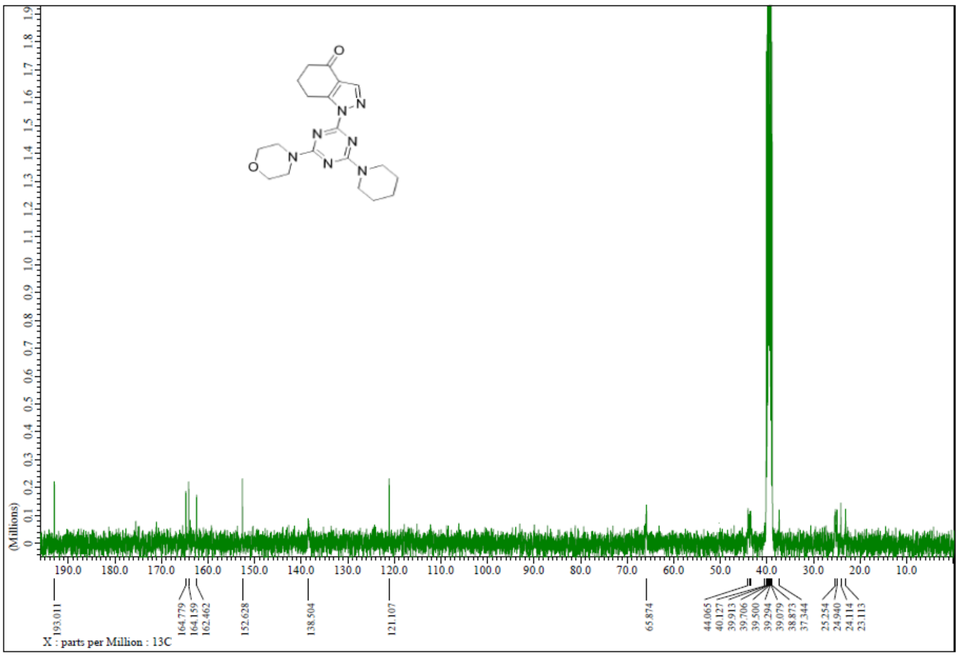

Figure S22. [1]H and ^{13}C compound 7m

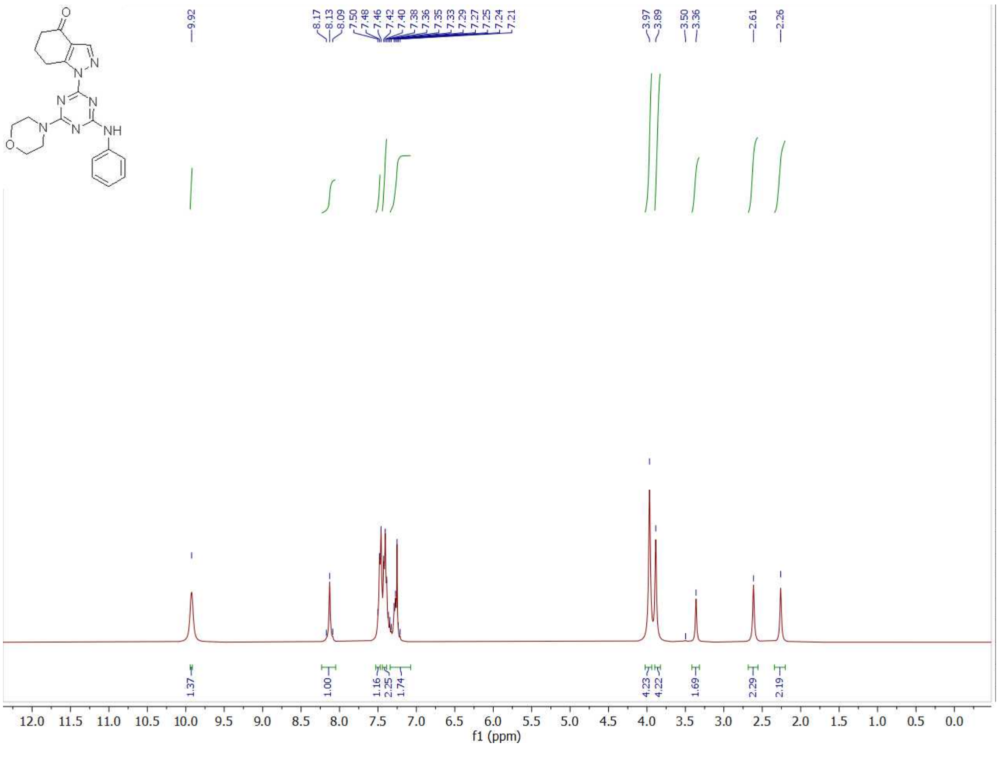

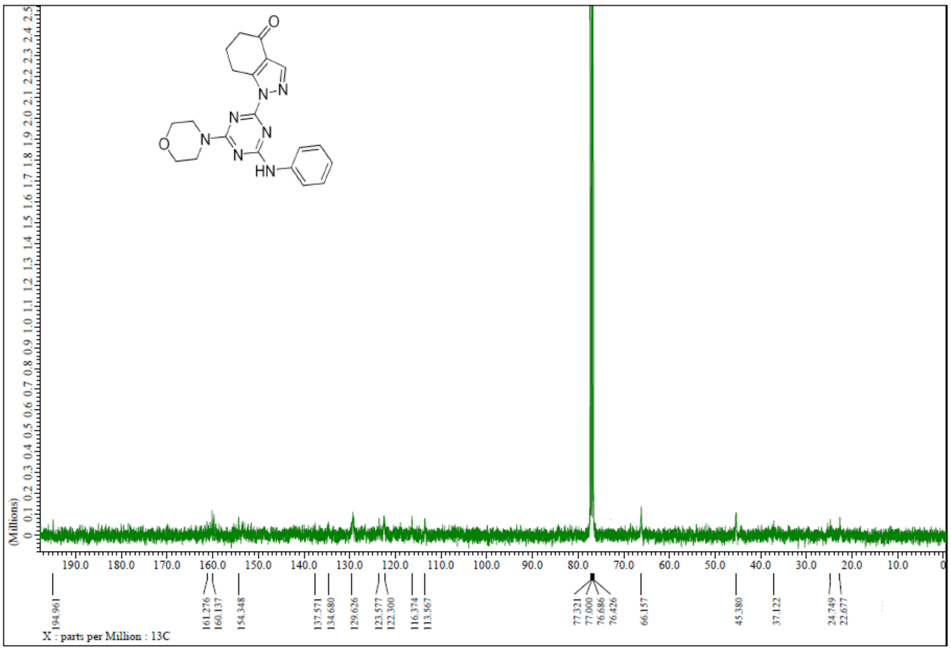

Figure S23. ¹H and ¹³C compound 7n

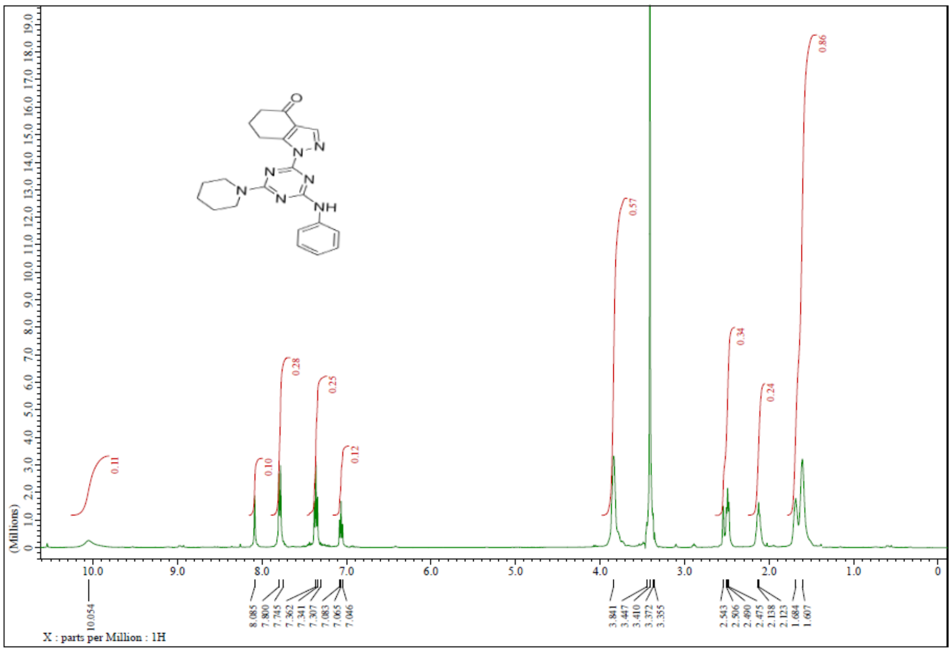

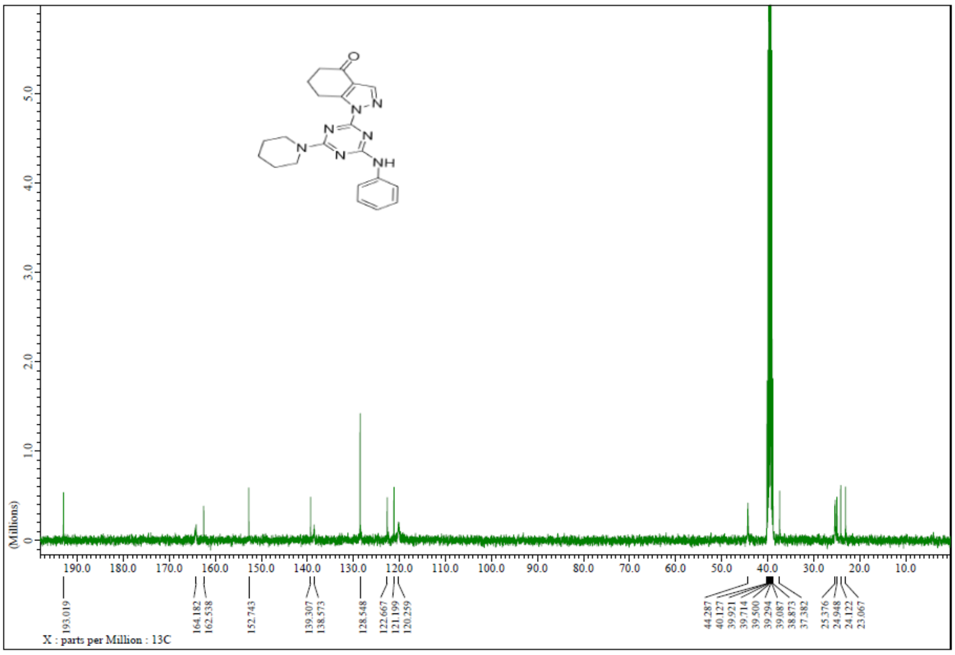

Figure S24. ^{1}H and ^{13}C compound 7o

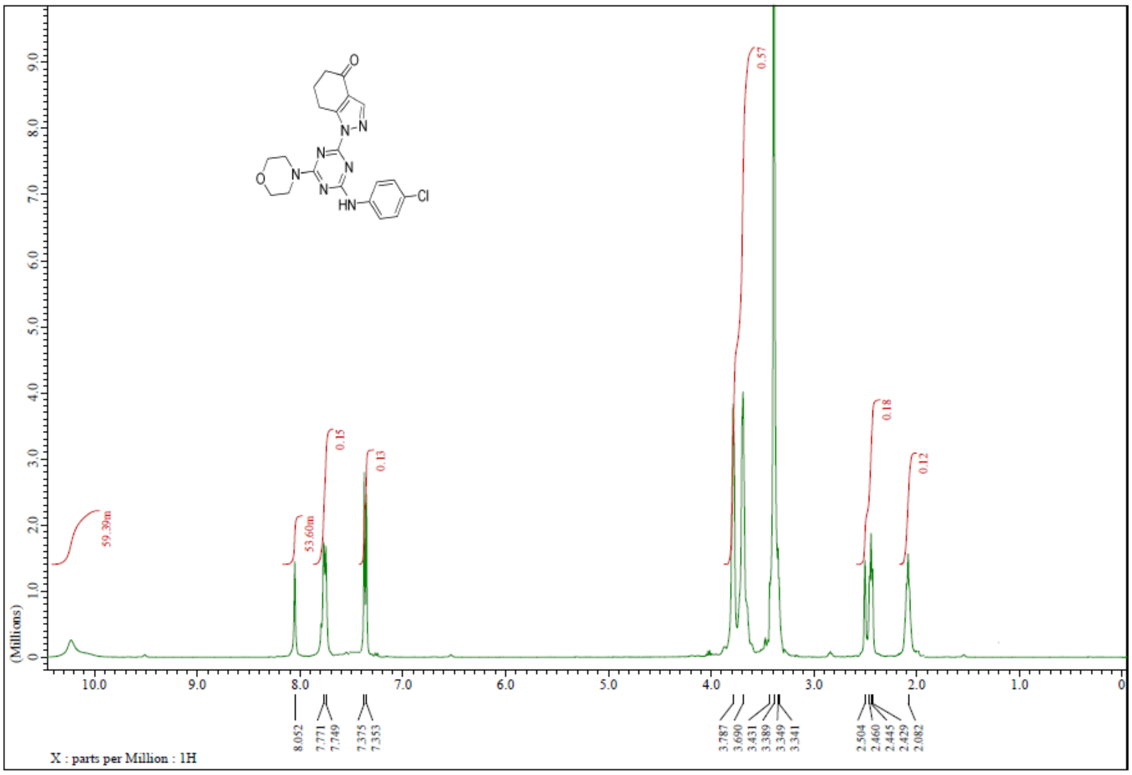

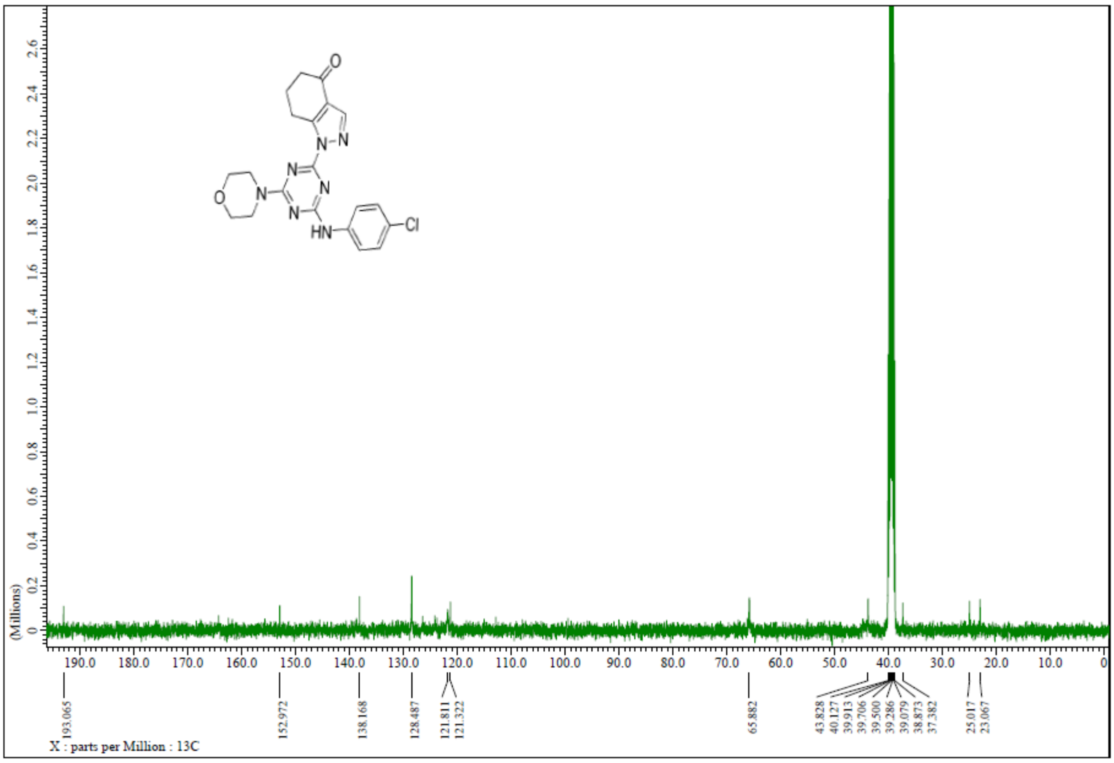

Figure S25. ^{1}H and ^{13}C compound 7p

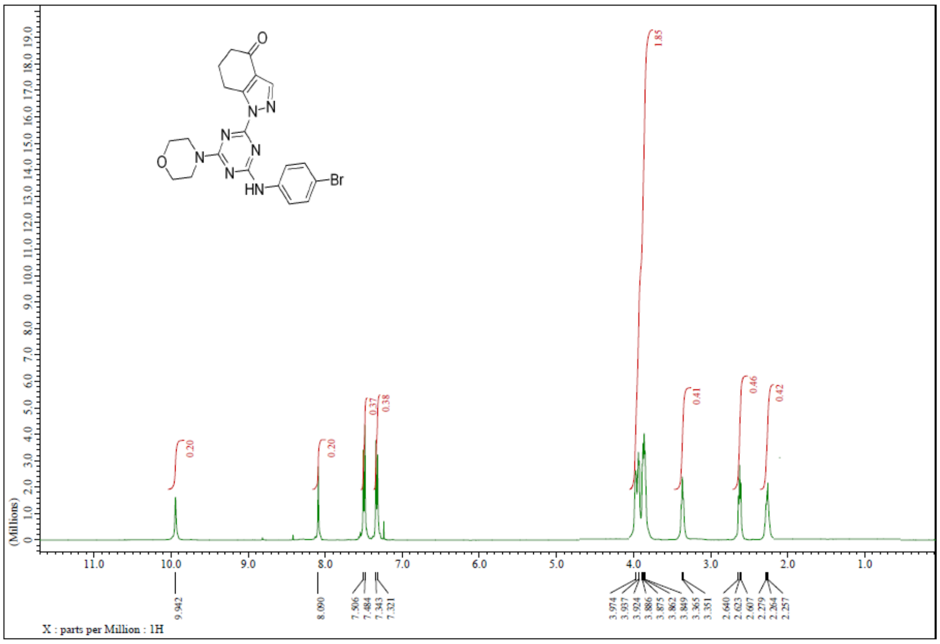

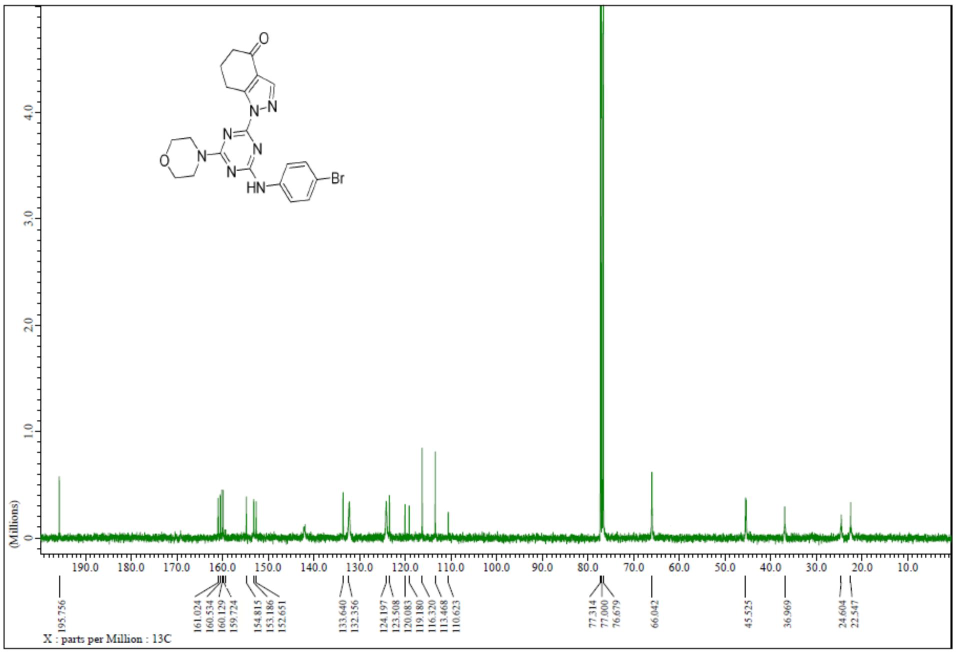

Figure S26. [1]H and ^{13}C compound 7q

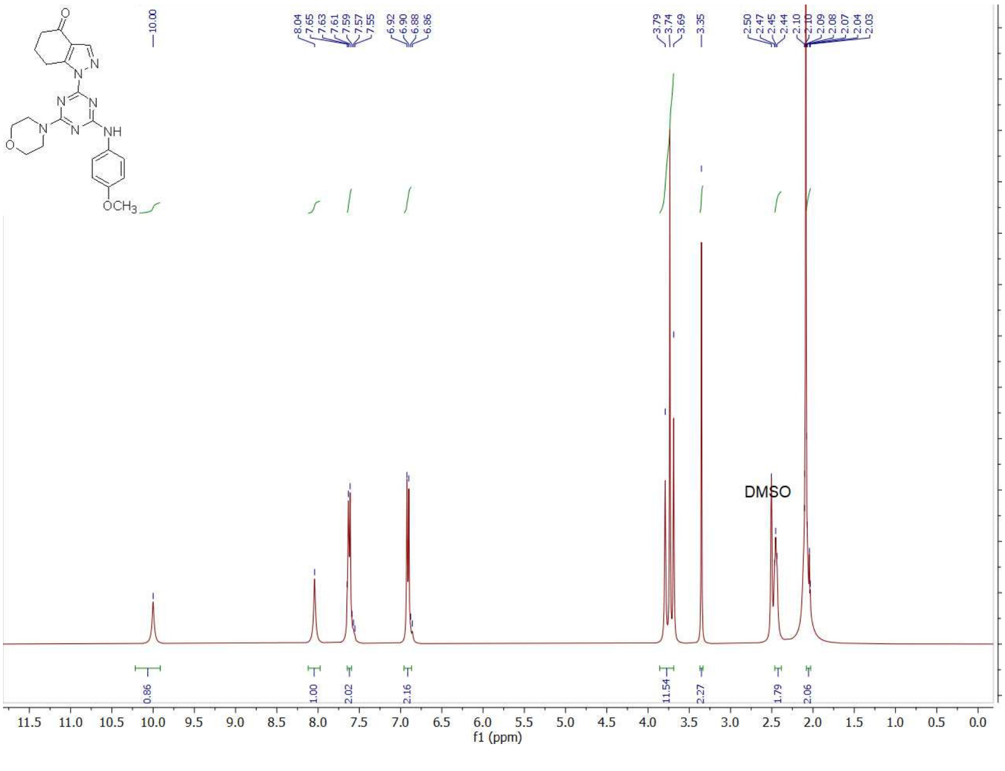

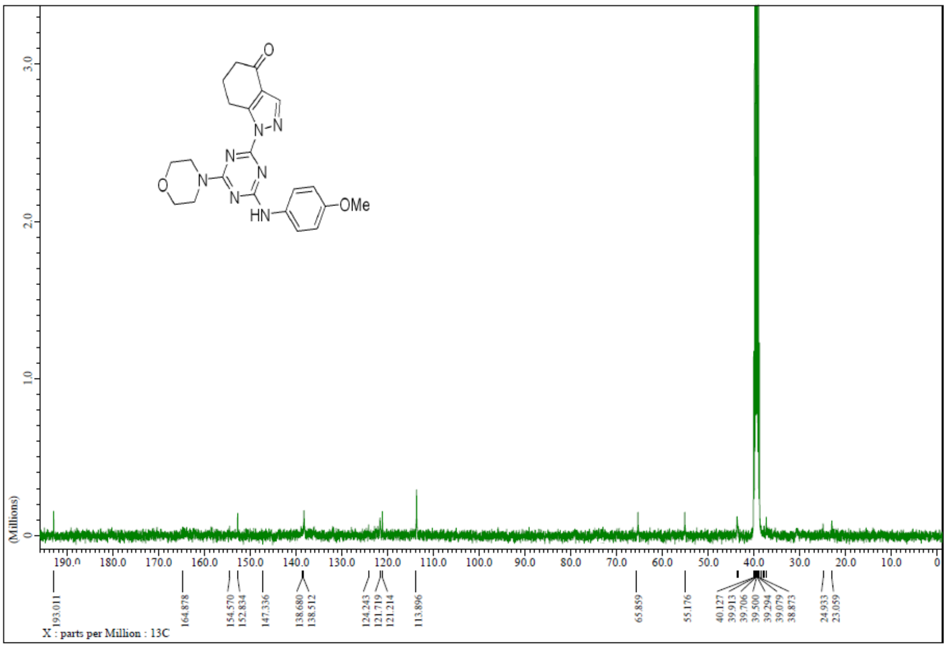

Figure S27. ^{1}H and ^{13}C compound 7r

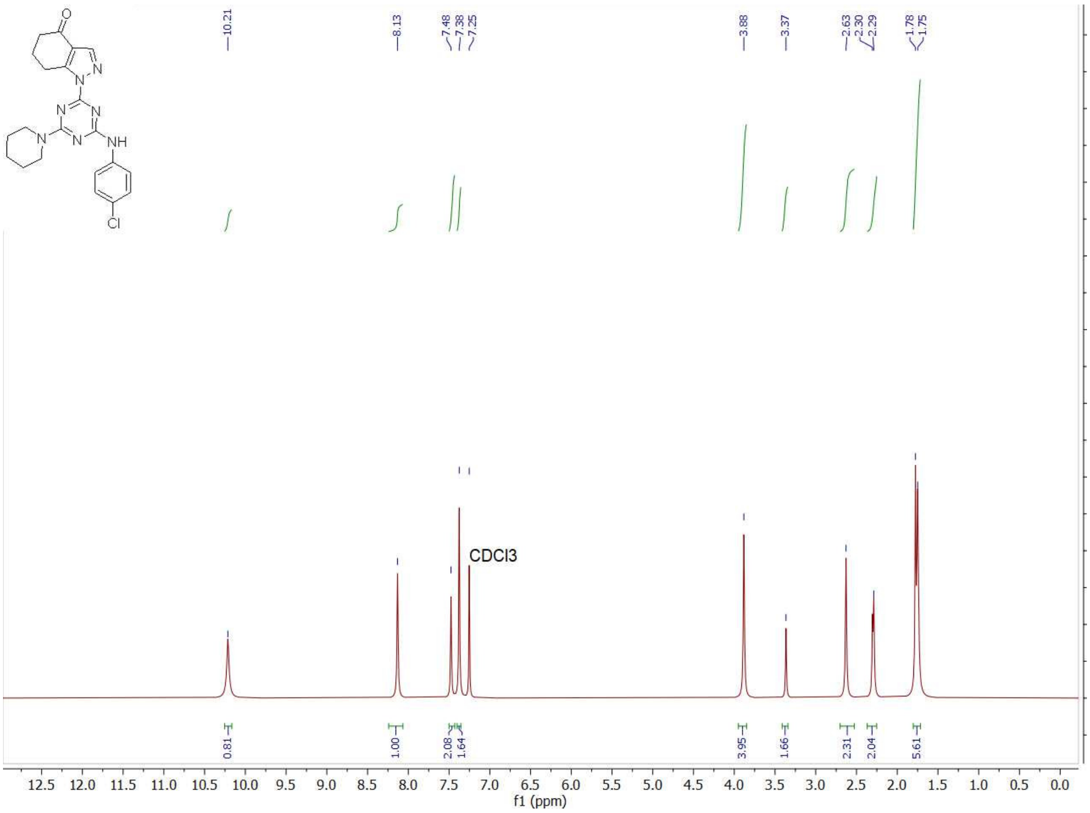

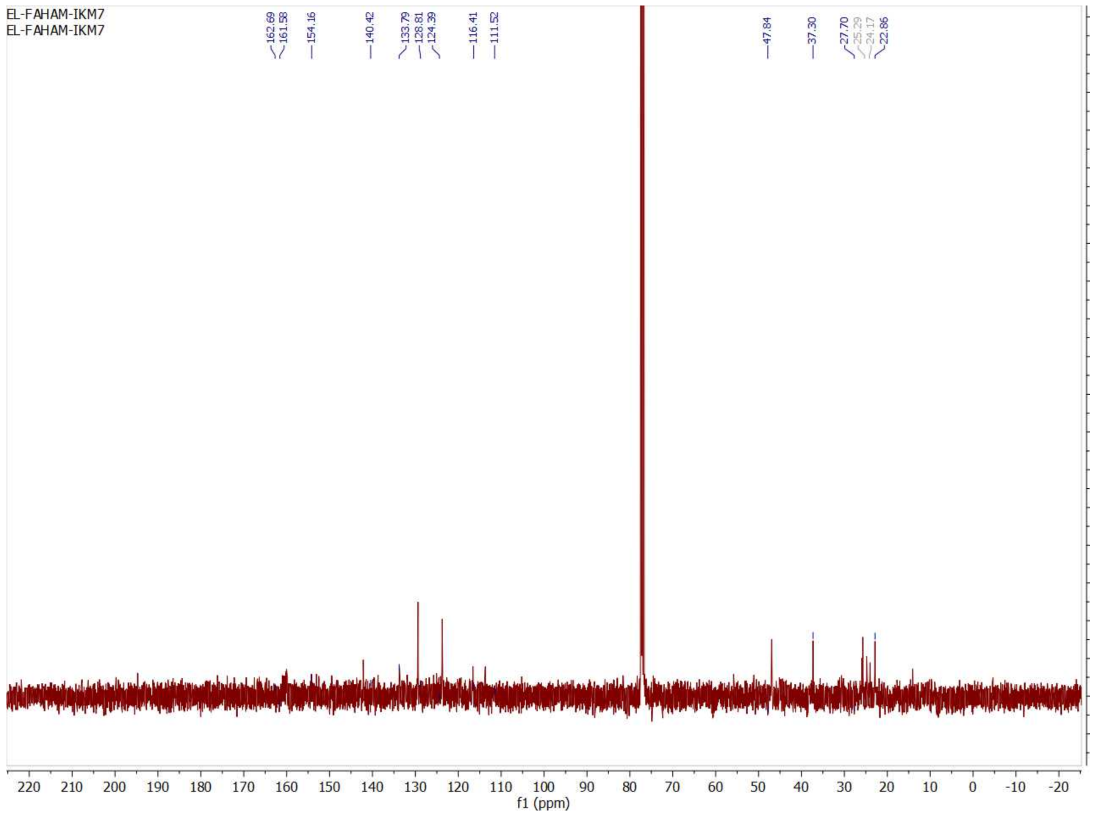

Figure S28. ^{1}H and ^{13}C compound 7s

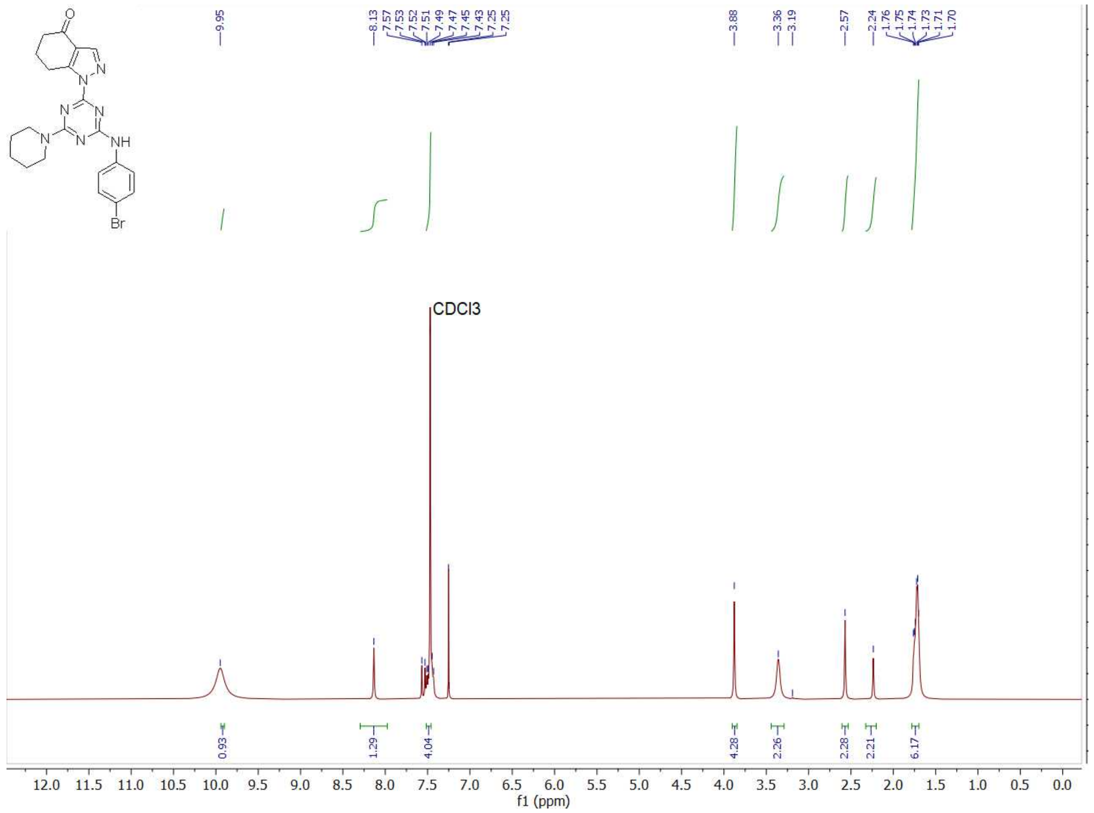

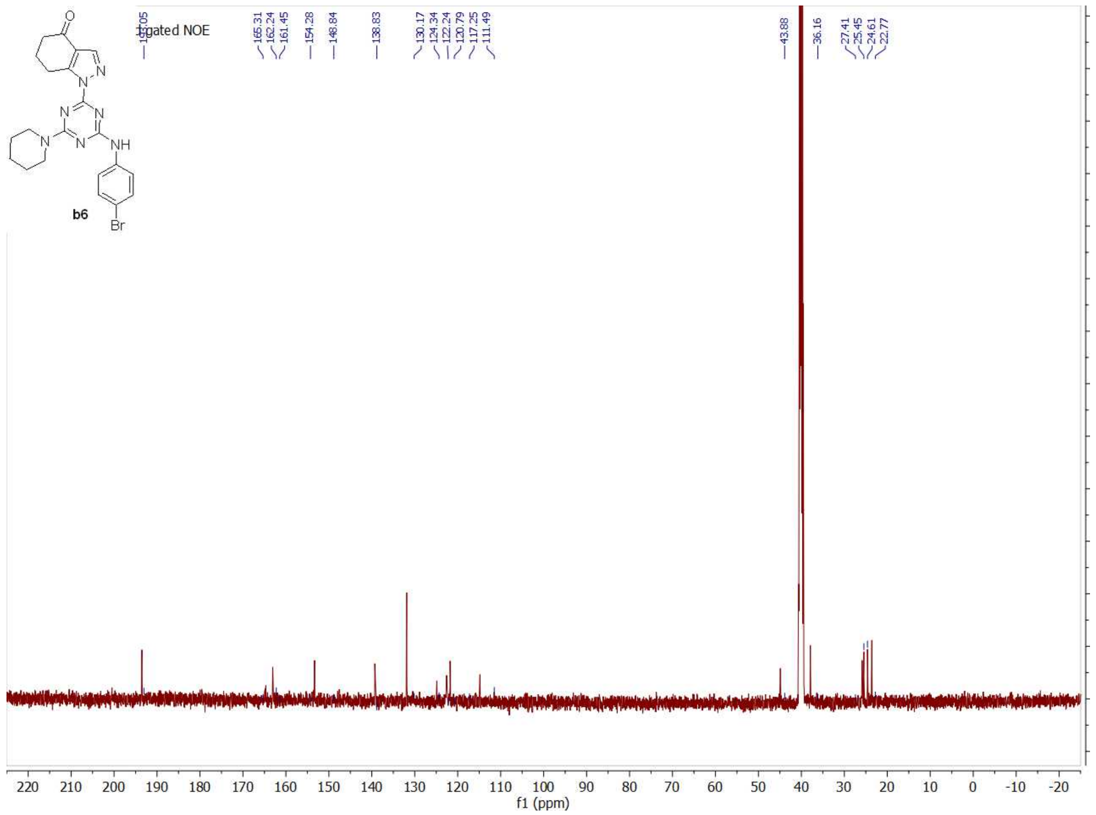

Figure S29. ^{1}H and ^{13}C compound 7t

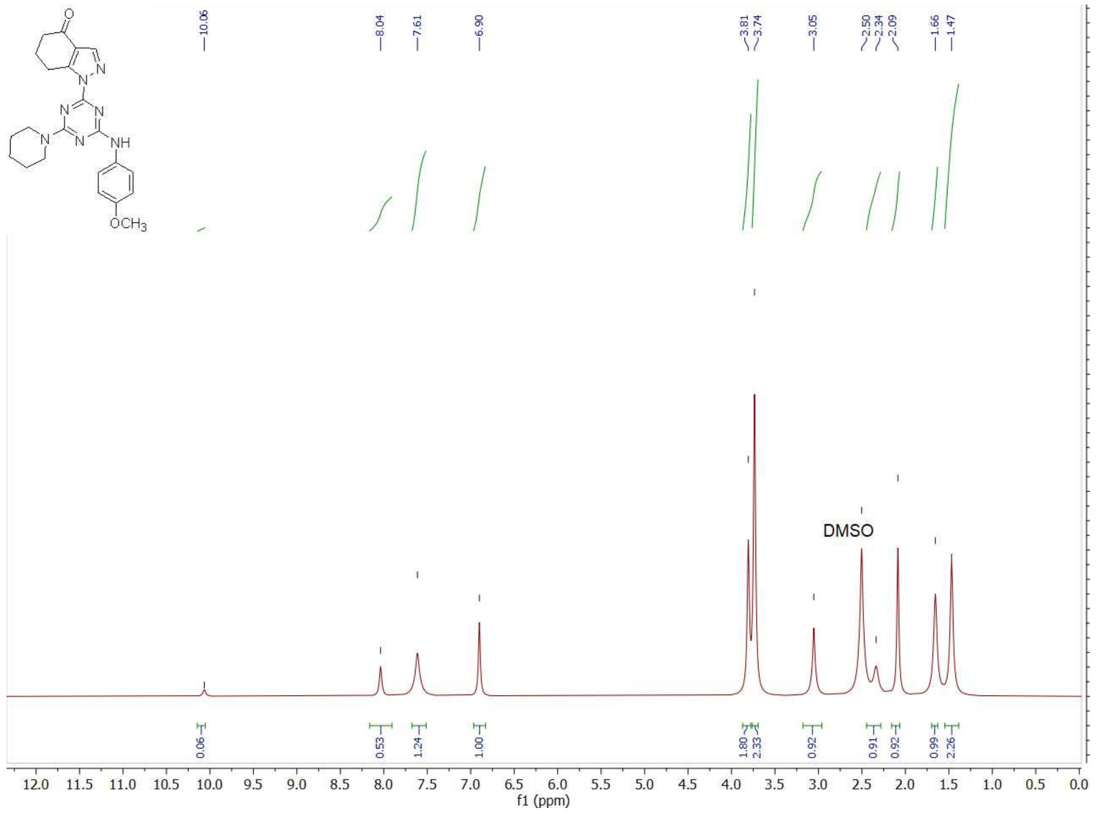

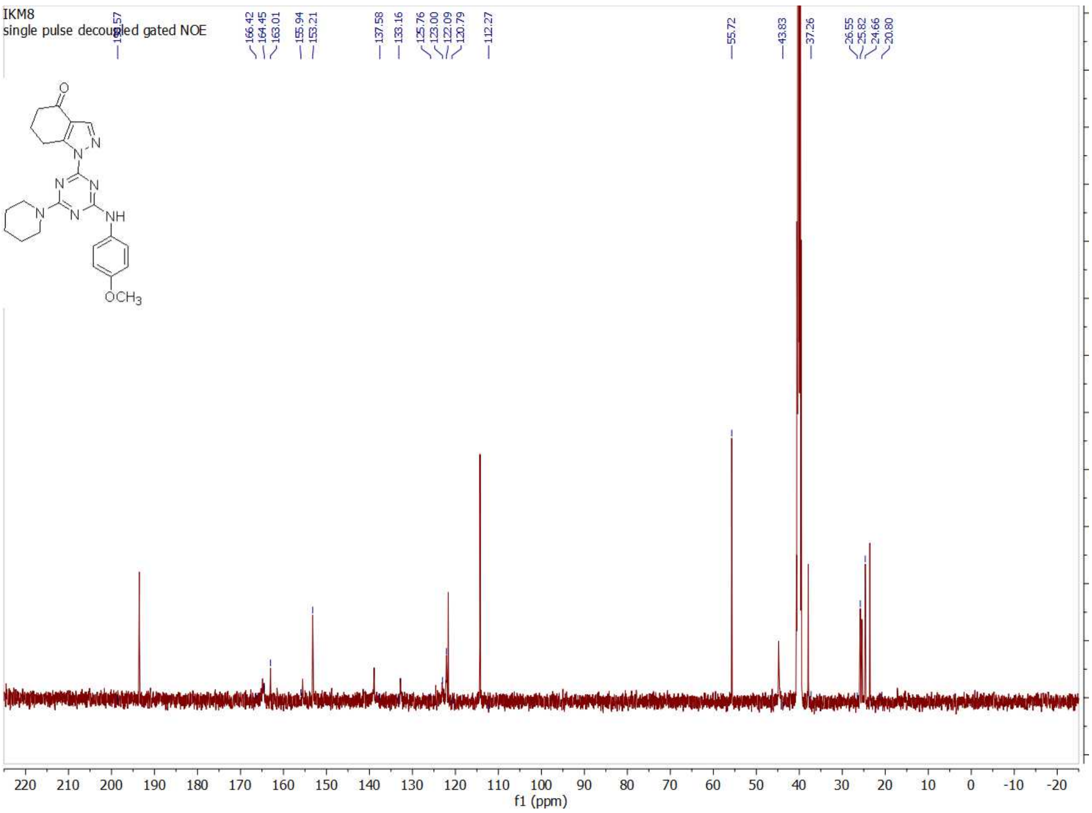

Figure S30. HRMS analysis for compound 5a

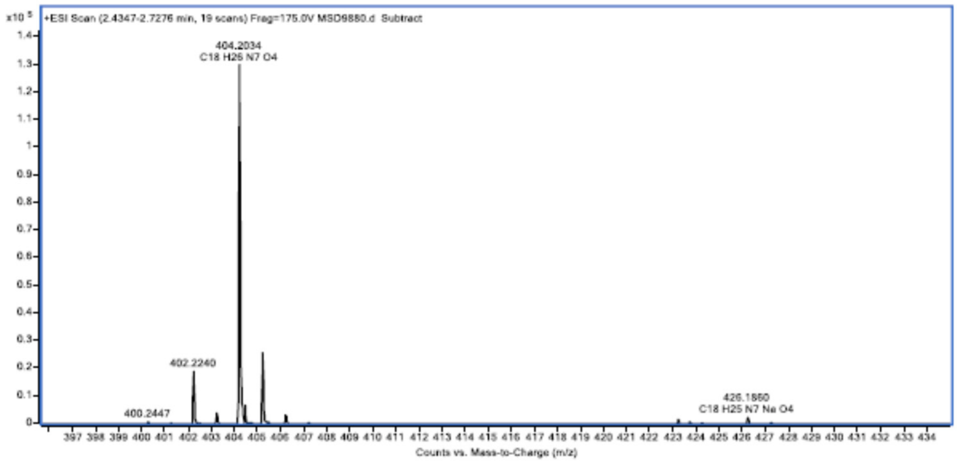

Figure S31. HRMS analysis for compound 5b

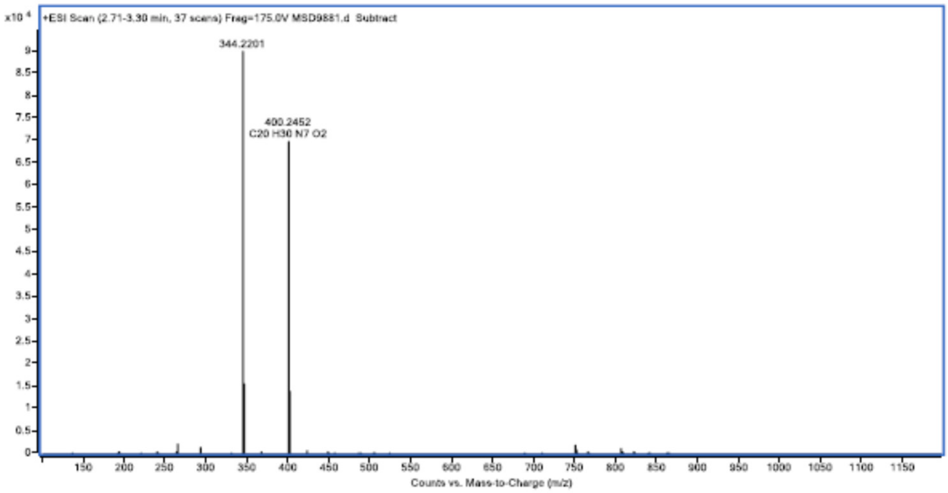

Figure S32. HRMS analysis for compound 5c

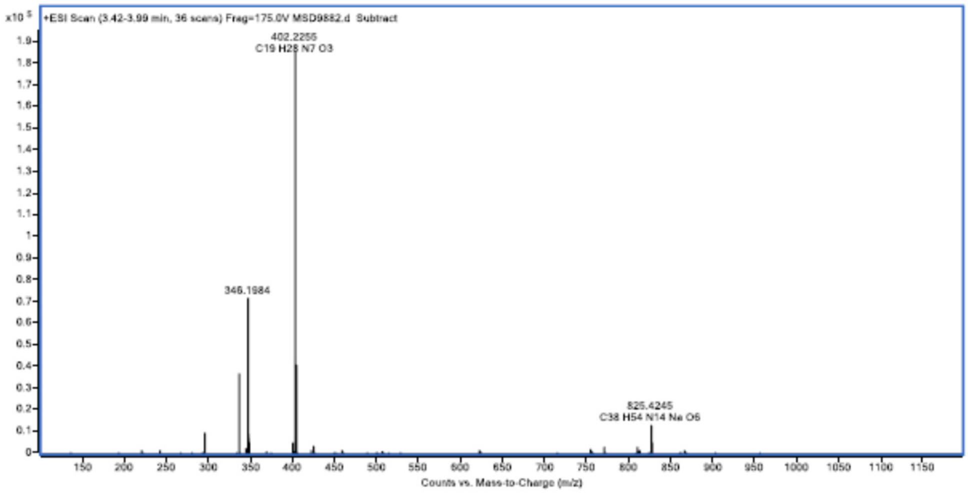

Figure S33. HRMS analysis for compound 5f

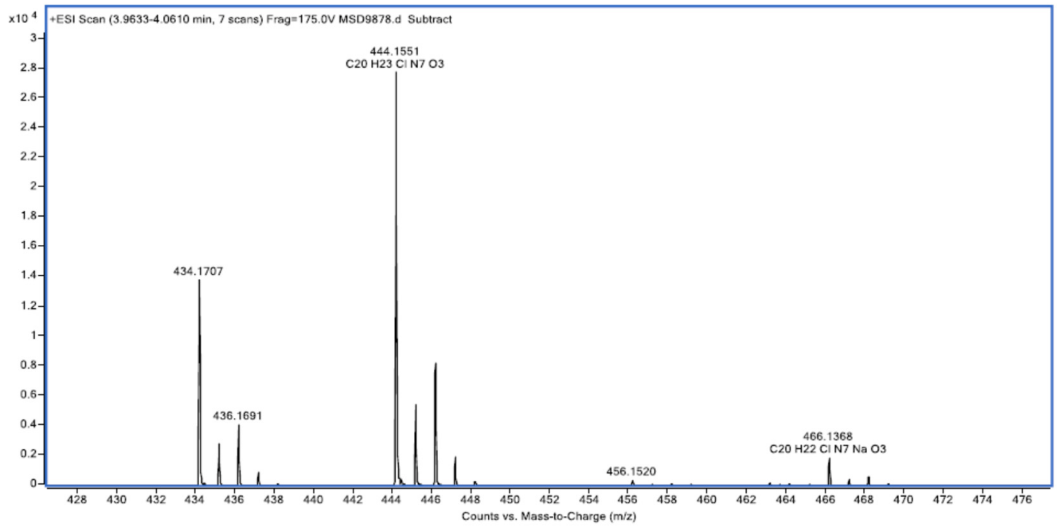

Figure S34. HRMS analysis for compound 7a

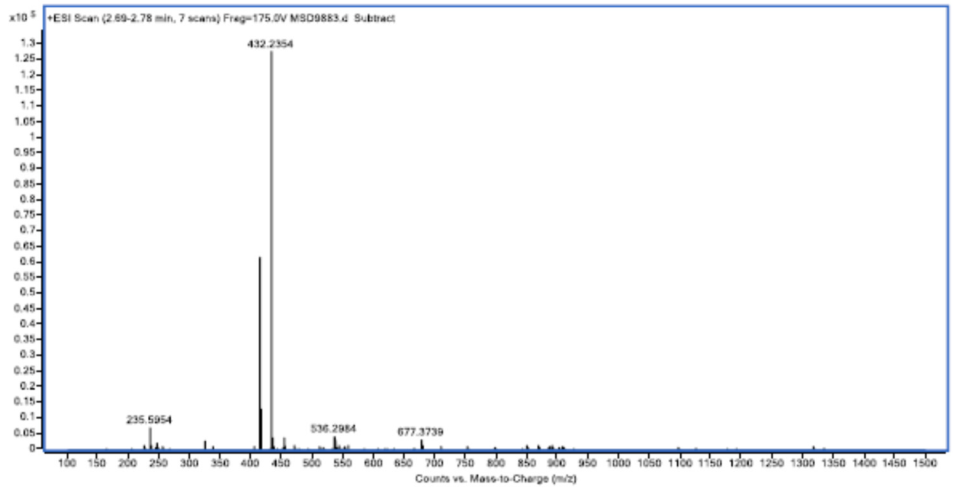

Figure S35. HRMS analysis for compound 7b

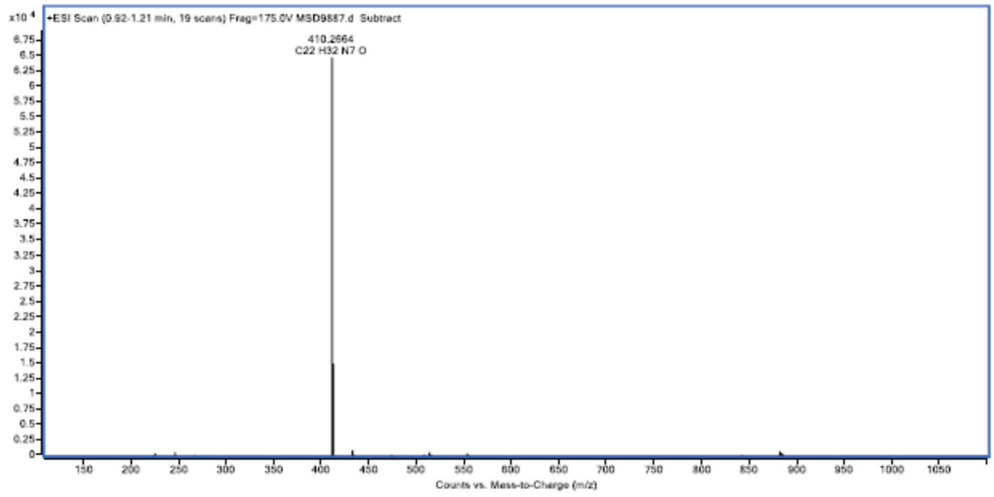

Figure S36. HRMS analysis for compound 7c

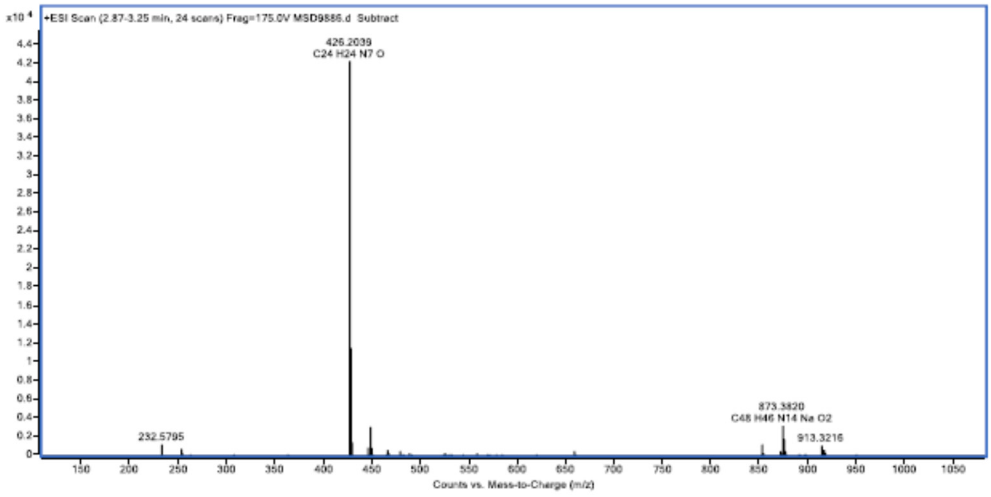

Figure 37. HRMS analysis for compound 7h

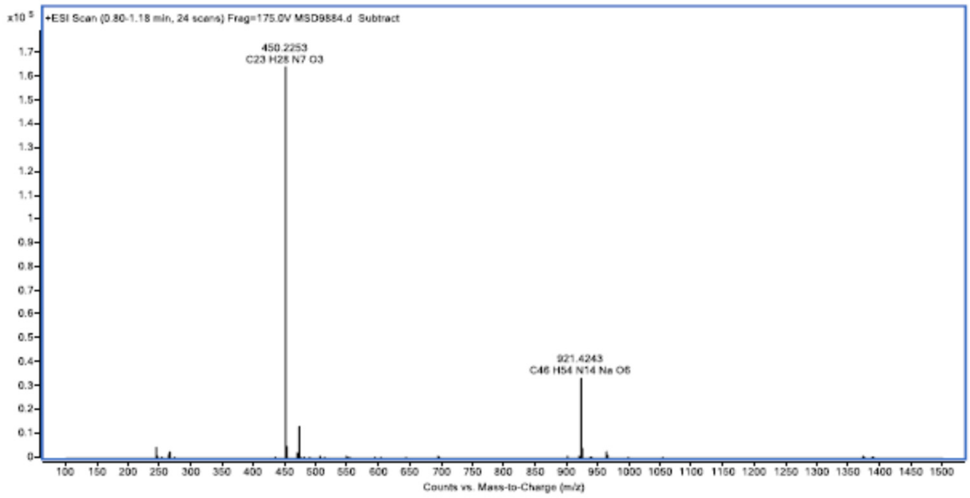

Figure S38. HRMS analysis for compound 7i

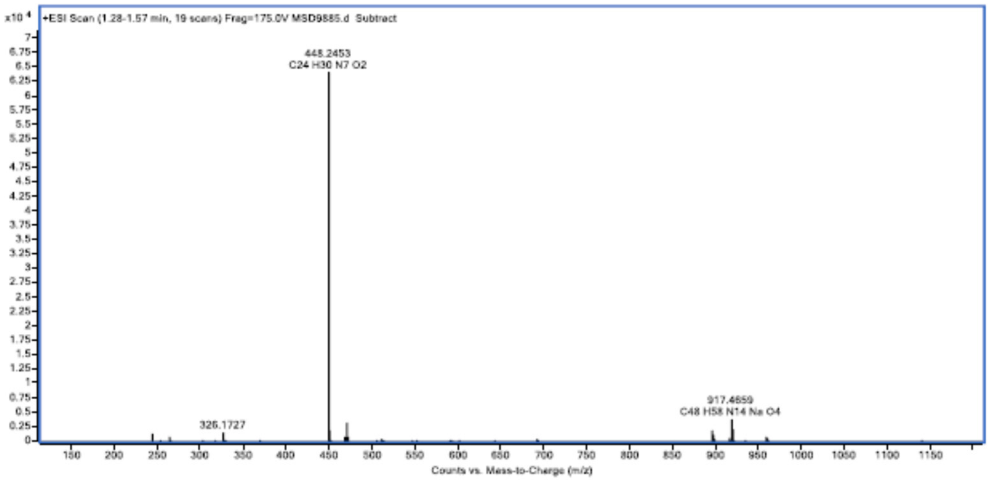

3. **Flow cytometric analysis (Annexin V-FTIC/PI assay)**

MDA-MB-231

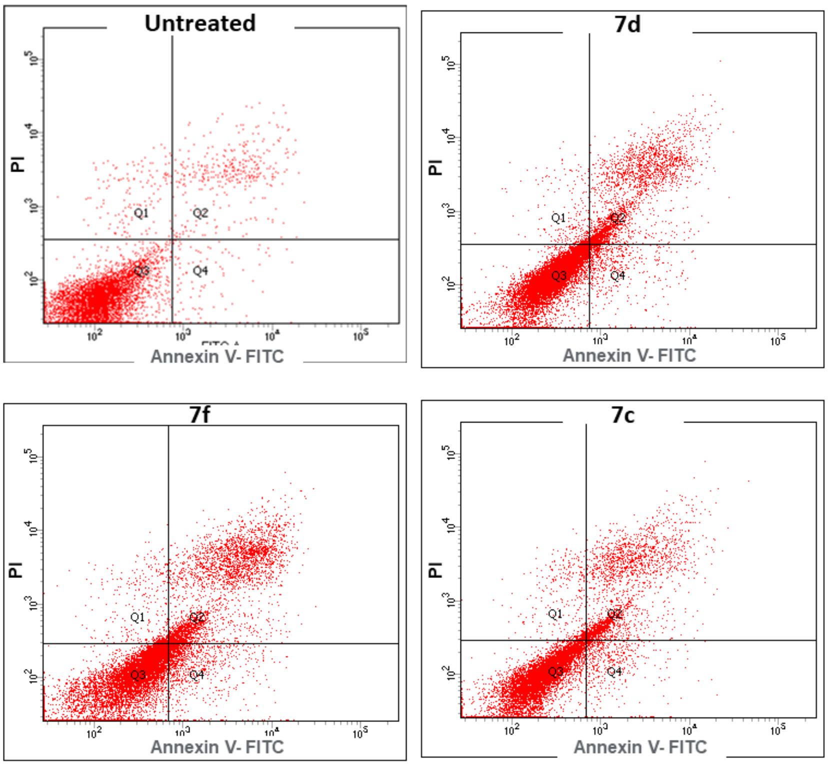

MCF-7

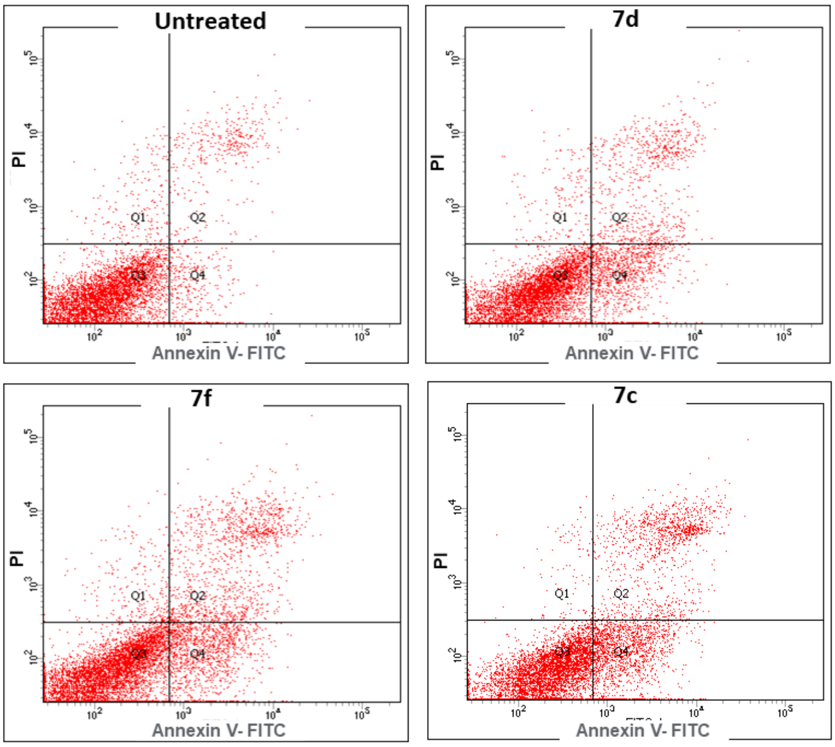

U-87 MG

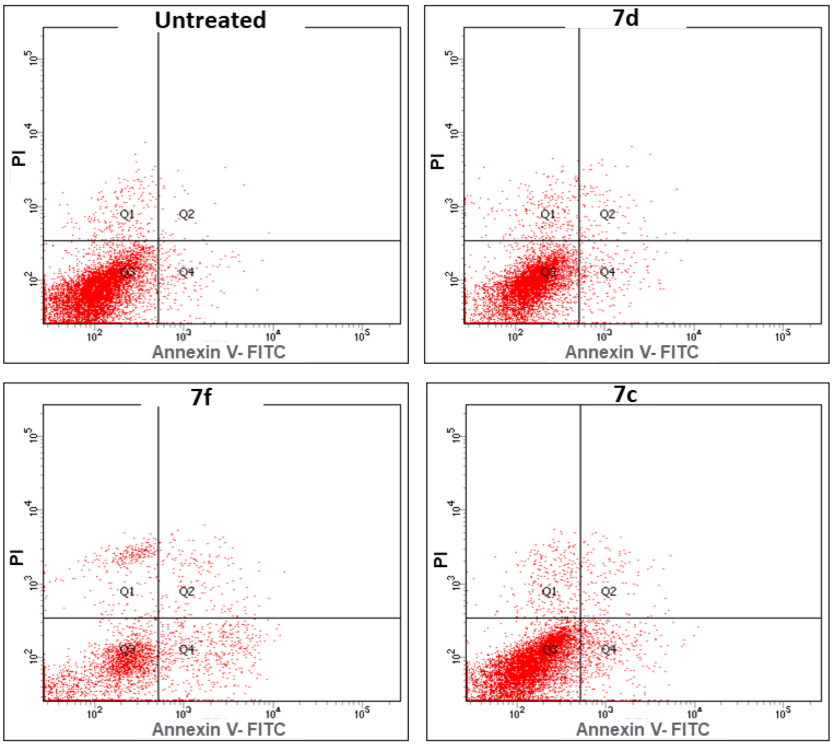
Untreated
7d
7f
7c
PI
Annexin V- FITC
Q1
Q2
Q3
Q4

A549

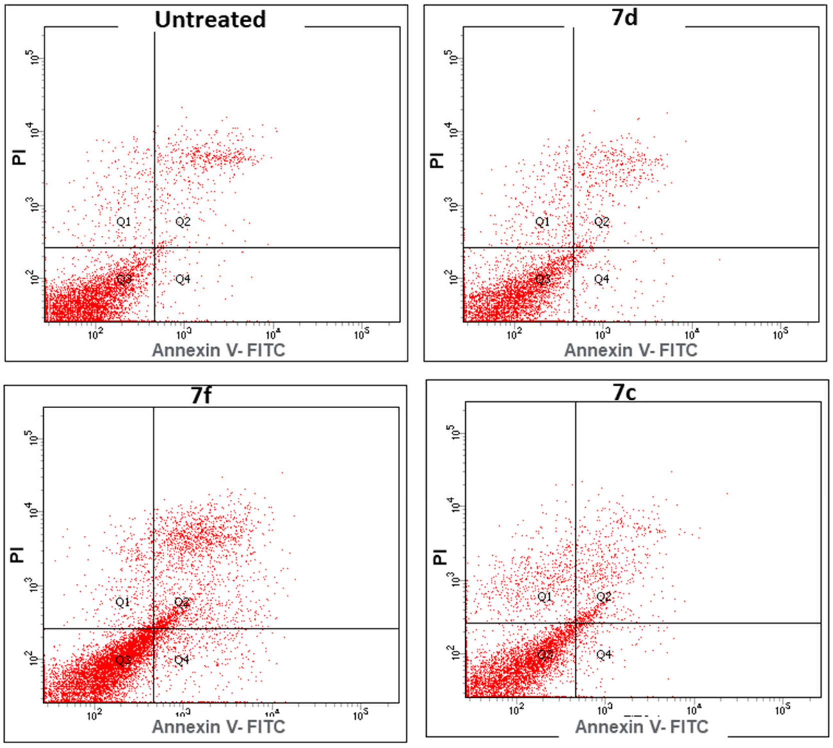
Untreated
7d
7f
7c
PI
Annexin V- FITC
Q1
Q2
Q3
Q4

PANC-1

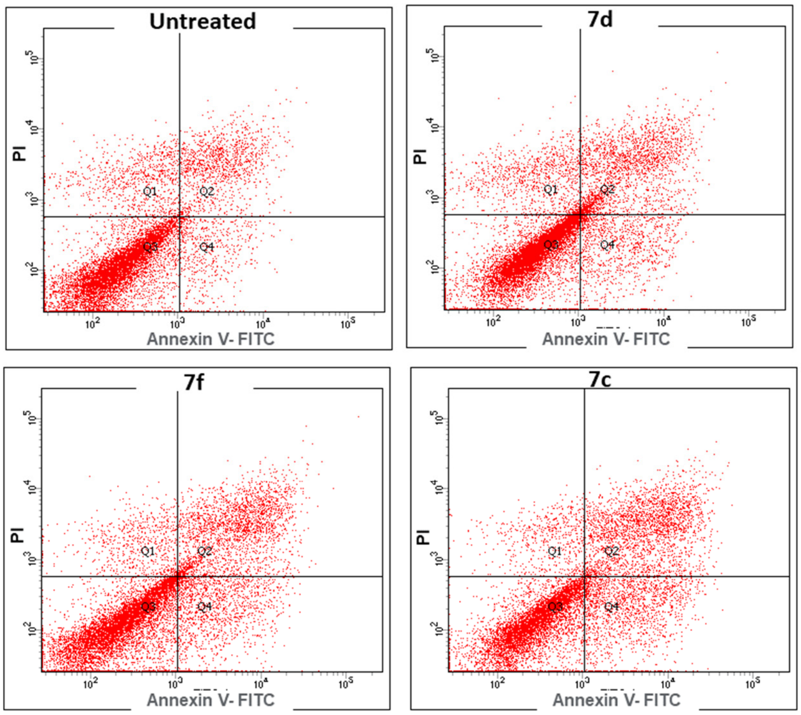

HDF

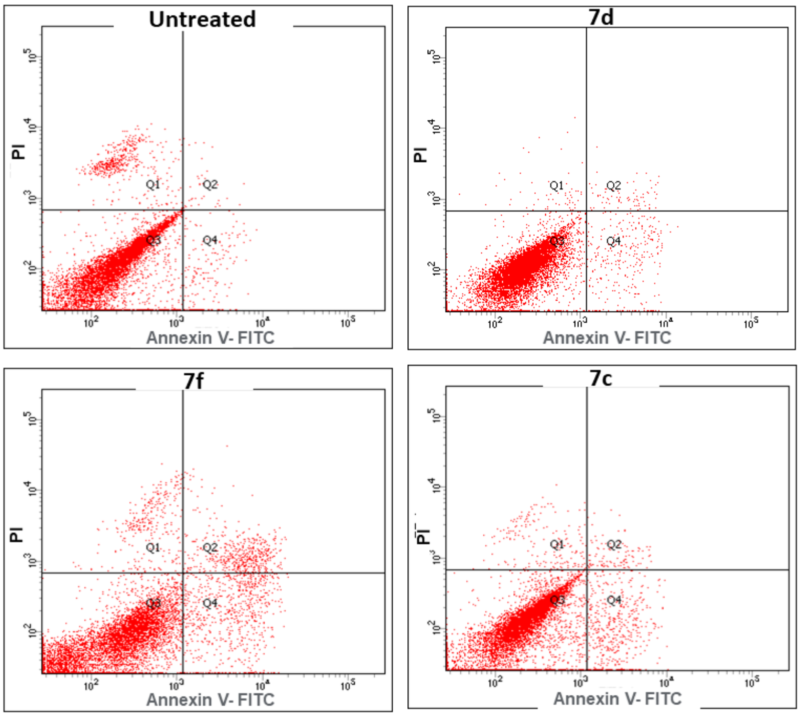

Figure S39: Flow cytometric analysis (Annexin V-FTIC/PI assay) MDA-MB-231, MCF-7, U87 MG, A459 and PANC1 cancer cell lines compared to normal cell line HDF for 24 h **7d, 7f,** and **7c**. The represented dot plots showing percentage of viable, early apoptotic, late apoptotic, and necrotic cells. in. The results showed a higher percentage in the apoptosis mechanism induced in all cancer cell lines compared to the normal cell line.
